# Real-world treatment patterns and clinical outcomes for inpatients with COVID-19 in the US from September 2020 to February 2021

**DOI:** 10.1371/journal.pone.0261707

**Published:** 2021-12-28

**Authors:** Olulade Ayodele, Kaili Ren, Jing Zhao, James Signorovitch, Michele Jonsson Funk, Julia Zhu, Ying Bao, Kathleen Gondek, Hillary Keenan, for the R&D COVID Alliance

**Affiliations:** 1 Data Sciences Institute, Takeda Pharmaceutical Company Limited, Cambridge, MA, United States of America; 2 Analysis Group, Data Sciences Institute, Boston, MA, United States of America; 3 Department of Epidemiology, University of North Carolina, Chapel Hill, NC, United States of America; 4 Center for Observational Research and Data Science, Bristol-Myers Squibb, Princeton, NJ, United States of America; Ohio State University Wexner Medical Center Department of Surgery, UNITED STATES

## Abstract

The objective of this retrospective cohort study was to describe pre-treatment characteristics, treatment patterns, health resource use, and clinical outcomes among adults hospitalized with COVID-19 in the United States (US) who initiated common treatments for COVID-19. The Optum^®^ COVID-19 electronic health records database was used to identify patients >18 years, diagnosed with COVID-19, who were admitted to an inpatient setting and received treatments of interest for COVID-19 between September 2020 and January 2021. Patients were stratified into cohorts based on index treatment use. Patient demographics, medical history, care setting, medical procedures, subsequent treatment use, patient disposition, clinical improvement, and outcomes were summarized descriptively. Among a total of 26,192 patients identified, the most prevalent treatments initiated were dexamethasone (35.4%) and dexamethasone + remdesivir (14.9%), and dexamethasone was the most common subsequent treatment. At day 14 post-index, <10% of patients received any treatments of interest. Mean (standard deviation [SD]) patient age was 65.6 (15.6) years, and the most prevalent comorbidities included hypertension (44.8%), obesity (35.4%), and diabetes (25.7%). At the end of follow-up, patients had a mean (SD) 8.1 (6.6) inpatient days and 1.4 (4.1) days with ICU care. Oxygen supplementation, non-invasive, or invasive ventilation was required by 4.5%, 3.0%, and 3.1% of patients, respectively. At the end of follow-up, 84.2% of patients had evidence of clinical improvement, 3.1% remained hospitalized, 83.8% were discharged, 4% died in hospital, and 9.1% died after discharge. Although the majority of patients were discharged alive, no treatments appeared to alleviate the inpatient morbidity and mortality associated with COVID-19. This highlights an unmet need for effective treatment options for patients hospitalized with COVID-19.

## Introduction

COVID-19 (coronavirus disease 2019), a disease caused by the novel coronavirus severe acute respiratory syndrome coronavirus 2 (SARS-CoV-2), remains a significant global concern. As of August 30, 2021, the number of reported cases of COVID-19 in the United States (US) surpassed 37 million, with 634,157 deaths associated with the disease [1].

The majority of people infected with SARS-CoV-2 have mild symptoms [2], but an estimated 20% to 30% experience a severe illness that requires hospitalization [2, 3]. COVID-19 hospitalizations are associated with substantial patient morbidity and mortality and considerable healthcare resource use and costs. A comparative evaluation of the clinical manifestations and risk of death in patients admitted to hospital with COVID-19 or seasonal influenza in the US between February 1, 2020 and June 17, 2020 showed a higher risk of extrapulmonary organ dysfunction, death, and increased healthcare resource use, including intensive care unit (ICU) admission and invasive mechanical ventilation (IMV), among patients hospitalized with COVID-19 [4].

The management and treatment of patients hospitalized with COVID-19 have required healthcare providers to navigate unexplored territory [5]. Several pharmacological treatments have been re-purposed in the inpatient setting throughout the pandemic based on rapidly evolving clinical and real-world evidence, regulatory decisions, federal guidelines, and practice patterns [6, 7]. Randomized controlled trials have generated data on the efficacy of remdesivir for patients hospitalized with COVID-19 [8–10]. In May 2020, remdesivir was granted Emergency Use Authorization (EUA) for the treatment of patients hospitalized with severe COVID-19 [11], and in October 2020, remdesivir was the first, and remains the only, Food and Drug Administration (FDA)-approved treatment for COVID-19 requiring hospitalization [12]. The use of dexamethasone for hospitalized patients emerged in June 2020 based on evidence from the RECOVERY trial [13]. Other therapeutics, including azithromycin [14] and tocilizumab [15–17], are being used as off-label treatments for COVID-19 in the inpatient setting based on preliminary efficacy data from clinical trials or effectiveness data from real-world observational studies.

Current clinical management of COVID-19 consists of supportive care, including supplemental oxygen and mechanical ventilation when indicated [18]. Since its EUA, remdesivir has been recommended by the National Institute of Health (NIH) COVID-19 treatment guidelines for the treatment of COVID-19 in hospitalized patients with severe disease, requiring supplemental oxygen, mechanical ventilation, or extracorporeal membrane oxygenation (ECMO). A combination of dexamethasone and remdesivir, although not studied in clinical trials, is recommended for patients who require increasing amounts of supplemental oxygen, and those requiring non-invasive ventilation [18]. Dexamethasone may be used alone when combination therapy with remdesivir cannot be used or is unavailable, and is recommended for patients who require supplemental oxygen or mechanical ventilation, as well as for patients receiving IMV or ECMO. Tocilizumab may be added to the treatment regimen for patients with rapidly increasing oxygen needs in the presence of systemic inflammation and receiving non-invasive or invasive ventilation. Although guidelines for patients hospitalized with COVID-19 are being updated to provide optimal treatment recommendations [18], significant questions about best treatment practices remain.

Evidence-based clinical data on COVID-19 pharmacotherapies are scarce, and it remains unclear what determines the use of specific treatments in patients hospitalized with COVID-19. In a cohort of 2,821 US patients hospitalized with COVID-19 between March 1, 2020 and May 24, 2020, pharmacological treatment was largely driven by disease severity. Over the study period, use of COVID-19 specific medications and other supportive medications decreased, while use of remdesivir and therapeutic anticoagulants increased; however, patient outcomes were not reported [7]. Awareness of the demographics, clinical characteristics, treatment patterns, and outcomes of patients hospitalized with COVID-19 is required to inform evolving standards of care.

The objective of this study was to describe the pre-treatment characteristics, real-world treatment patterns, and clinical and health resource use outcomes among adult patients diagnosed with COVID-19 in the US who initiated commonly used treatment regimens for COVID-19 in the inpatient setting between September 2020 and January 2021. The data period was selected based on the following considerations: 1) improved use of International Classification of Diseases, 10th Revision, Clinical Modification (ICD-10-CM) diagnosis codes to identify confirmed COVID-19 cases; and 2) treatments used in the inpatient setting were reflective of recent practice guidelines following the release of the revoked EUA of hydroxychloroquine [19], expanded EUA of remdesivir [20], and EUA of convalescent plasma [21]. The findings of this large nationwide study spanning a recent period of the COVID-19 pandemic will advance our understanding of the drivers of treatment among patients hospitalized with COVID-19 in the US and serve as a basis for ongoing and future prospective studies.

## Methods

**Fig 1:**
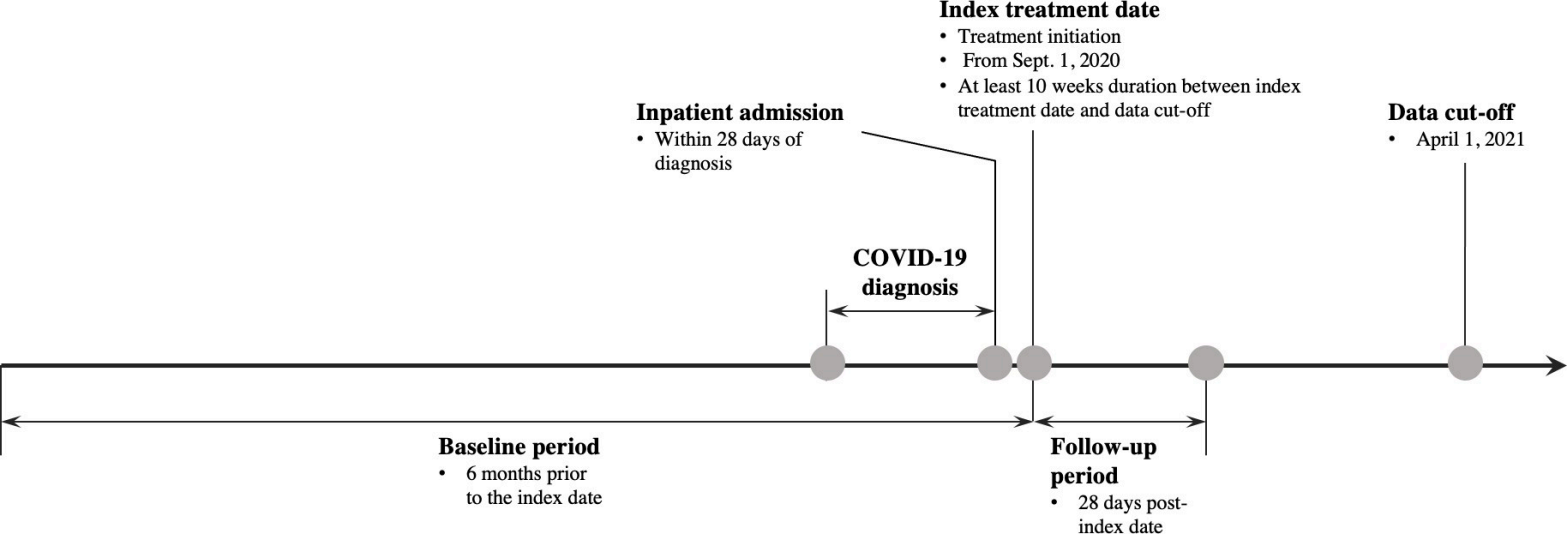
Study design. COVID-19, coronavirus disease 2019. Note: The duration between index treatment date and data cut-off date was at least 10 weeks to allow for full reporting of the deaths that occurred among the patients during the 28 days of follow-up.

This retrospective cohort study examined patient characteristics, treatment patterns, and clinical outcomes among hospitalized adult patients diagnosed with COVID-19 who received treatments of interest for COVID-19 on or after September 1, 2020 (**Fig 1**).

### Data source

This study used data extracted from the de-identified Optum^®^ COVID-19 electronic health records (EHR) database [22]. The data are certified as de-identified by an independent statistical expert following HIPAA statistical de-identification rules and managed according to Optum^®^ customer data use agreements [23, 24]. The COVID-19 EHR database includes patients in the Optum^®^ EHR database who have 1) a documented diagnosis of COVID-19 or acute respiratory illness after February 1, 2020 according to the following codes: U07.1, B97.29 and J12.89 or J20.8, or J40, or J22, or J98.8, or J80; and/or 2) documented COVID-19 antigen or nucleic acid amplification testing (positive or negative result). The database incorporates raw clinical data from inpatient and ambulatory electronic medical records (EMRs), practice management systems, and other internal systems.

### Study population

Adult patients hospitalized with a COVID-19 diagnosis who received treatments of interest for COVID-19 were included in this study. The COVID-19 diagnosis date was defined as the first occurrence of any of the following events: 1) a positive SARS-CoV-2 viral RNA test; 2) a positive antigen test for SARS-CoV-2; or 3) ICD-10-CM diagnosis code of U07.1. Additional sample selection criteria were: 1) ≥18 years of age; 2) admission to an inpatient facility 0 to 28 days (inclusive) after the COVID-19 diagnosis date; 3) treatment with any of the 12 treatments of interest; and 4) index treatment date on or after September 1, 2020 and at least 10 weeks prior to the data cut-off (April 1, 2021) to allow a follow-up duration of 28 days (up to February, 2021) and an additional 6 weeks for full reporting of mortality. Exclusion criteria were: 1) missing age or gender; 2) labor or delivery codes during the index admission (**S1 Table**); or 3) trauma, injury, fracture, or poisoning diagnosis codes (presence of ≥1 ICD-10-CM code: S00-S99, T07-T73) on the day of or on the next day after the index admission date.

**Fig 2:**
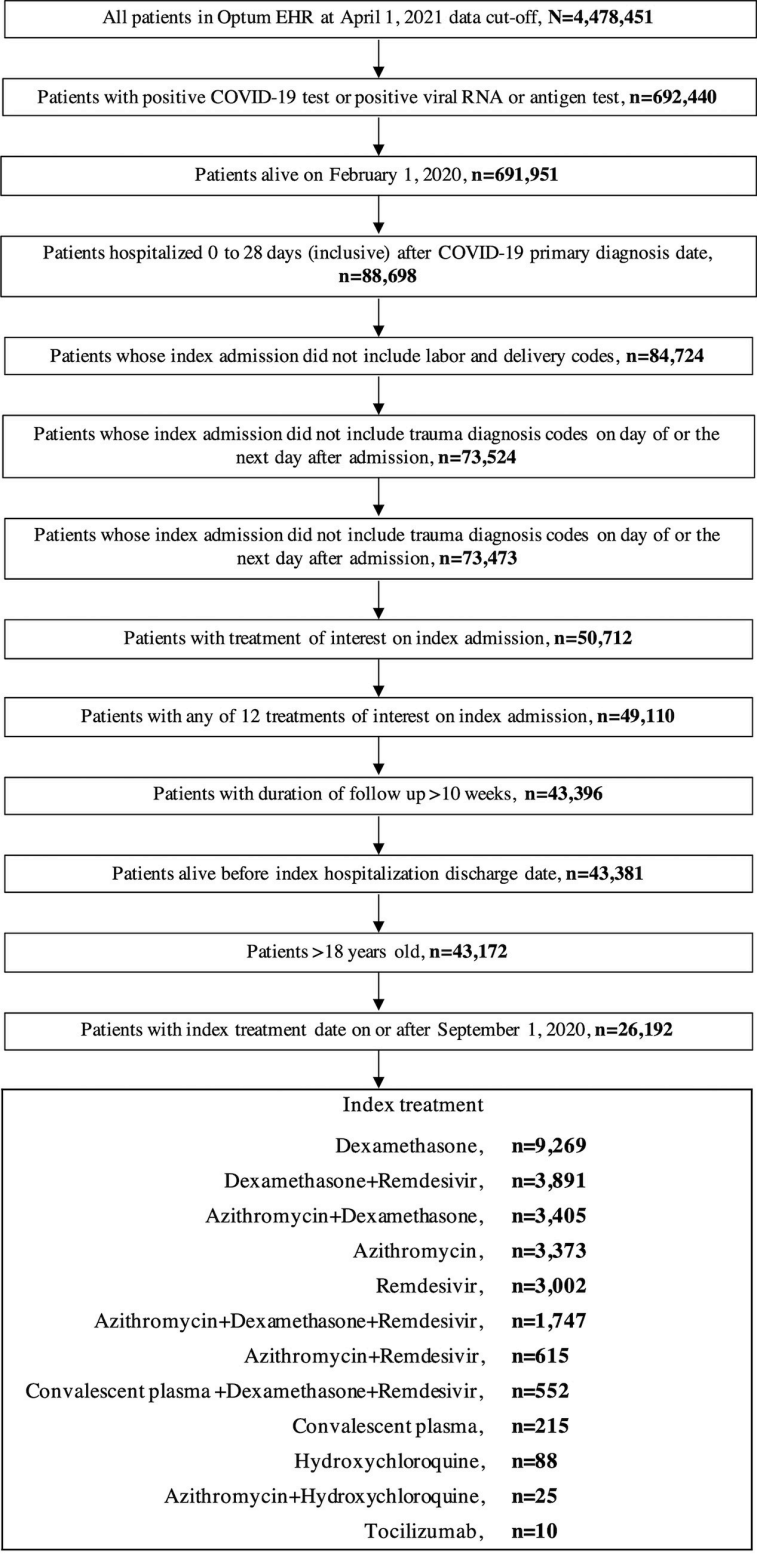
Sample selection. The first inpatient admission after COVID-19 diagnosis was defined as the index admission. Index treatment was defined as the earliest regimen of interest received during the index admission, with the date of initiation serving as the index treatment date.

Treatments of interest for COVID-19 comprised the 12 most frequently used treatments and combination regimens, including dexamethasone, dexamethasone + remdesivir, azithromycin + dexamethasone, azithromycin, remdesivir, azithromycin + dexamethasone + remdesivir, azithromycin + remdesivir, convalescent plasma + dexamethasone + remdesivir, convalescent plasma alone, hydroxychloroquine, azithromycin + hydroxychloroquine, and tocilizumab (**Fig 2**). Combination therapy was defined as receipt of ≥2 treatments of interest on the same day.

Index admission was defined as the first inpatient admission after a COVID-19 diagnosis documented in the EHR. Index treatment was defined as the earliest treatment of interest for COVID-19 received during the index admission, with the date of treatment initiation serving as the index treatment date. Post-index treatment was defined as any treatment of interest for COVID-19, or any combination thereof, received after the index treatment during the 28-day follow-up period. The baseline period was defined as the 6 months prior to the index treatment date. The follow-up period was defined as the 28 days following the index treatment date. The duration between index treatment date and data cut-off date (April 1, 2021) was at least 10 weeks to allow for full follow-up in death reporting accounting for a potential lag in report of mortality that occurred among the patients during the 28 days of follow-up.

### Study measures

Patient pre-treatment characteristics (age, sex, race, geographic region, body mass index [BMI]), care setting (e.g. ICU), and medical procedures and supportive care (oxygen supplementation, non-invasive ventilation, invasive ventilation, ECMO, vasopressor use) were recorded at index admission or on the index treatment date. Medical history (cardiac disease, respiratory disease, obesity, diabetes mellitus, kidney disease, venous thromboembolism) based on ICD-10-CM diagnosis and procedure codes were assessed during the baseline period (**S2 Table**).

Index and post-index treatment use were identified based on National Drug Code or procedure codes for drug administration when applicable. Index treatment was recorded at the index treatment date. Post-index treatment was recorded on Days 7, 14, 21, and 28.

Patient clinical status was assessed using a 4-point ordinal scale (0: discharged alive from the index admission; 1: hospitalized without oxygen/ventilation use; 2: hospitalized with oxygen/ventilation use; and 3: recorded death including post-discharge death). Patient clinical status was recorded on the index treatment date and at Days 7, 14, 21, and 28 post-index.

Patient disposition (in hospital, discharged alive, death during index admission, and death after discharge from index admission), clinical improvement (defined as improvement of ≥1 point in clinical status from the index treatment date), and clinical outcomes (cumulative inpatient days, cumulative days of ICU care, and cumulative days with mechanical ventilation or ECMO) were recorded during the follow-up period. The occurrence of death or organ failure (defined as occurrence of heart failure, pulmonary system failure, renal failure, or hepatic failure) was assessed as a binary indicator of presence/absence at index admission and during the follow-up period.

### Data analysis

Descriptive analyses were performed for the overall population and each cohort stratified by index treatment. Patient demographic and clinical characteristics, patient disposition, clinical improvement, and clinical outcomes were summarized descriptively. Post-index treatment patterns and patient clinical status were summarized and depicted using Sankey diagrams. Maps depicting geographic distribution of index treatments were generated using US Census regions and divisions of the US. Results are descriptive, and all comparisons are qualitative in nature.

## Results

### Pre-treatment patient characteristics

A total of 26,192 patients met the eligibility criteria for this study (**Fig 2**). The descriptive analysis showed 9,269 (35.4%) patients received dexamethasone as the index treatment, 3,891(14.9%) patients received dexamethasone + remdesivir, 3,405 (13.0%) patients received azithromycin + dexamethasone, 3,373 (12.9%) patients received azithromycin, 3,002 (11.5%) patients received remdesivir, and 1,747 (6.7%) patients received azithromycin + dexamethasone + remdesivir. The remaining treatment cohorts each included <5% of all patients. Among these, 615 (2.3%) patients received azithromycin + remdesivir, 552 (2.1%) patients received convalescent plasma + dexamethasone + remdesivir, 215 (0.8%) patients received convalescent plasma, 88 (0.3%) patients received hydroxychloroquine, 25 (0.1%) patients received azithromycin + hydroxychloroquine, and 10 (<0.1%) patients received tocilizumab. Index treatment use over time from September 2020 to January 2021 (inclusive) is summarized for each treatment of interest in **S1 Fig**. Treatment patterns were generally reflective of practice guidelines during the study period (after September 2020) [18].

In the overall population, mean (standard deviation [SD]) time between index admission and index treatment date was 0.6 (1.7) days. Mean time between index admission and index treatment date across treatment cohorts was similar to or shorter than the overall population, with the exception of the remdesivir (1.0 [1.6] days post-admission), convalescent plasma (1.5 [3.2] days post-admission), and tocilizumab (3.8 [2.8] days post-admission) cohorts.

Patient demographics and medical history are summarized in **Table 1**. In the overall population, mean (SD) patient age was 65.6 (15.6) years, which was similar across treatment cohorts. Exceptions included the azithromycin + remdesivir (mean [SD] 64.2 [15.8] years), hydroxychloroquine (61.9 [16.4] years), and azithromycin + hydroxychloroquine (60.8 [16.1] years) cohorts. In the overall population, more than half of the patients were male (53.6%). The sex distribution within treatment cohorts was similar to that of the overall population, with the exception of patients in the hydroxychloroquine, and azithromycin + hydroxychloroquine cohorts, where there were a smaller proportion of males (15.9% and 32.0%, respectively).

**Table 1:** Pre-treatment patient characteristics.

	**Overall (N = 26,192)**	**Dexamethasone (N = 9,269)**	**Dexamethasone +Remdesivir (N = 3,891)**	**Azithromycin +Dexamethasone (N = 3,405)**	**Azithromycin (N = 3,373)**	**Remdesivir (N = 3,002)**	**Azithromycin +Dexamethasone +Remdesivir (N = 1,747)**
**Age at index treatment date (years)**							
Mean (SD)	65.6 (15.6)	65.2 (16.0)	66.0 (15.1)	66.5 (15.3)	64.7 (16.6)	66.9 (14.5)	65.6 (14.8)
18–64, n (%)	11,348 (43.3)	4,105 (44.3)	1,702 (43.7)	1,402 (41.2)	1,516 (44.9)	1,204 (40.1)	774 (44.3)
≥65, n (%)	14,844 (56.7)	5,164 (55.7)	2,189 (56.3)	2,003 (58.8)	1,857 (55.1)	1,798 (59.9)	973 (55.7)
**Male, n (%)**	14,044 (53.6)	4,751 (51.3)	2,141 (55.0)	1,917 (56.3)	1,773 (52.6)	1,624 (54.1)	1,035 (59.2)
**Race, n (%)**							
White	18,657 (71.2)	6,917 (74.6)	2,920 (75.0)	2,566 (75.4)	1,900 (56.3)	2,007 (66.9)	1,358 (77.7)
Black	3,381 (12.9)	1,007 (10.9)	325 (8.4)	377 (11.1)	713 (21.1)	562 (18.7)	151 (8.6)
Asian	531 (2.0)	176 (1.9)	86 (2.2)	57 (1.7)	81 (2.4)	54 (1.8)	35 (2.0)
Other/Unknown	3,623 (13.8)	1,169 (12.6)	560 (14.4)	405 (11.9)	679 (20.1)	379 (12.6)	203 (11.6)
**Geographic region, n (%)**							
East North Central	6,282 (24.0)	2,320 (25.0)	725 (18.6)	739 (21.7)	845 (25.1)	963 (32.1)	308 (17.6)
South Atlantic/ West South Central	4,793 (18.3)	1,726 (18.6)	745 (19.1)	653 (19.2)	248 (7.4)	859 (28.6)	328 (18.8)
West North Central	4,699 (17.9)	1,850 (20.0)	785 (20.2)	697 (20.5)	450 (13.3)	114 (3.8)	433 (24.8)
Middle Atlantic	3,808 (14.5)	1,004 (10.8)	586 (15.1)	319 (9.4)	1,244 (36.9)	162 (5.4)	165 (9.4)
East South Central	2,395 (9.1)	729 (7.9)	170 (4.4)	453 (13.3)	152 (4.5)	633 (21.1)	195 (11.2)
Mountain	1,759 (6.7)	810 (8.7)	223 (5.7)	344 (10.1)	161 (4.8)	45 (1.5)	134 (7.7)
New England	1,322 (5.0)	464 (5.0)	472 (12.1)	78 (2.3)	116 (3.4)	65 (2.2)	105 (6.0)
Pacific	412 (1.6)	106 (1.1)	75 (1.9)	33 (1.0)	77 (2.3)	76 (2.5)	18 (1.0)
Other/Unknown	722 (2.8)	260 (2.8)	110 (2.8)	89 (2.6)	80 (2.4)	85 (2.8)	61 (3.5)
**BMI category, n (%)**							
Underweight	313 (1.2)	109 (1.2)	33 (0.8)	40 (1.2)	62 (1.8)	35 (1.2)	15 (0.9)
Healthy weight	2,748 (10.5)	1,086 (11.7)	360 (9.3)	309 (9.1)	369 (10.9)	344 (11.5)	122 (7.0)
Overweight	4,524 (17.3)	1,716 (18.5)	645 (16.6)	515 (15.1)	568 (16.8)	575 (19.2)	264 (15.1)
Obese	3,982 (15.2)	1,391 (15.0)	601 (15.4)	482 (14.2)	484 (14.3)	520 (17.3)	236 (13.5)
Morbidly obese	5,296 (20.2)	1,919 (20.7)	773 (19.9)	646 (19.0)	539 (16.0)	710 (23.7)	364 (20.8)
Unknown	9,329 (35.6)	3,048 (32.9)	1,479 (38.0)	1,413 (41.5)	1,351 (40.1)	818 (27.2)	746 (42.7)
**Medical history, n (%)**							
Cardiac disease							
Hypertension	11,740 (44.8)	4,325 (46.7)	1,806 (46.4)	1,409 (41.4)	1,356 (40.2)	1,460 (48.6)	695 (39.8)
Arrhythmia	4,309 (16.5)	1,662 (17.9)	697 (17.9)	461 (13.5)	482 (14.3)	548 (18.3)	232 (13.3)
Coronary artery disease	3,762 (14.4)	1,384 (14.9)	556 (14.3)	453 (13.3)	438 (13.0)	513 (17.1)	202 (11.6)
Heart failure	3,121 (11.9)	1,160 (12.5)	471 (12.1)	354 (10.4)	366 (10.9)	414 (13.8)	155 (8.9)
Stroke or TIA	2,033 (7.8)	822 (8.9)	283 (7.3)	212 (6.2)	247 (7.3)	265 (8.8)	90 (5.2)
Respiratory disease							
ARDS/respiratory failure	3,637 (13.9)	1,225 (13.2)	704 (18.1)	301 (8.8)	252 (7.5)	695 (23.2)	217 (12.4)
COPD	3,142 (12.0)	1,071 (11.6)	454 (11.7)	411 (12.1)	379 (11.2)	407 (13.6)	214 (12.2)
Asthma	1,825 (7.0)	708 (7.6)	265 (6.8)	219 (6.4)	214 (6.3)	193 (6.4)	110 (6.3)
Diabetes mellitus	6,729 (25.7)	2,387 (25.8)	1,030 (26.5)	808 (23.7)	768 (22.8)	922 (30.7)	392 (22.4)
Obesity (BMI ≥ 30kg/m^2^)	9,278 (35.4)	3,310 (35.7)	1,374 (35.3)	1,128 (33.1)	1,023 (30.3)	1,230 (41.0)	600 (34.3)
Kidney disease							
Chronic kidney disease	3,904 (14.9)	1,509 (16.3)	539 (13.9)	473 (13.9)	494 (14.6)	422 (14.1)	203 (11.6)
Acute renal failure	3,396 (13.0)	1,378 (14.9)	471 (12.1)	334 (9.8)	387 (11.5)	483 (16.1)	143 (8.2)
VTE	836 (3.2)	295 (3.2)	126 (3.2)	82 (2.4)	113 (3.4)	122 (4.1)	45 (2.6)

	**Azithromycin +Remdesivir (N = 615)**	**Convalescent Plasma +Dexamethasone +Remdesivir (N = 552)**	**Convalescent Plasma (N = 215)**	**Hydroxychloroquine (N = 88)**	**Azithromycin +Hydroxychloroquine (N = 25)**
**Age at index treatment date (years)**					
Mean (SD)	64.2 (15.8)	66.2 (14.1)	68.3 (15.1)	61.9 (16.4)	60.8 (16.1)
18–64, n (%)	284 (46.2)	229 (41.5)	72 (33.5)	42 (47.7)	14 (56.0)
≥65, n (%)	331 (53.8)	323 (58.5)	143 (66.5)	46 (52.3)	11 (44.0)
**Male, n (%)**	351 (57.1)	305 (55.3)	120 (55.8)	14 (15.9)	8 (32.0)
**Race, n (%)**					
White	307 (49.9)	436 (79.0)	164 (76.3)	61 (69.3)	13 (52.0)
Black	155 (25.2)	41 (7.4)	32 (14.9)	12 (13.6)	5 (20.0)
Asian	16 (2.6)	17 (3.1)	2 (0.9)	5 (5.7)	2 (8.0)
Other/Unknown	137 (22.3)	58 (10.5)	17 (7.9)	10 (11.4)	5 (20.0)
**Geographic region, n (%)**					
East North Central	167 (27.2)	63 (11.4)	112 (52.1)	27 (30.7)	6 (24.0)
South Atlantic/West South Central	22 (3.6)	167 (30.3)	26 (12.1)	11 (12.5)	6 (24.0)
West North Central	85 (13.8)	220 (39.9)	44 (20.5)	14 (15.9)	7 (28.0)
Middle Atlantic	280 (45.5)	27 (4.9)	1 (0.5)	15 (17.0)	5 (20.0)
East South Central	6 (1.0)	31 (5.6)	22 (10.2)	4 (4.5)	0
Mountain	7 (1.1)	24 (4.3)	4 (1.9)	7 (8.0)	0
New England	11 (1.8)	4 (0.7)	0	6 (6.8)	1 (4.0)
Pacific	20 (3.3)	3 (0.5)	1 (0.5)	3 (3.4)	0
Other/Unknown	17 (2.8)	13 (2.4)	5 (2.3)	1 (1.1)	0
**BMI category, n (%)**					
Underweight	7 (1.1)	8 (1.4)	3 (1.4)	1 (1.1)	0
Healthy weight	63 (10.2)	42 (7.6)	32 (14.9)	17 (19.3)	3 (12.0)
Overweight	107 (17.4)	75 (13.6)	41 (19.1)	11 (12.5)	5 (20.0)
Obese	119 (19.3)	88 (15.9)	39 (18.1)	14 (15.9)	5 (20.0)
Morbidly obese	136 (22.1)	140 (25.4)	42 (19.5)	19 (21.6)	7 (28.0)
Unknown	183 (29.8)	199 (36.1)	58 (27.0)	26 (29.5)	5 (20.0)
**Medical history, n (%)**					
Cardiac disease					
Hypertension	253 (41.1)	256 (46.4)	131 (60.9)	34 (38.6)	11 (44.0)
Arrhythmia	78 (12.7)	87 (15.8)	43 (20.0)	13 (14.8)	4 (16.0)
Coronary artery disease	82 (13.3)	75 (13.6)	44 (20.5)	10 (11.4)	3 (12.0)
Heart failure	73 (11.9)	65 (11.8)	44 (20.5)	15 (17.0)	4 (16.0)
Stroke or TIA	56 (9.1)	28 (5.1)	21 (9.8)	6 (6.8)	2 (8.0)
Respiratory disease					
ARDS/respiratory failure	55 (8.9)	100 (18.1)	75 (34.9)	7 (8.0)	0
COPD	78 (12.7)	70 (12.7)	43 (20.0)	13 (14.8)	2 (8.0)
Asthma	42 (6.8)	40 (7.2)	21 (9.8)	10 (11.4)	1 (4.0)
Diabetes mellitus	166 (27.0)	148 (26.8)	75 (34.9)	20 (22.7)	10 (40.0)
Obesity (BMI ≥ 30kg/m^2^)	255 (41.5)	228 (41.3)	81 (37.7)	33 (37.5)	12 (48.0)
Kidney disease					
Chronic kidney disease	91 (14.8)	91 (16.5)	61 (28.4)	15 (17.0)	4 (16.0)
Acute renal failure	63 (10.2)	50 (9.1)	67 (31.2)	16 (18.2)	3 (12.0)
VTE	19 (3.1)	15 (2.7)	12 (5.6)	4 (4.5)	1 (4.0)

ARDS: acute respiratory distress syndrome; BMI: body mass index; COPD: chronic obstructive pulmonary disease; SD: standard deviation; TIA: transient ischemic attack; VTE: venous thromboembolism.

Data for the tocilizumab cohort are not presented due to small sample size.

**Fig 3:**
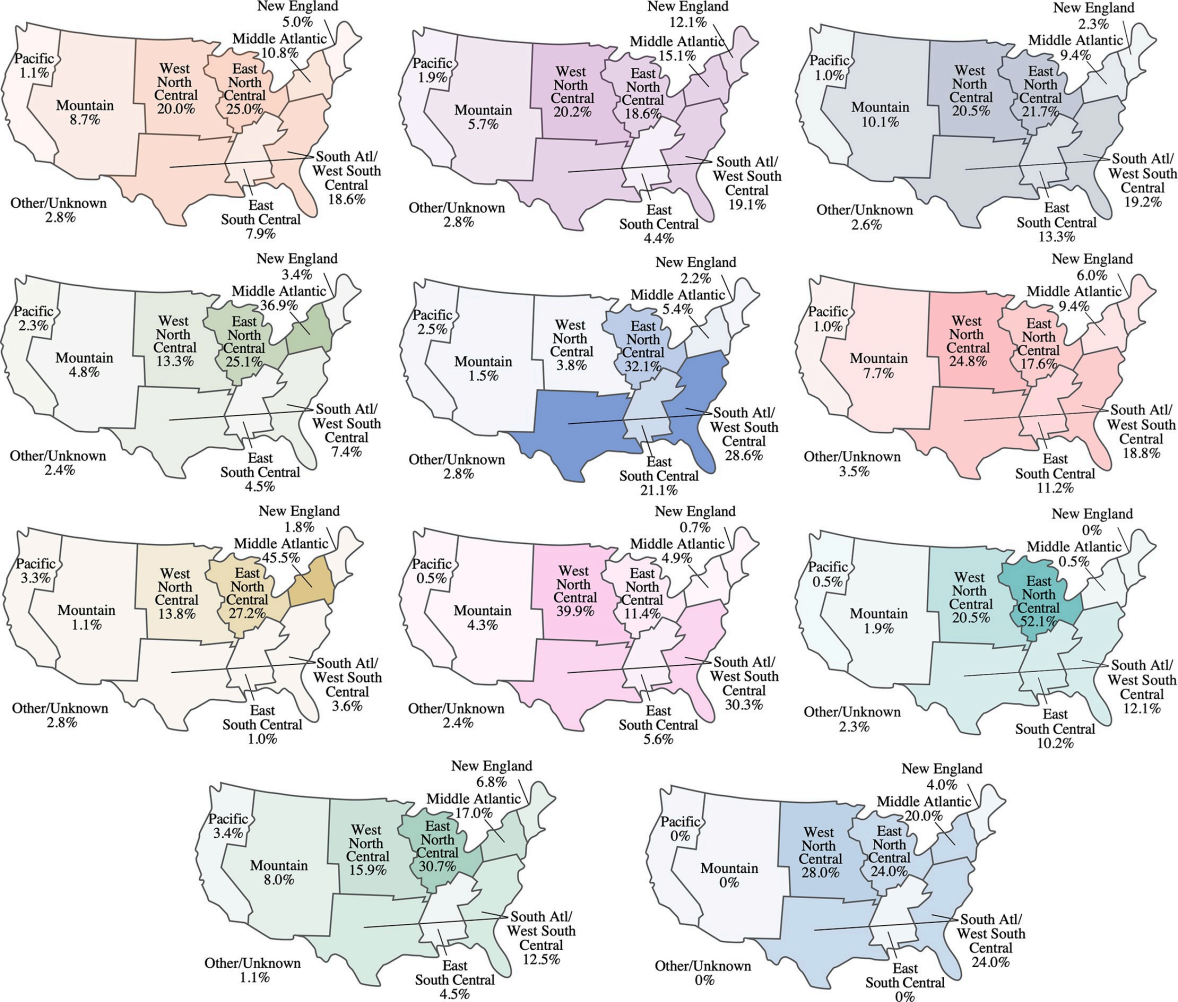
Geographical distribution of index treatment use. a) Dexamethasone (n = 9,269), b) Dexamethasone + remdesivir (n = 3,891), c) Azithromycin + dexamethasone (n = 3,405), d) Azithromycin (n = 3,373), e) Remdesivir (n = 3,002), f) Azithromycin + dexamethasone + remdesivir (n = 1,747), G) Azithromycin + remdesivir (n = 615), h) Convalescent plasma + dexamethasone + remdesivir (n = 552), i) Convalescent plasma (n = 215), j) Hydroxychloroquine (n = 88), k) Azithromycin + hydroxychloroquine (n = 25). * Includes Alaska and Hawaii. Data for the tocilizumab cohort are not presented due to small sample size.

In the overall population, 71.2% of patients were White, 12.9% were Black, and 2.0% were Asian. Racial distribution across treatment cohorts was similar to the overall population. Exceptions included the azithromycin, azithromycin + remdesivir, and azithromycin + hydroxychloroquine cohorts, where a smaller proportion of patients were White (56.3%, 49.9%, and 52.0%, respectively) and a higher proportion of patients were Black (21.1%, 25.2%, and 20.0%, respectively). In the tocilizumab cohort, 80.0% of patients were White and 10.0% of patients were Black. In the overall population, most patients resided in the East North Central (24.0%), South Atlantic/West South Central (18.3%), West North Central (17.9%), and Middle Atlantic (14.5%) regions. Geographic distribution was similar across some treatment cohorts; however, regional differences in index treatment use were observed (**Fig 3**).

In the overall population, 44.8% of patients had hypertension, 35.4% of patients were obese or morbidly obese based on BMI (BMI data were missing for 35.6% of patients), and 25.7% of patients had diabetes mellitus. Other comorbidities prevalent in the overall population were arrhythmia (16.5%), chronic kidney disease (14.9%), coronary artery disease (14.4%), acute respiratory distress syndrome (ARDS)/respiratory failure (13.9%), acute renal failure (13.0%), chronic obstructive pulmonary disease (COPD) (12.0%), and heart failure (11.9%). Patient medical history profiles were similar across some treatment cohorts; however, comorbidity profiles for certain cohorts were more severe. Compared to the overall population, a higher percentage of patients in the remdesivir cohort had ARDS/respiratory failure (23.2%); a higher percentage of patients in the convalescent plasma cohort had hypertension (60.9%), arrhythmia (20.0%), coronary artery disease (20.5%), heart failure (20.5%), ARDS/respiratory failure (34.9%), COPD (20.0%), chronic kidney disease (28.4%), and acute renal failure (31.2%); and a higher percentage of patients in the tocilizumab cohort had arrhythmia (20.0%), coronary artery disease (20.0%), ARDS/respiratory failure (60.0%), asthma (20.0%), and venous thromboembolism (20.0% vs. 3.2% overall).

### Post-index treatment and patient outcomes

**Fig 4:**
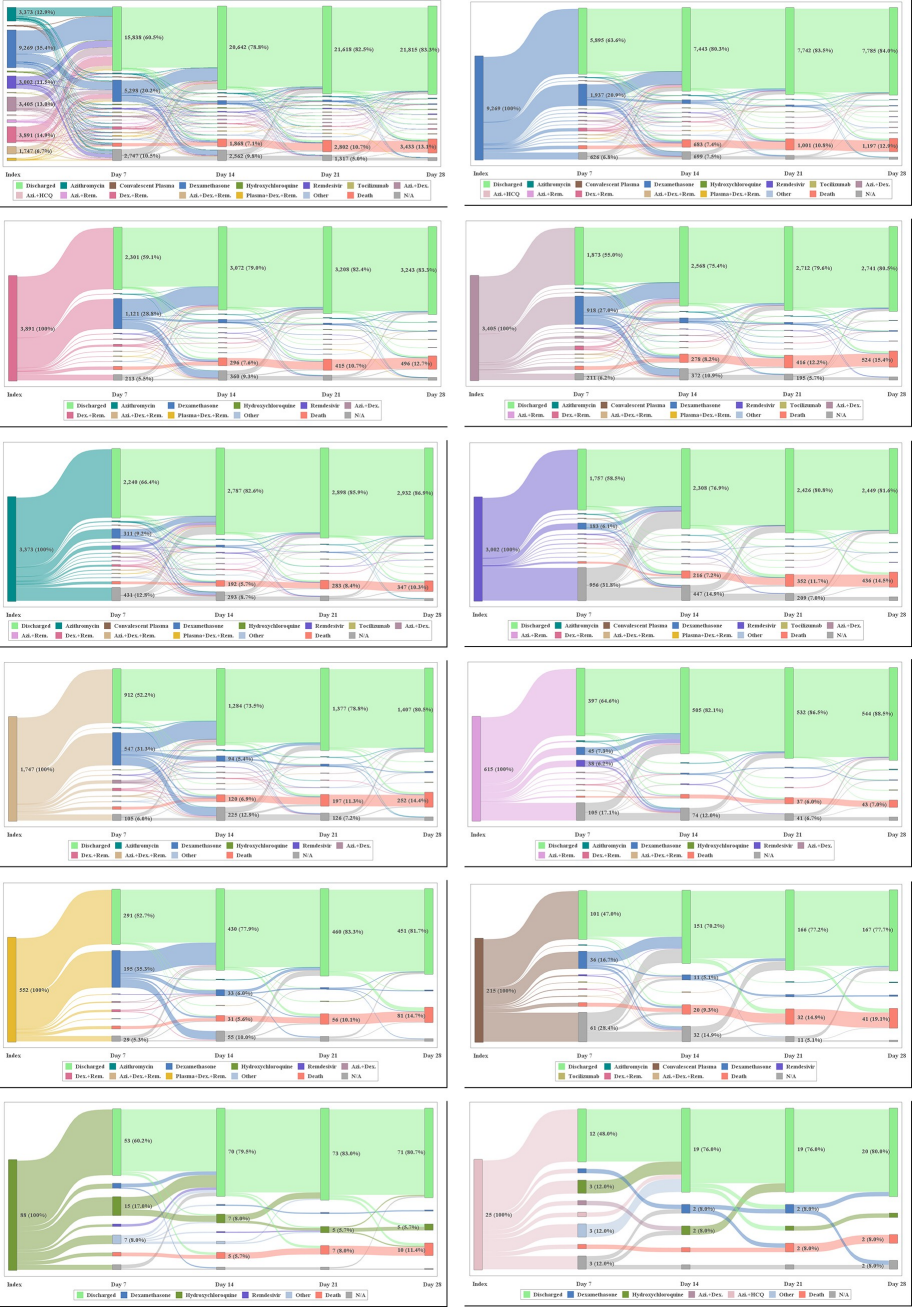
Post-index treatment use. a) Overall, b) Dexamethasone, c) Dexamethasone + Remdesivir, d) Azithromycin + Dexamethasone, e) Azithromycin, f) Remdesivir, g) Azithromycin + Dexamethasone + Remdesivir, h) Azithromycin + Remdesivir, i) Convalescent Plasma + Dexamethasone + Remdesivir, j) Convalescent Plasma, k) Hydroxychloroquine, l) Azithromycin + Hydroxychloroquine. Azi, azithromycin; Dex, dexamethasone; HCQ, hydroxychloroquine; N/A, not available; Rem, remdesivir. Other includes combinations of treatments of interest not assessed in this study. Patient disposition data may not align with Table 2 as treatments are prioritized over death: i.e., if a patient received treatment and died the same day, the patient was categorized under treatment. Data for tocilizumab are not presented due to small sample size.

Treatment and patient disposition at days 7, 14, 21, and 28 post-index are shown in **Fig 4**. At day 7 post-index, dexamethasone was the most prevalent treatment in the overall population (20.2%) and across treatment cohorts, with the exception of the hydroxychloroquine, and azithromycin + hydroxychloroquine cohorts, where hydroxychloroquine was most often administered (17.0% and 12.0%, respectively). At day 14 post-index, across all cohorts, fewer than 10% of patients received any treatments of interest, with the majority of patients discharged.

**Table 2:** Patient disposition and resource use.

	Overall (N = 26,192)	Dexamethasone (N = 9,269)	Dexamethasone +Remdesivir (N = 3,891)	Azithromycin +Dexamethasone (N = 3,405)	Azithromycin (N = 3,373)	Remdesivir (N = 3,002)
**Care setting and medical procedures on the index treatment date, n (%)**						
ICU	2,185 (8.3)	720 (7.8)	360 (9.3)	352 (10.3)	163 (4.8)	189 (6.3)
Oxygen supplementation	1,171 (4.5)	374 (4.0)	253 (6.5)	182 (5.3)	100 (3.0)	82 (2.7)
Non-invasive ventilation	775 (3.0)	246 (2.7)	126 (3.2)	146 (4.3)	59 (1.7)	41 (1.4)
Invasive ventilation	825 (3.1)	303 (3.3)	145 (3.7)	134 (3.9)	52 (1.5)	45 (1.5)
ECMO	6 (< 0.1)	4 (< 0.1)	1 (< 0.1)	0	0	0
Vasopressor use	156 (0.6)	72 (0.8)	25 (0.6)	23 (0.7)	12 (0.4)	6 (0.2)
**Patient disposition at the end of follow-up, n (%)**						
In hospital	803 (3.1)	237 (2.6)	122 (3.1)	123 (3.6)	81 (2.4)	110 (3.7)
Discharged alive	21,955 (83.8)	7,835 (84.5)	3,273 (84.1)	2,758 (81.0)	2,945 (87.3)	2,455 (81.8)
Death during index admission	1,045 (4.0)	399 (4.3)	174 (4.5)	157 (4.6)	107 (3.2)	75 (2.5)
Death after discharge from index admission	2,389 (9.1)	798 (8.6)	322 (8.3)	367 (10.8)	240 (7.1)	362 (12.1)
**Clinical outcomes at the end of the follow-up, mean (SD)**						
Cumulative inpatient days during index admission	8.1 (6.6)	7.2 (6.4)	8.3 (6.4)	8.6 (6.9)	7.5 (6.3)	9.2 (6.7)
Cumulative days with ICU care during index admission	1.4 (4.1)	1.2 (3.7)	1.5 (4.3)	1.7 (4.4)	0.8 (3.0)	1.4 (4.3)
Cumulative days with mechanical ventilation or ECMO during index admission	0.9 (3.1)	0.8 (2.9)	1.0 (3.1)	1.2 (3.6)	0.6 (2.3)	0.6 (2.5)
	**Azithromycin +Dexamethasone +Remdesivir (N = 1,747)**	**Azithromycin +Remdesivir (N = 615)**	**Convalescent Plasma +Dexamethasone +Remdesivir (N = 552)**	**Convalescent Plasma (N = 215)**	**Hydroxychloroquine (N = 88)**	**Azithromycin +Hydroxychloroquine (N = 25)**
**Care setting and medical procedures on the index treatment date, n (%)**						
ICU	243 (13.9)	46 (7.5)	72 (13.0)	33 (15.3)	5 (5.7)	1 (4.0)
Oxygen supplementation	122 (7.0)	21 (3.4)	30 (5.4)	5 (2.3)	2 (2.3)	0
Non-invasive ventilation	92 (5.3)	15 (2.4)	40 (7.2)	10 (4.7)	0	0
Invasive ventilation	92 (5.3)	16 (2.6)	26 (4.7)	10 (4.7)	0	1 (4.0)
ECMO	1 (< 0.1)	0	0	0	0	0
Vasopressor use	14 (0.8)	3 (0.5)	1 (0.2)	0	0	0
**Patient disposition at the end of follow-up, n (%)**						
In hospital	78 (4.5)	23 (3.7)	18 (3.3)	6 (2.8)	2 (2.3)	2 (8.0)
Discharged alive	1,417 (81.1)	549 (89.3)	453 (82.1)	168 (78.1)	76 (86.4)	21 (84.0)
Death during index admission	83 (4.8)	14 (2.3)	24 (4.3)	10 (4.7)	1 (1.1)	0
Death after discharge from index admission	169 (9.7)	29 (4.7)	57 (10.3)	31 (14.4)	9 (10.2)	2 (8.0)
**Clinical outcomes at the end of the follow-up, mean (SD)**						
Cumulative inpatient days during index admission	9.6 (7. 2)	8.7 (6.7)	9.1 (6.4)	10.1 (7.0)	7.4 (6.5)	9.0 (7.5)
Cumulative days of ICU care during index admission	2.4 (5.4)	1.4 (4.3)	1.7 (4.2)	1.8 (3.8)	0.8 (2.8)	1.1 (4.0)
Cumulative days of mechanical ventilation or ECMO during index admission	1.6 (4.2)	0.8 (3.0)	1.2 (3.2)	1.3 (3.2)	0.5 (2.7)	0.4 (1.6)

ECMO, extracorporeal membrane oxygenation; ICU, intensive care unit; SD, standard deviation.

Patient disposition data may not align with Fig 3 as death is prioritized over other categories.

Data for the tocilizumab cohort are not presented due to small sample size.

Post-index treatment switching in the overall population, regardless of the time of switch, for up to 4 treatments including index treatment, is shown in **S2 Fig**. Dexamethasone was the most common second (17.8%), third (15.3%) and fourth (8.4%) treatment, followed by dexamethasone + remdesivir (second treatment 16.9%, third treatment 8.2%).

### Patient disposition and resource use

Medical resource use at the index treatment date and patient disposition at the end of follow-up are summarized in **Table 2**. In the overall population, at day 28 post-index, patients had mean (SD) 8.1 (6.6) cumulative inpatient days, which was similar across treatment cohorts. At the index treatment date, 8.3% of patients were in ICU care, which was similar across treatment cohorts. Exceptions included the convalescent plasma (15.3%), azithromycin + dexamethasone + remdesivir (13.9%), and convalescent plasma + dexamethasone + remdesivir (13.0%) cohorts. At day 28 post-index, mean (SD) cumulative days with ICU care was 1.4 (4.1) for patients in the overall population, which was similar across treatment cohorts. Exceptions included the azithromycin + dexamethasone + remdesivir and tocilizumab cohorts, where patients had a mean (SD) 2.4 and 3.7 (9.0) cumulative days in ICU care, respectively.

In the overall population, oxygen supplementation at the index treatment date was required by 4.5% of patients, and non-invasive or invasive ventilation was required by 3.0% and 3.1% of patients, respectively. Use of ECMO (<0.1%) and vasopressor (0.6%) was low. Some differences in the distribution of these medical procedures were observed across treatment cohorts; however, the proportions were small. At day 28 post-index, patients in the overall population and across treatment cohorts had approximately 1 day of mechanical ventilation or ECMO, with the exception of the tocilizumab cohort, where patients required mean (SD) 3.5 (8.7) days of ventilation or ECMO.

At day 28 post-index, 3.1% of patients in the overall population remained in hospital, 83.8% were discharged alive, 4.0% died in hospital, and 9.1% died after being discharged alive. The distribution of these outcomes was similar across treatment cohorts, with the exception of the tocilizumab cohort, where 10.0% of patients remained hospitalized, 50.0% were discharged alive, 10.0% died in hospital, and 30.0% died after discharge.

### Clinical status

**Fig 5:**
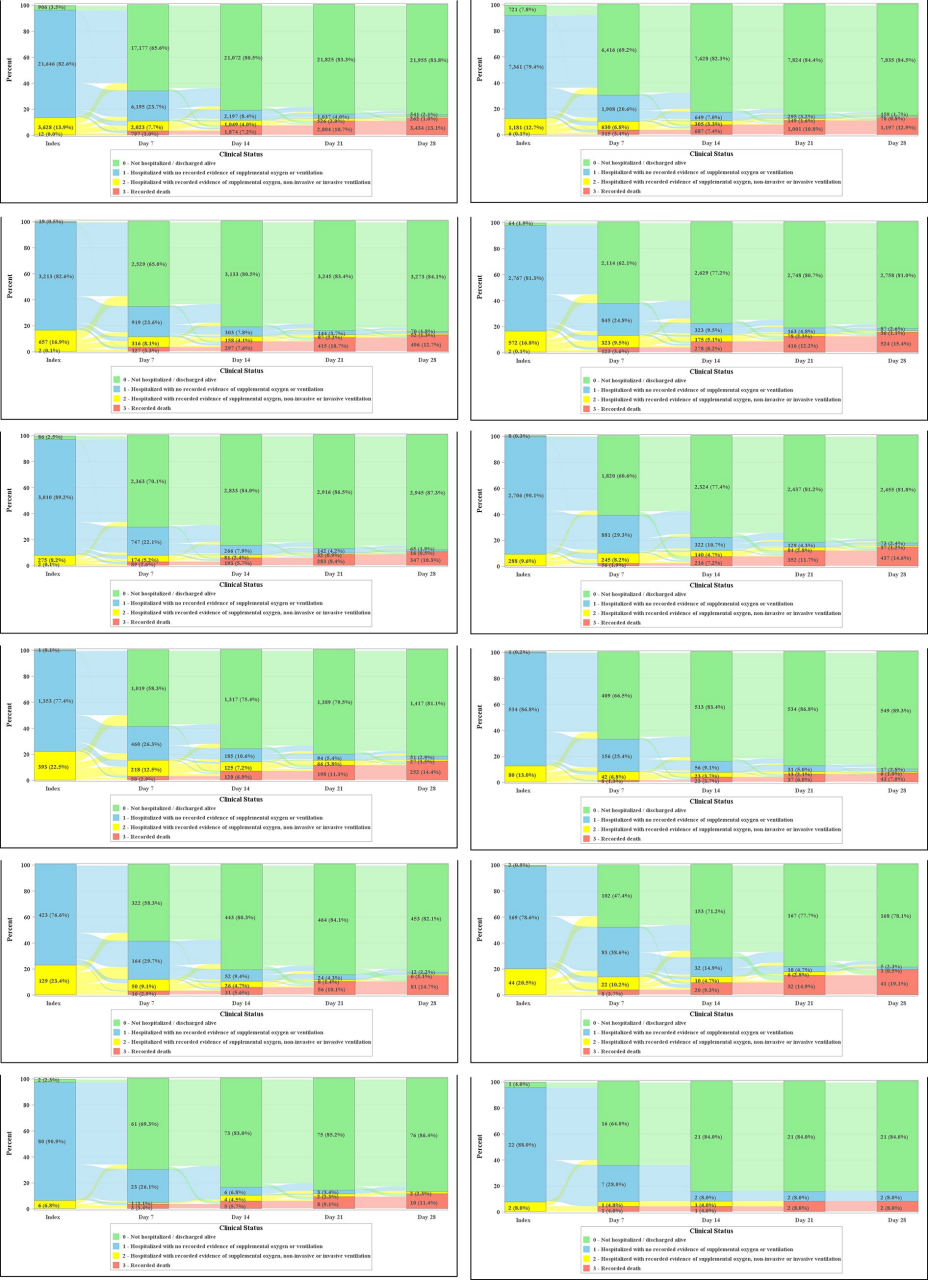
Post-index clinical status. a) Overall, b) Dexamethasone, c) Dexamethasone + Remdesivir, d) Azithromycin + Dexamethasone, e) Azithromycin, f) Remdesivir, g) Azithromycin + Dexamethasone + Remdesivir, h) Azithromycin + Remdesivir, i) Convalescent Plasma + Dexamethasone + Remdesivir, j) Convalescent Plasma, k) Hydroxychloroquine, l) Azithromycin + Hydroxychloroquine. Data for tocilizumab are not presented due to small sample size.

Clinical status of patients at the index treatment date and days 7, 14, 21 and 28 post-index assessed using the 4-point ordinal scale is shown in **Fig 5**. In the overall population, at the index treatment date, 96.5% of patients remained hospitalized, 13.9% of patients were hospitalized with recorded evidence of supplemental oxygen, 3.5% of patients were discharged, and <0.1% of patients died. The distribution of patient clinical status at the index treatment date was similar across most treatment cohorts. Exceptions included the convalescent plasma + dexamethasone + remdesivir, azithromycin + dexamethasone + remdesivir, and convalescent plasma cohorts, where 23.4%, 22.5%, and 20.5% of patients, respectively, had recorded evidence of supplemental oxygen or ventilation.

At day 7 post-index, 65.6% of patients in the overall population were discharged alive, 31.4% of patients remained hospitalized (7.7% and 23.7% of patients were with and without recorded evidence of supplemental oxygen, respectively), and 3.0% of patients had died. The distribution of patient clinical status was similar across treatment cohorts, with the exception of the convalescent plasma cohort, where 47.4% of patients were discharged alive, 48.8% of patients remained hospitalized (10.2% and 38.6% of patients were with and without supplemental oxygen, respectively), and 3.7% of patients died.

At day 28 post-index, 84.2% of patients in the overall population had evidence of clinical improvement. The distribution of clinical status at day 28 post-index was similar across treatment cohorts, with the exception of the tocilizumab cohort, where 50.0% of patients were discharged, 10.0% of patients remained hospitalized, 40.0% of patients died, and 50% of patients achieved clinical improvement.

## Discussion

This study described pre-treatment characteristics, real-world treatment patterns, and clinical and health resource use outcomes among patients diagnosed with COVID-19 in the US who initiated commonly used treatment regimens for COVID-19 in the inpatient setting between September 2020 and January 2021. Findings showed that half the patients included in this study received dexamethasone or dexamethasone + remdesivir as the index treatment. The remaining patients received azithromycin or remdesivir or combinations of dexamethasone, azithromycin, and remdesivir. Convalescent plasma or tocilizumab or combinations of convalescent plasma, hydroxychloroquine, or tocilizumab were used infrequently during this time period.

The predominant use of dexamethasone and remdesivir in this study largely reflects current NIH treatment guidelines for patients hospitalized with COVID-19. NIH guidelines recommend the use of dexamethasone and remdesivir in hospitalized patients requiring supplemental oxygen; however, some included patients received these treatments regardless of their need for oxygen therapy [18].

Treatment utilization in patients hospitalized with COVID-19 in the US has been reported in previous studies conducted earlier in the pandemic. A cohort study of 3,546 patients hospitalized with COVID-19 in California between March 10 and December 31, 2020 showed dexamethasone daily usage increased from 1.4% of patients on March 31 to 67.5% of patients on December 31, remdesivir daily usage increased from 4.9% of patients on June 1 to 62.5% of patients on December 31, and azithromycin daily usage decreased from 45.5% of patients on April 1 to 20.0% of patients on August 1. Tocilizumab daily usage remained low at <2.4% across the study period [25]. A study of treatment patterns among 53,264 patients hospitalized with COVID-19 between April 1 and July 31, 2020 using data from the Premier Healthcare Database showed a decline in the use of hydroxychloroquine (19.6% to 0.5%), a substantial increase in the use of dexamethasone (4.7% to 61.2%), and an increase in the use of remdesivir (0.4% to 10.6%) across the study period. The change in use of convalescent plasma (1.6% to 2.3%) and tocilizumab (6.7% to 3.7%) was low [26]. In a study of 2,821 patients hospitalized with COVID-19 between March 1 and May 24, 2020, EHR data from a Massachusetts-based healthcare system showed the use of most COVID-19 medications, including hydroxychloroquine and azithromycin, decreased by nearly 90%. Use of tocilizumab fluctuated below 10% during the study period and only the use of remdesivir increased significantly, likely as a result of the broadened its EUA in May 2020 [7].

In the overall study population and across all treatment cohorts, a high proportion of patients had comorbidities. Almost half of the patients had hypertension, a third of the patients had obesity, and a quarter of the patients had diabetes. Patients in the convalescent plasma and tocilizumab cohorts were more likely to have additional comorbidities. This may have resulted in more severe COVID-19 disease [27] and served as the rationale for the administration of their index treatments. Convalescent plasma and tocilizumab have primarily been administered to critically ill patients with COVID-19 [15, 28–30]. However, a recent randomized, double-blind, placebo-controlled trial of convalescent plasma with high antibody titers against SARS-CoV-2 in 160 older adults showed that infusion within 72 hours after the onset of mild symptoms reduced the progression of COVID-19 by 48% [31]. Further investigations are required to fully understand the reasons for the disparities in index treatment use by patient age, gender, race, region, and comorbidity profile identified in this study, and their effects on patient outcomes. Treatment may have been guided by various patient-level and healthcare system-level factors, including disease severity, differences in practice protocols, and the availability of medications and healthcare resources [7, 32, 33].

In the general US population, 16.5% of individuals are 65 years and over, 49.2% are male, 76.3% of Americans are White, 13.4% are Black, and 45.4% of adults have comorbidities that may increase the risk of COVID-19 complications [34, 35]. The Optum^®^ EHR database is geographically diverse across the US, with the greatest proportion of patients residing in the Midwest and South US Census Bureau regions. The age and sex distribution of the enrollees is similar to that reported by the US Census Bureau [36]. Compared to these population-based estimates, data from the present study revealed that older individuals and males were most likely to require hospitalization and receive treatment for COVID-19, and a high proportion of patients had comorbidities. No notable differences were observed in the racial composition of those hospitalized and treated compared to the US population in general. These findings are consistent with studies conducted across the US in the first 6 months of the pandemic (January–June 2020), which showed hospitalizations among patients with confirmed COVID-19 were most prevalent among the elderly, men, and those with hypertension, diabetes, obesity, cardiovascular disease, and chronic kidney disease [37–46]. Studies encompassing the period from March to October, 2020 consistently identified a higher proportion of males and those with hypertension, diabetes, and obesity among adults hospitalized with COVID-19 [47, 48].

Relatively few real-world studies examining COVID-19 treatment patterns among patients hospitalized with COVID-19 have been published [7, 25, 27, 32]. To the authors’ knowledge, no other studies have reported on longitudinal treatment patterns for patients hospitalized with COVID-19. The present study showed that dexamethasone was the most commonly used treatment at day 7 post-index among patients with COVID-19 who remained hospitalized, irrespective of index treatment, except for patients initiating regimens containing hydroxychloroquine or tocilizumab. This may reflect the use of tocilizumab as an anti-inflammatory in patients with rapidly increasing oxygen needs and systemic inflammation [49]. At day 14 post-index, the majority of patients in this study received no further treatment of interest, but may have been provided supportive care. Prevailing treatment guidelines and published case studies, as well as changes in patient COVID-19 severity status, may have served as the basis for changes in drug utilization during inpatient stays [7].

Ultimately, no index treatment regimens or post-index treatments administered to the patients in this study appeared to alleviate the inpatient morbidity and mortality associated with COVID-19. Evaluation of clinical and health resource use outcomes showed the mean length of hospital stay in the overall study population was 8.1 days, and was similar across treatment cohorts. The proportion of patients requiring ICU-level care ranged from 4.0–15.3% across treatment cohorts, and fewer than 12% of patients in all cohorts received non-invasive or invasive ventilation. At the end of the 28-day follow-up period, approximately 80% of patients were discharged alive, 4% died in hospital, and 9% died after discharge. In previous studies, the mean (SD) length of hospital stay for US patients hospitalized with COVID-19 ranged from 7.7 (10.8) to 8.5 (13.6) days, and the mean (SD) length of ICU stay ranged from 7.3 (6.8) to 9.6 (8.0) days [47, 50, 51]. The median (interquartile range, IQR) length of hospital stay for US patients hospitalized with COVID-19 ranged from 5 (3–10) to 7 (3–13) days and from 5 (2–10) to 15 (6–20) days for patients requiring ICU care [43, 47, 48, 50, 51]. ICU admission and ventilation use were higher than those in the present study, with approximately 14–23% of patients hospitalized with COVID-19 requiring ICU-level care and approximately 12–16% of patients receiving invasive mechanical ventilation [47, 48, 50, 51].

For context, the burden presented by COVID-19 appears higher than other common respiratory diseases, such as seasonal influenza, acute asthma, and acute COPD exacerbation. Compared to seasonal influenza, COVID-19 has been associated with a higher risk of mechanical ventilator use (hazard ratio [HR] 4.01, confidence interval [CI]: 3.53–4.54) and ICU admission (HR 2.41, CI: 2.25–2.59) [4], and a prolonged length of hospital stay (mean [SD],10 [10.99] days vs. 7 [19.00] days; median (IQR) range of hospital stay, 6 [3–12] to 8 [4–16] days vs. 3 [2–6] to 4 [2–7] days) [4, 52]. The median (IQR) length of hospital stay for patients with acute asthma has been estimated at 2 (1–3) days [53]. The mean (SD) range of length of hospital stay for adults hospitalized with acute COPD exacerbation has been reported at 4.5 (3.3) to 6 days, and up to 16 (16.7) days for ICU admissions [54, 55].

At the start of the pandemic, mortality rates for inpatients with COVID-19 were high (up to 33%) [7,40,44,50,51,56], but have subsequently decreased [37, 43]. A cohort study of 38,517 patients hospitalized with COVID-19 found mortality rates in US hospitals declined from 16.6% in January 2020 to 9.3% in June 2020 [56]. Another cohort study of 503,409 US patients hospitalized with COVID-19 reported inpatient mortality rates of 10.6%, 19.7%, and 9.3% for March, April, and November 2020, respectively [50]. The low inpatient mortality rate observed in the present study likely reflects continued improvements in the management of patients hospitalized with COVID-19; however, a notable proportion of patients died shortly after discharge. This finding was similar to a study of 1,648 patients hospitalized with COVID-19 in Michigan (March 2020–July 2020), where 6.7% of discharged patients died during 60 days of follow-up [57]. The significant morbidity and mortality among survivors of COVID-19 underscores the urgent need for widespread preventive measures, as well as the development of effective treatments.

## Strengths and limitations

The strength of this study is the relative stabilization of COVID-19 treatments observed over the study period (index treatment date: September 2020 to January 2021), as real-world decisions and treatment guidelines were relatively consistent during this time. Criteria and real-world decisions for hospitalization of COVID-19 patients may vary across geographies, time periods, and patient groups, and could impact inpatient outcomes. Acknowledging that many therapies are still under development, data reported here are expected to remain relevant for the near future. This study was subject to several limitations. First, centers contributing the Optum EHR database data may not be fully representative of the general COVID-19 inpatient population in the US. Second, the small sample size of some of the cohorts limits the reliability of some findings. Third, oxygen supplementation and respiratory support were identified based on procedure codes, which may be substantially under-recorded in the EHR. Therefore, clinical status identified from the EHR data may not be accurate if there are insufficient details on oxygen supplementation, ventilation, or organ support. Fourth, as the Optum EHR database is not a closed system, several data points may not have been fully captured. In particular, treatments occurring before or after hospital discharge, as well as deaths, may be missing despite best efforts to link this information from other data sources.

## Conclusion

This real-world study described patient characteristics, treatment patterns, and outcomes among hospitalized patients who initiated common treatments for COVID-19 from September 2020 to January 2021 in the US. Elderly patients and patients with comorbidities were most likely to require hospitalization for COVID-19. Dexamethasone and dexamethasone with remdesivir were the most prevalent treatments initiated by patients, and treatment was most often switched to dexamethasone for patients hospitalized for more than a week. Although the majority of patients were discharged alive, no treatment regimens appeared to alleviate the inpatient morbidity and mortality associated with COVID-19. This highlights an unmet need for effective treatment options for patients hospitalized with COVID-19.

## Supporting information

S1 Table Labour and delivery codes used as exclusion criteria. CPT, Current Procedural Terminology; ICD-10-CM, International Classification of Diseases, Tenth Revision, Clinical Modification.
(DOCX)

S2 Table Diagnosis and procedure codes used to identify patient comorbidities. a) Cardiac disease diagnosis ICD-9-CM, International Classification of Diseases, Ninth Revision, Clinical Modification; ICD-10-CM, International Classification of Diseases, Tenth Revision, Clinical Modification. b) Respiratory disease diagnosis. ARDS, acute respiratory distress syndrome; International Classification of Diseases, Tenth Revision, Clinical Modification. c) Diabetes mellitus diagnosis. International Classification of Diseases, Tenth Revision, Clinical Modification. d) Kidney disease diagnosis. International Classification of Diseases, Tenth Revision, Clinical Modification. e) Kidney disease procedures. CPT, Current Procedural Terminology; HCPCS, Healthcare Common Procedure Coding System; ICD-10-PCS, International Classification of Diseases, Tenth Revision, Procedure Coding System. f) Venous thromboembolism (VTE) diagnosis. ICD-9-CM, International Classification of Diseases, Ninth Revision, Clinical Modification; ICD-10-CM, International Classification of Diseases, Tenth Revision, Clinical Modification.
(DOCX)

S1 Fig Index treatment use per total COVID-19 patients, by month. a) Dexamethasone. b) Dexamethasone + Remdesivir. c) Azithromycin. d) Azithromycin + Dexamethasone. e) Remdesivir. f) Azithromycin + Dexamethasone + Remdesivir. g) Azithromycin + Remdesivir. h) Convalescent Plasma + Dexamethasone + Remdesivir. i) Convalescent Plasma. j) Hydroxychloroquine. k) Azithromycin + Hydroxychloroquine. l) Tocilizumab.
(TIF)

S2 Fig Post-index treatment switching. Azi, azithromycin; Dex, dexamethasone; HCQ, hydroxychloroquine; NA, not available; Rem, remdesivir.
(TIF)

## References

[1] Centers for Disease Control and Prevention COVID Data Tracker. Available at: https://covid.cdc.gov/covid-data-tracker/#cases_casesper100klast7days. Accessed June 1, 2021.

[2] Owusu D, Kim L, O’Halloran A, Whitaker M, Piasecki AM, Reingold A, et al; COVID-NET Surveillance Teama. Characteristics of adults aged 18–49 years without underlying conditions hospitalized with laboratory-confirmed coronavirus disease 2019 in the United States: COVID-NET-March-August 2020. Clin Infect Dis. 2021;72(5): e162–e166. doi: 10.1093/cid/ciaa1806 33270136 PMC7799269

[3] Nemer DM, Wilner BR, Burkle A, Aguilera J, Adewumi J, Gillombardo C, et al. Clinical characteristics and outcomes of non-ICU hospitalization for COVID-19 in a nonepicenter, centrally monitored healthcare system. J Hosp Med. 2021;16(1): 7–14. doi: 10.12788/jhm.3510 33147132 PMC7768915

[4] Xie Y, Bowe B, Maddukuri G, Al-Aly Z. Comparative evaluation of clinical manifestations and risk of death in patients admitted to hospital with Covid-19 and seasonal influenza: cohort study. BMJ. 2020; Dec 15:; 371:m4677. doi: 10.1136/bmj.m4677 ; PMCID: PMC7735416. 33323357 PMC7735416

[5] Magagnoli J, Narendran S, Pereira F, Cummings TH, Hardin JW, Sutton SS, et al. Outcomes of hydroxychloroquine usage in United States veterans hospitalized with Covid-19. medRxiv. 2020. 10.1016/j.medj.2020.06.001 PMC7274588 32838355

[6] Beyzarov E, Chen Y, Julg R, Naim K, Shah J, Gregory WW, et al. Global safety database summary of COVID-19-related drug utilization-safety surveillance: A sponsor’s perspective. Drug Saf. 2021; Jan;44(1): 95–105. doi: 10.1007/s40264-020-01035-x Epub 2020 Dec 22. ; PMCID: PMC7755229. 33354753 PMC7755229

[7] Lin KJ, Schneeweiss S, Tesfaye H, D’Andrea E, Liu J, Lii J, et al. Pharmacotherapy for hospitalized patients with COVID-19: Treatment patterns by disease severity. Drugs. 2020;80(18): 1961–1972. doi: 10.1007/s40265-020-01424-7 33151482 PMC7643089

[8] Beigel JH, Tomashek KM, Dodd LE, Mehta AK, Zingman BS, Kalil AC, et al; ACTT-1 Study Group Members. Remdesivir for the treatment of Covid-19—Final Report. N Engl J Med. 2020; Nov 5;383(19): 1813–1826. doi: 10.1056/NEJMoa2007764 Epub 2020 Oct 8. ; PMCID: PMC7262788. 32445440 PMC7262788

[9] Goldman JD, Lye DCB, Hui DS, Marks KM, Bruno R, Montejano R, et al; GS-US-540-5773 Investigators. Remdesivir for 5 or 10 days in patients with severe Covid-19. N Engl J Med. 2020; Nov 5;383(19): 1827–1837. doi: 10.1056/NEJMoa2015301 Epub 2020 May 27. ; PMCID: PMC7377062. 32459919 PMC7377062

[10] Spinner CD, Gottlieb RL, Criner GJ, Arribas López JR, Cattelan AM, Soriano Viladomiu A, et al; GS-US-540-5774 Investigators. Effect of remdesivir vs standard care on Clinical status at 11 days in patients with moderate COVID-19: A randomized clinical trial. JAMA. 2020; Sep 15;324(11): 1048–1057. doi: 10.1001/jama.2020.16349 ; PMCID: PMC7442954. 32821939 PMC7442954

[11] Coronavirus (COVID-19) Update: FDA Issues Emergency Use Authorization for Potential COVID-19 Treatment. 05/01/2020. Available at: https://www.fda.gov/news-events/press-announcements/coronavirus-covid-19-update-fda-issues-emergency-use-authorization-potential-covid-19-treatment.

[12] Food and Drug Administration. FDA Approves First Treatment for COVID-19. 10/22/2020. Available at: https://www.fda.gov/news-events/press-announcements/fda-approves-first-treatment-covid-19. Accessed June 4, 2021. Food and Drug Administration. FDA Approves First Treatment for COVID-19. 10/22/2020. Available at: https://www.fda.gov/news-events/press-announcements/fda-approves-first-treatment-covid-19.

[13] RECOVERY Collaborative Group. Dexamethasone in hospitalized patients with Covid-19. N Engl J Med. 2021; 384(8):693–704. doi: 10.1056/NEJMoa2021436 32678530 PMC7383595

[14] Rosenberg ES, Dufort EM, Udo T, Wilberschied LA, Kumar J, Tesoriero J, et al. Association of treatment with hydroxychloroquine or azithromycin with in-hospital mortality in patients with COVID-19 in New York State. JAMA. 2020;323(24): 2493–2502. doi: 10.1001/jama.2020.8630 32392282 PMC7215635

[15] Alattar R, Ibrahim TBH, Shaar SH, Abdalla S, Shukri K, Daghfal JN, et al. Tocilizumab for the treatment of severe coronavirus disease 2019. J Med Virol. 2020;92(10): 2042–2049. doi: 10.1002/jmv.25964 32369191 PMC7267594

[16] Alzghari SK, Acuna VS. Supportive treatment with tocilizumab for COVID-19: A systematic review. J Clin Virol. 2020;127: 104380. doi: 10.1016/j.jcv.2020.104380 32353761 PMC7194791

[17] Zhang S, Li L, Shen A, Chen Y, Qi Z. Rational use of tocilizumab in the treatment of novel coronavirus pneumonia. Clin Drug Investig. 2020;40(6): 511–518. doi: 10.1007/s40261-020-00917-3 32337664 PMC7183818

[18] COVID-19 Treatment Guidelines Panel. Coronavirus Disease 2019 (COVID-19) Treatment Guidelines. National Institutes of Health. 2020. Available at: https://www.covid19treatmentguidelines.nih.gov/. Accessed June 7. 34003615

[19] Food and Drug Administration. Coronavirus (COVID-19) Update: FDA Revokes Emergency Use Authorization for Chloroquine and Hydroxychloroquine. 06/15/2020. Available at: https://www.fda.gov/news-events/press-announcements/coronavirus-covid-19-update-fda-revokes-emergency-use-authorization-chloroquine-and. Accessed June 5, 2021.

[20] Food and Drug Administration. COVID-19 Update: FDA Broadens Emergency Use Authorization for Veklury (remdesivir) to Include All Hospitalized Patients for Treatment of COVID-19. 08/28/2020. Available at: https://www.fda.gov/news-events/press-announcements/covid-19-update-fda-broadens-emergency-use-authorization-veklury-remdesivir-include-all-hospitalized. Accessed June 15, 2021.

[21] Food and Drug Administration. FDA Issues Emergency Use Authorization for Convalescent Plasma as Potential Promising COVID–19 Treatment, Another Achievement in Administration’s Fight Against Pandemic. 08/23/2020. Available at: https://www.fda.gov/news-events/press-announcements/fda-issues-emergency-use-authorization-convalescent-plasma-potential-promising-covid-19-treatment. Accessed June 15, 2021.

[22] Optum de-identified COVID-19 Electronic Health Record dataset (2007–2021).

[23] U.S. Department of Health and Human Services. Guidance Regarding Methods for De-identification of Protected Health Information in Accordance with the Health Insurance Portability and Accountability Act (HIPAA) Privacy Rule. 26 Nov 2012. Available at: https://www.hhs.gov/sites/default/files/ocr/privacy/hipaa/understanding/coveredentities/De-identification/hhs_deid_guidance.pdf. Accessed 20 May 2021.

[24] U.S. Department of Health and Human Services. Other requirements relating to uses and disclosures of protected health information., 45 CFR 164.514(b)(1). 7 June 2013. Available at: https://www.govinfo.gov/content/pkg/CFR-2017-title45-vol1/pdf/CFR-2017-title45-vol1-sec164-514.pdf. Accessed June 15, 2021.

[25] Watanabe JH, Kwon J, Nan B, Abeles SR, Jia S, Mehta SR. Medication use patterns in hospitalized patients with COVID-19 in California during the pandemic. JAMA Netw Open. 2021; May 3;4(5): e2110775. doi: 10.1001/jamanetworkopen.2021.10775 . 34019090 PMC8140369

[26] Fan X, Johnson BH, Johnston SS, Elangovanraaj N, Coplan P, Khanna R. Evolving treatment patterns for hospitalized COVID-19 patients in the United States in April 2020-July 2020. Int J Gen Med. 2021;14: 267–271. doi: 10.2147/IJGM.S290118 33531828 PMC7846837

[27] Gallo Marin B, Aghagoli G, Lavine K, Yang L, Siff EJ, Chiang SS, et al. Predictors of COVID-19 severity: A literature review. Rev Med Virol. 2021;31(1): 1–10. doi: 10.1002/rmv.2146 32845042 PMC7855377

[28] Gupta S, Wang W, Hayek SS, Chan L, Mathews KS, Melamed ML, et al; STOP-COVID Investigators. Association between early treatment with tocilizumab and mortality among critically ill patients with COVID-19. JAMA Intern Med. 2021;181(1): 41–51. doi: 10.1001/jamainternmed.2020.6252 33080002 PMC7577201

[29] Smoke SM, Raja K, Hilden P, Daniel NM. Early clinical outcomes with tocilizumab for severe COVID-19: a two-centre retrospective study. Int J Antimicrob Agents. 2021; Feb;57(2): 106265. doi: 10.1016/j.ijantimicag.2020.106265 Epub 2020 Dec 15. ; PMCID: PMC7834231. 33338559 PMC7834231

[30] Salazar E, Christensen PA, Graviss EA, Nguyen DT, Castillo B, Chen J, et al. Treatment of coronavirus disease 2019 patients with convalescent plasma reveals a signal of significantly decreased mortality. Am J Pathol. 2020; 190: 2290–2303. doi: 10.1016/j.ajpath.2020.08.001 32795424 PMC7417901

[31] Libster R, Pérez Marc G, Wappner D, Coviello S, Bianchi A, Braem V, et al; Fundación INFANT–COVID-19 Group. Early High-Titer Plasma Therapy to Prevent Severe Covid-19 in Older Adults. N Engl J Med. 2021;384(7):610–618. doi: 10.1056/NEJMoa2033700 33406353 PMC7793608

[32] Best JH, Kong AM, Kaplan-Lewis E, Brawley OW, Baden R, Zazzali JL, et al. Treatment patterns in US patients hospitalized with COVID-19 and pulmonary involvement. J Med Virol. 2021; Apr 29. Available at: doi: 10.1002/jmv.27049 33913536 PMC8242555

[33] Emanuel EJ, Persad G, Upshur R, Thome B, Parker M, Glickman A, et al. Fair allocation of scarce medical resources in the time of Covid-19. N Engl J Med. 2020; May 21;382(21): 2049–2055. doi: 10.1056/NEJMsb2005114 Epub 2020 Mar 23. . 32202722

[34] Adams ML, Katz DL, Grandpre J. Population based estimates of comorbidities affecting risk for complications from COVID-19 in the US. medRxiv 2020.03.30.20043919; 10.1101/2020.03.30.20043919

[35] U.S. Census Bureau QuickFacts. 2019. Available at: https://www.census.gov/quickfacts/fact/table/US/PST045219. Accessed May 20, 2021.

[36] Canfield S, Kemeter MJ, Hornberger J, Febbo PG. Active surveillance use among a low-risk prostate cancer population in a large US payer system: 17-gene genomic prostate score versus other risk stratification methods. Rev Urol. 2017;19(4):203–212. doi: 10.3909/riu0786 ; PMCID: PMC5811877. 29472824 PMC5811877

[37] Buckner FS, McCulloch DJ, Atluri V, Blain M, McGuffin SA, Nalla AK, et al. Clinical features and outcomes of 105 hospitalized patients with COVID-19 in Seattle, Washington. Clin Infect Dis. 2020; Nov 19;71(16): 2167–2173. doi: 10.1093/cid/ciaa632 ; PMCID: PMC7314181. 32444880 PMC7314181

[38] Garg S, Kim L, Whitaker M, O’Halloran A, Cummings C, Holstein R, et al. Hospitalization rates and characteristics of patients hospitalized with laboratory-confirmed coronavirus disease 2019—COVID-NET, 14 States, March 1–30, 2020. MMWR Morb Mortal Wkly Rep. 2020;69: 458–464. doi: 10.15585/mmwr.mm6915e3 32298251 PMC7755063

[39] Goyal P, Choi JJ, Pinheiro LC, Schenck EJ, Chen R, Jabri A, et al. Clinical characteristics of Covid-19 in New York City. N Engl J Med. 2020; Jun 11;382(24): 2372–2374. doi: 10.1056/NEJMc2010419 Epub 2020 Apr 17. ; PMCID: PMC7182018. 32302078 PMC7182018

[40] Imam Z, Odish F, Gill I, O’Connor D, Armstrong J, Vanood A, et al. Older age and comorbidity are independent mortality predictors in a large cohort of 1305 COVID-19 patients in Michigan, United States. J Intern Med. 2020; Oct;288(4): 469–476. doi: 10.1111/joim.13119 Epub 2020 Jun 22. ; PMCID: PMC7300881. 32498135 PMC7300881

[41] Killerby ME, Link-Gelles R, Haight SC, Schrodt CA, England L, Gomes DJ, et al; CDC COVID-19 Response Clinical Team. Characteristics associated with hospitalization among patients with COVID-19 -—metropolitan Atlanta, Georgia, March–April 2020. MMWR Morb Mortal Wkly Rep. 2020;69: 790–794. doi: 10.15585/mmwr.mm6925e1 32584797 PMC7316317

[42] Ko JY, Danielson ML, Town M, Derado G, Greenlund KJ, Daily Kirley P, et al; COVID-NET Surveillance Team. Risk factors for COVID-19-associated hospitalization: COVID-19-Associated Hospitalization Surveillance Network and Behavioral Risk Factor Surveillance System. Clin Infect Dis. 2020; Sep 18: ciaa1419. doi: 10.1093/cid/ciaa1419 Epub ahead of print. ; PMCID: PMC7543371. 32945846 PMC7543371

[43] Petrilli CM, Jones SA, Yang J, Rajagopalan H, O’Donnell L, Chernyak Y, et al. Factors associated with hospital admission and critical illness among 5279 people with coronavirus disease 2019 in New York City: prospective cohort study. BMJ. 2020; May 22;369: m1966. doi: 10.1136/bmj.m1966 ; PMCID: PMC7243801. 32444366 PMC7243801

[44] Richardson S, Hirsch JS, Narasimhan M, Crawford JM, McGinn T, Davidson KW; the Northwell COVID-19 Research Consortium, et al. Presenting characteristics, comorbidities, and outcomes among 5700 patients hospitalized with COVID-19 in the New York City area. JAMA. 2020; May 26;323(20): 2052–2059. doi: 10.1001/jama.2020.6775 Erratum in: JAMA. 2020 May 26;323(20):2098. ; PMCID: PMC7177629. 32320003 PMC7177629

[45] Stokes EK, Zambrano LD, Anderson KN, Marder EP, Raz KM, El Burai Felix S, et al. Coronavirus disease 2019 case surveillance -—United States, January 22–May 30, 2020. MMWR Morb Mortal Wkly Rep. 2020;69: 759–765. doi: 10.15585/mmwr.mm6924e2 32555134 PMC7302472

[46] van Gerwen M, Alsen M, Little C, Barlow J, Genden E, Naymagon L, et al. Risk factors and outcomes of COVID-19 in New York City; a retrospective cohort study. J Med Virol. 2021; Feb;93(2): 907–915. doi: 10.1002/jmv.26337 Epub 2020 Aug 13. ; PMCID: PMC7404409. 32706392 PMC7404409

[47] Di Fusco M, Shea KM, Lin J, Nguyen JL, Angulo FJ, Benigno M, et al. Health outcomes and economic burden of hospitalized COVID-19 patients in the United States. J Med Econ. 2021;24(1): 308–317. doi: 10.1080/13696998.2021.1886109 33555956

[48] Nguyen NT, Chinn J, Nahmias J, Yuen S, Kirby KA, Hohmann S, et al. Outcomes and mortality among adults hospitalized with COVID-19 at US medical centers. JAMA Netw Open. 2021;4(3): e210417. doi: 10.1001/jamanetworkopen.2021.0417 33666657 PMC8547263

[49] RECOVERY Collaborative Group. Tocilizumab in patients admitted to hospital with COVID-19 (RECOVERY): a randomised, controlled, open-label, platform trial. Lancet. 2021 May 1;397(10285): 1637–1645. doi: 10.1016/S0140-6736(21)00676-0 ; PMCID: PMC8084355. 33933206 PMC8084355

[50] Finelli L, Gupta V, Petigara T, Yu K, Bauer KA, Puzniak LA. Mortality among US patients hospitalized with SARS-CoV-2 infection in 2020. JAMA Netw Open. 2021; Apr 1;4(4): e216556. doi: 10.1001/jamanetworkopen.2021.6556 ; PMCID: PMC8033442. 33830226 PMC8033442

[51] Rosenthal N, Cao Z, Gundrum J, Sianis J, Safo S. Risk factors associated with in-hospital mortality in a US national sample of patients with COVID-19. JAMA Netw Open. 2020 Dec 1;3(12):e2029058. doi: 10.1001/jamanetworkopen.2020.29058 Erratum in: JAMA Netw Open. 2021; Jan 4;4(1): e2036103. ; PMCID: PMC7729428. 33301018 PMC7729428

[52] Donnino MW, Moskowitz A, Thompson GS, Heydrick SJ, Pawar RD, Berg KM, et al. Comparison between patients hospitalized with influenza and COVID-19 at a tertiary care center. J Gen Intern Med. 2021; Mar 18: 1–7. doi: 10.1007/s11606-021-06647-2 Epub ahead of print. ; PMCID: PMC7971402. 33738759 PMC7971402

[53] Hasegawa K, Tsugawa Y, Clark S, Eastin CD, Gabriel S, Herrera V, et al; MARC-37 Investigators. Improving quality of acute asthma care in US hospitals: changes between 1999–2000 and 2012–2013. Chest. 2016; Jul;150(1): 112–22. doi: 10.1016/j.chest.2016.03.037 Epub 2016 Apr 4. ; PMCID: PMC6026245. 27056585 PMC6026245

[54] Lima FV, Yen TY, Patel JK. Trends in in-hospital outcomes among adults hospitalized with exacerbation of chronic obstructive pulmonary disease. COPD. 2015; 12(6): 636–42. doi: 10.3109/15412555.2015.1020151 Epub 2015 Aug 11. . 26263035

[55] Dalal AA, Shah M, D’Souza AO, Rane P. Costs of COPD exacerbations in the emergency department and inpatient setting. Respir Med. 2011; Mar;105(3): 454–60. doi: 10.1016/j.rmed.2010.09.003 . 20869226

[56] Asch DA, Sheils NE, Islam MN, Chen Y, Werner RM, Buresh J, et al. Variation in US hospital mortality rates for patients admitted with COVID-19 during the first 6 months of the pandemic. JAMA Intern Med. 2021; Apr 1;181(4): 471–478. doi: 10.1001/jamainternmed.2020.8193 ; PMCID: PMC7756246. 33351068 PMC7756246

[57] Chopra V, Flanders SA, O’Malley M, Malani AN, Prescott HC. Sixty-day outcomes among patients hospitalized with COVID-19. Ann Intern Med. 20201; 174(4): 576–578. doi: 10.7326/M20-5661 33175566 PMC7707210

