# Supplementary material for: Real-world treatment patterns and clinical outcomes for inpatients with COVID-19 in the US from September 2020 to February 2021
Source: PLoS One. 2021 Dec 28;16(12):e0261707. doi: 10.1371/journal.pone.0261707 (PMC8714107; doi:10.1371/journal.pone.0261707)
Supplement: S1 Table — CPT, Current Procedural Terminology; ICD-10-CM, International Classification of Diseases, Tenth Revision, Clinical Modification. (DOCX) [file pone.0261707.s001.docx]

## Table S1. Labour and delivery codes used as exclusion criteria

| **Criterion Name** | **Code Type** | **Code** | **Code Description** |
| --- | --- | --- | --- |
| childbirth | CPT | 01960 | Anesthesia for vaginal delivery only |
| childbirth | CPT | 01961 | Anesthesia for cesarean delivery only |
| childbirth | CPT | 01967 | Neuraxial labor analgesia/anesthesia for planned vaginal delivery (this includes any repeat subarachnoid needle placement and drug injection and/or any necessary replacement of an epidural catheter during labor) |
| childbirth | CPT | 01968 | Anesthesia for cesarean delivery following neuraxial labor analgesia/anesthesia (list separately in addition to code for primary procedure performed) |
| childbirth | CPT | 58611 | Ligation or transection of fallopian tube(s) when done at the time of cesarean delivery or intra-abdominal surgery (not a separate procedure) (list separately in addition to code for primary procedure) |
| childbirth | CPT | 59409 | 59409: Vaginal delivery only (with or without episiotomy and/or forceps) |
| childbirth | CPT | 59410 | 59410: Vaginal delivery only (with or without episiotomy and/or forceps); including postpartum care |
| childbirth | CPT | 59514 | 59514: Cesarean delivery only |
| childbirth | CPT | 59515 | 59515: Cesarean delivery only; including postpartum care |
| childbirth | CPT | 59525 | 59525: Subtotal or total hysterectomy after cesarean delivery (List separately in addition to code for primary procedure) |
| childbirth | CPT | 59612 | 59612: Vaginal delivery only, after previous cesarean delivery (with or without episiotomy and/or forceps) |
| childbirth | CPT | 59614 | 59614: Vaginal delivery only, after previous cesarean delivery (with or without episiotomy and/or forceps); including postpartum care |
| childbirth | CPT | 59620 | 59620: Cesarean delivery only, following attempted vaginal delivery after previous cesarean delivery |
| childbirth | CPT | 59622 | 59622: Cesarean delivery only, following attempted vaginal delivery after previous cesarean delivery; including postpartum care |
| childbirth | ICD-10-CM Procedure | 10D00Z0 | 10D00Z0: Extraction of Products of Conception, High, Open Approach |
| childbirth | ICD-10-CM Procedure | 10D00Z1 | 10D00Z1: Extraction of Products of Conception, Low, Open Approach |
| childbirth | ICD-10-CM Procedure | 10D00Z2 | 10D00Z2: Extraction of Products of Conception, Extraperitoneal, Open Approach |
| childbirth | ICD-10-CM Procedure | 10D07Z3 | 10D07Z3: Extraction of Products of Conception, Low Forceps, Via Natural or Artificial Opening |
| childbirth | ICD-10-CM Procedure | 10D07Z4 | 10D07Z4: Extraction of Products of Conception, Mid Forceps, Via Natural or Artificial Opening |
| childbirth | ICD-10-CM Procedure | 10D07Z5 | 10D07Z5: Extraction of Products of Conception, High Forceps, Via Natural or Artificial Opening |
| childbirth | ICD-10-CM Procedure | 10D07Z6 | 10D07Z6: Extraction of Products of Conception, Vacuum, Via Natural or Artificial Opening |
| childbirth | ICD-10-CM Procedure | 10D07Z7 | 10D07Z7: Extraction of Products of Conception, Internal Version, Via Natural or Artificial Opening |
| childbirth | ICD-10-CM Procedure | 10D07Z8 | 10D07Z8: Extraction of Products of Conception, Other, Via Natural or Artificial Opening |
| childbirth | ICD-10-CM Procedure | 10E0XZZ | 10E0XZZ: Delivery of Products of Conception, External Approach |
| childbirth | REV | "0722" | 0722: Labor Room/Delivery - Delivery |
| childbirth | ICD-10-CM Diagnosis | O10.02 | Pre-existing essential hypertension complicating childbirth |
| childbirth | ICD-10-CM Diagnosis | O10.12 | Pre-existing hypertensive heart disease complicating childbirth |
| childbirth | ICD-10-CM Diagnosis | O10.22 | Pre-existing hypertensive chronic kidney disease complicating childbirth |
| childbirth | ICD-10-CM Diagnosis | O10.32 | Pre-existing hypertensive heart and chronic kidney disease complicating childbirth |
| childbirth | ICD-10-CM Diagnosis | O10.42 | Pre-existing secondary hypertension complicating childbirth |
| childbirth | ICD-10-CM Diagnosis | O10.92 | Unspecified pre-existing hypertension complicating childbirth |
| childbirth | ICD-10-CM Diagnosis | O11.4 | Pre-existing hypertension with pre-eclampsia, complicating childbirth |
| childbirth | ICD-10-CM Diagnosis | O12.04 | Gestational edema, complicating childbirth |
| childbirth | ICD-10-CM Diagnosis | O12.14 | Gestational proteinuria, complicating childbirth |
| childbirth | ICD-10-CM Diagnosis | O12.24 | Gestational edema with proteinuria, complicating childbirth |
| childbirth | ICD-10-CM Diagnosis | O13.4 | Gestational [pregnancy-induced] hypertension without significant proteinuria, complicating childbirth |
| childbirth | ICD-10-CM Diagnosis | O14.04 | Mild to moderate pre-eclampsia, complicating childbirth |
| childbirth | ICD-10-CM Diagnosis | O14.14 | Severe pre-eclampsia complicating childbirth |
| childbirth | ICD-10-CM Diagnosis | O14.24 | HELLP syndrome, complicating childbirth |
| childbirth | ICD-10-CM Diagnosis | O14.94 | Unspecified pre-eclampsia, complicating childbirth |
| childbirth | ICD-10-CM Diagnosis | O16.4 | Unspecified maternal hypertension, complicating childbirth |
| childbirth | ICD-10-CM Diagnosis | O24.02 | Pre-existing type 1 diabetes mellitus, in childbirth |
| childbirth | ICD-10-CM Diagnosis | O24.12 | Pre-existing type 2 diabetes mellitus, in childbirth |
| childbirth | ICD-10-CM Diagnosis | O24.32 | Unspecified pre-existing diabetes mellitus in childbirth |
| childbirth | ICD-10-CM Diagnosis | O24.42 | Gestational diabetes mellitus in childbirth |
| childbirth | ICD-10-CM Diagnosis | O24.420 | Gestational diabetes mellitus in childbirth, diet controlled |
| childbirth | ICD-10-CM Diagnosis | O24.424 | Gestational diabetes mellitus in childbirth, insulin controlled |
| childbirth | ICD-10-CM Diagnosis | O24.425 | Gestational diabetes mellitus in childbirth, controlled by oral hypoglycemic drugs |
| childbirth | ICD-10-CM Diagnosis | O24.429 | Gestational diabetes mellitus in childbirth, unspecified control |
| childbirth | ICD-10-CM Diagnosis | O24.82 | Other pre-existing diabetes mellitus in childbirth |
| childbirth | ICD-10-CM Diagnosis | O24.92 | Unspecified diabetes mellitus in childbirth |
| childbirth | ICD-10-CM Diagnosis | O25.2 | Malnutrition in childbirth |
| childbirth | ICD-10-CM Diagnosis | O26.62 | Liver and biliary tract disorders in childbirth |
| childbirth | ICD-10-CM Diagnosis | O26.72 | Subluxation of symphysis (pubis) in childbirth |
| childbirth | ICD-10-CM Diagnosis | O60.1 | Preterm labor with preterm delivery |
| childbirth | ICD-10-CM Diagnosis | O60.10 | Preterm labor with preterm delivery, unspecified trimester |
| childbirth | ICD-10-CM Diagnosis | O60.10X0 | Preterm labor with preterm delivery, unspecified trimester, not applicable or unspecified |
| childbirth | ICD-10-CM Diagnosis | O60.10X1 | Preterm labor with preterm delivery, unspecified trimester, fetus 1 |
| childbirth | ICD-10-CM Diagnosis | O60.10X2 | Preterm labor with preterm delivery, unspecified trimester, fetus 2 |
| childbirth | ICD-10-CM Diagnosis | O60.10X3 | Preterm labor with preterm delivery, unspecified trimester, fetus 3 |
| childbirth | ICD-10-CM Diagnosis | O60.10X4 | Preterm labor with preterm delivery, unspecified trimester, fetus 4 |
| childbirth | ICD-10-CM Diagnosis | O60.10X5 | Preterm labor with preterm delivery, unspecified trimester, fetus 5 |
| childbirth | ICD-10-CM Diagnosis | O60.10X9 | Preterm labor with preterm delivery, unspecified trimester, other fetus |
| childbirth | ICD-10-CM Diagnosis | O60.12 | Preterm labor second trimester with preterm delivery second trimester |
| childbirth | ICD-10-CM Diagnosis | O60.12X0 | Preterm labor second trimester with preterm delivery second trimester, not applicable or unspecified |
| childbirth | ICD-10-CM Diagnosis | O60.12X1 | Preterm labor second trimester with preterm delivery second trimester, fetus 1 |
| childbirth | ICD-10-CM Diagnosis | O60.12X2 | Preterm labor second trimester with preterm delivery second trimester, fetus 2 |
| childbirth | ICD-10-CM Diagnosis | O60.12X3 | Preterm labor second trimester with preterm delivery second trimester, fetus 3 |
| childbirth | ICD-10-CM Diagnosis | O60.12X4 | Preterm labor second trimester with preterm delivery second trimester, fetus 4 |
| childbirth | ICD-10-CM Diagnosis | O60.12X5 | Preterm labor second trimester with preterm delivery second trimester, fetus 5 |
| childbirth | ICD-10-CM Diagnosis | O60.12X9 | Preterm labor second trimester with preterm delivery second trimester, other fetus |
| childbirth | ICD-10-CM Diagnosis | O60.13 | Preterm labor second trimester with preterm delivery third trimester |
| childbirth | ICD-10-CM Diagnosis | O60.13X0 | Preterm labor second trimester with preterm delivery third trimester, not applicable or unspecified |
| childbirth | ICD-10-CM Diagnosis | O60.13X1 | Preterm labor second trimester with preterm delivery third trimester, fetus 1 |
| childbirth | ICD-10-CM Diagnosis | O60.13X2 | Preterm labor second trimester with preterm delivery third trimester, fetus 2 |
| childbirth | ICD-10-CM Diagnosis | O60.13X3 | Preterm labor second trimester with preterm delivery third trimester, fetus 3 |
| childbirth | ICD-10-CM Diagnosis | O60.13X4 | Preterm labor second trimester with preterm delivery third trimester, fetus 4 |
| childbirth | ICD-10-CM Diagnosis | O60.13X5 | Preterm labor second trimester with preterm delivery third trimester, fetus 5 |
| childbirth | ICD-10-CM Diagnosis | O60.13X9 | Preterm labor second trimester with preterm delivery third trimester, other fetus |
| childbirth | ICD-10-CM Diagnosis | O60.14 | Preterm labor third trimester with preterm delivery third trimester |
| childbirth | ICD-10-CM Diagnosis | O60.14X0 | Preterm labor third trimester with preterm delivery third trimester, not applicable or unspecified |
| childbirth | ICD-10-CM Diagnosis | O60.14X1 | Preterm labor third trimester with preterm delivery third trimester, fetus 1 |
| childbirth | ICD-10-CM Diagnosis | O60.14X2 | Preterm labor third trimester with preterm delivery third trimester, fetus 2 |
| childbirth | ICD-10-CM Diagnosis | O60.14X3 | Preterm labor third trimester with preterm delivery third trimester, fetus 3 |
| childbirth | ICD-10-CM Diagnosis | O60.14X4 | Preterm labor third trimester with preterm delivery third trimester, fetus 4 |
| childbirth | ICD-10-CM Diagnosis | O60.14X5 | Preterm labor third trimester with preterm delivery third trimester, fetus 5 |
| childbirth | ICD-10-CM Diagnosis | O60.14X9 | Preterm labor third trimester with preterm delivery third trimester, other fetus |
| childbirth | ICD-10-CM Diagnosis | O60.2 | Term delivery with preterm labor |
| childbirth | ICD-10-CM Diagnosis | O60.20 | Term delivery with preterm labor, unspecified trimester |
| childbirth | ICD-10-CM Diagnosis | O60.20X0 | Term delivery with preterm labor, unspecified trimester, not applicable or unspecified |
| childbirth | ICD-10-CM Diagnosis | O60.20X1 | Term delivery with preterm labor, unspecified trimester, fetus 1 |
| childbirth | ICD-10-CM Diagnosis | O60.20X2 | Term delivery with preterm labor, unspecified trimester, fetus 2 |
| childbirth | ICD-10-CM Diagnosis | O60.20X3 | Term delivery with preterm labor, unspecified trimester, fetus 3 |
| childbirth | ICD-10-CM Diagnosis | O60.20X4 | Term delivery with preterm labor, unspecified trimester, fetus 4 |
| childbirth | ICD-10-CM Diagnosis | O60.20X5 | Term delivery with preterm labor, unspecified trimester, fetus 5 |
| childbirth | ICD-10-CM Diagnosis | O60.20X9 | Term delivery with preterm labor, unspecified trimester, other fetus |
| childbirth | ICD-10-CM Diagnosis | O60.22 | Term delivery with preterm labor, second trimester |
| childbirth | ICD-10-CM Diagnosis | O60.22X0 | Term delivery with preterm labor, second trimester, not applicable or unspecified |
| childbirth | ICD-10-CM Diagnosis | O60.22X1 | Term delivery with preterm labor, second trimester, fetus 1 |
| childbirth | ICD-10-CM Diagnosis | O60.22X2 | Term delivery with preterm labor, second trimester, fetus 2 |
| childbirth | ICD-10-CM Diagnosis | O60.22X3 | Term delivery with preterm labor, second trimester, fetus 3 |
| childbirth | ICD-10-CM Diagnosis | O60.22X4 | Term delivery with preterm labor, second trimester, fetus 4 |
| childbirth | ICD-10-CM Diagnosis | O60.22X5 | Term delivery with preterm labor, second trimester, fetus 5 |
| childbirth | ICD-10-CM Diagnosis | O60.22X9 | Term delivery with preterm labor, second trimester, other fetus |
| childbirth | ICD-10-CM Diagnosis | O60.23 | Term delivery with preterm labor, third trimester |
| childbirth | ICD-10-CM Diagnosis | O60.23X0 | Term delivery with preterm labor, third trimester, not applicable or unspecified |
| childbirth | ICD-10-CM Diagnosis | O60.23X1 | Term delivery with preterm labor, third trimester, fetus 1 |
| childbirth | ICD-10-CM Diagnosis | O60.23X2 | Term delivery with preterm labor, third trimester, fetus 2 |
| childbirth | ICD-10-CM Diagnosis | O60.23X3 | Term delivery with preterm labor, third trimester, fetus 3 |
| childbirth | ICD-10-CM Diagnosis | O60.23X4 | Term delivery with preterm labor, third trimester, fetus 4 |
| childbirth | ICD-10-CM Diagnosis | O60.23X5 | Term delivery with preterm labor, third trimester, fetus 5 |
| childbirth | ICD-10-CM Diagnosis | O60.23X9 | Term delivery with preterm labor, third trimester, other fetus |
| childbirth | ICD-10-CM Diagnosis | O62 | Abnormalities of forces of labor |
| childbirth | ICD-10-CM Diagnosis | O62.0 | Primary inadequate contractions |
| childbirth | ICD-10-CM Diagnosis | O62.1 | Secondary uterine inertia |
| childbirth | ICD-10-CM Diagnosis | O62.2 | Other uterine inertia |
| childbirth | ICD-10-CM Diagnosis | O62.3 | Precipitate labor |
| childbirth | ICD-10-CM Diagnosis | O62.4 | Hypertonic, incoordinate, and prolonged uterine contractions |
| childbirth | ICD-10-CM Diagnosis | O62.8 | Other abnormalities of forces of labor |
| childbirth | ICD-10-CM Diagnosis | O62.9 | Abnormality of forces of labor, unspecified |
| childbirth | ICD-10-CM Diagnosis | O63 | Long labor |
| childbirth | ICD-10-CM Diagnosis | O63.0 | Prolonged first stage (of labor) |
| childbirth | ICD-10-CM Diagnosis | O63.1 | Prolonged second stage (of labor) |
| childbirth | ICD-10-CM Diagnosis | O63.2 | Delayed delivery of second twin, triplet, etc. |
| childbirth | ICD-10-CM Diagnosis | O63.9 | Long labor, unspecified |
| childbirth | ICD-10-CM Diagnosis | O64 | Obstructed labor due to malposition and malpresentation of fetus |
| childbirth | ICD-10-CM Diagnosis | O64.0 | Obstructed labor due to incomplete rotation of fetal head |
| childbirth | ICD-10-CM Diagnosis | O64.0XX0 | Obstructed labor due to incomplete rotation of fetal head, not applicable or unspecified |
| childbirth | ICD-10-CM Diagnosis | O64.0XX1 | Obstructed labor due to incomplete rotation of fetal head, fetus 1 |
| childbirth | ICD-10-CM Diagnosis | O64.0XX2 | Obstructed labor due to incomplete rotation of fetal head, fetus 2 |
| childbirth | ICD-10-CM Diagnosis | O64.0XX3 | Obstructed labor due to incomplete rotation of fetal head, fetus 3 |
| childbirth | ICD-10-CM Diagnosis | O64.0XX4 | Obstructed labor due to incomplete rotation of fetal head, fetus 4 |
| childbirth | ICD-10-CM Diagnosis | O64.0XX5 | Obstructed labor due to incomplete rotation of fetal head, fetus 5 |
| childbirth | ICD-10-CM Diagnosis | O64.0XX9 | Obstructed labor due to incomplete rotation of fetal head, other fetus |
| childbirth | ICD-10-CM Diagnosis | O64.1 | Obstructed labor due to breech presentation |
| childbirth | ICD-10-CM Diagnosis | O64.1XX0 | Obstructed labor due to breech presentation, not applicable or unspecified |
| childbirth | ICD-10-CM Diagnosis | O64.1XX1 | Obstructed labor due to breech presentation, fetus 1 |
| childbirth | ICD-10-CM Diagnosis | O64.1XX2 | Obstructed labor due to breech presentation, fetus 2 |
| childbirth | ICD-10-CM Diagnosis | O64.1XX3 | Obstructed labor due to breech presentation, fetus 3 |
| childbirth | ICD-10-CM Diagnosis | O64.1XX4 | Obstructed labor due to breech presentation, fetus 4 |
| childbirth | ICD-10-CM Diagnosis | O64.1XX5 | Obstructed labor due to breech presentation, fetus 5 |
| childbirth | ICD-10-CM Diagnosis | O64.1XX9 | Obstructed labor due to breech presentation, other fetus |
| childbirth | ICD-10-CM Diagnosis | O64.2 | Obstructed labor due to face presentation |
| childbirth | ICD-10-CM Diagnosis | O64.2XX0 | Obstructed labor due to face presentation, not applicable or unspecified |
| childbirth | ICD-10-CM Diagnosis | O64.2XX1 | Obstructed labor due to face presentation, fetus 1 |
| childbirth | ICD-10-CM Diagnosis | O64.2XX2 | Obstructed labor due to face presentation, fetus 2 |
| childbirth | ICD-10-CM Diagnosis | O64.2XX3 | Obstructed labor due to face presentation, fetus 3 |
| childbirth | ICD-10-CM Diagnosis | O64.2XX4 | Obstructed labor due to face presentation, fetus 4 |
| childbirth | ICD-10-CM Diagnosis | O64.2XX5 | Obstructed labor due to face presentation, fetus 5 |
| childbirth | ICD-10-CM Diagnosis | O64.2XX9 | Obstructed labor due to face presentation, other fetus |
| childbirth | ICD-10-CM Diagnosis | O64.3 | Obstructed labor due to brow presentation |
| childbirth | ICD-10-CM Diagnosis | O64.3XX0 | Obstructed labor due to brow presentation, not applicable or unspecified |
| childbirth | ICD-10-CM Diagnosis | O64.3XX1 | Obstructed labor due to brow presentation, fetus 1 |
| childbirth | ICD-10-CM Diagnosis | O64.3XX2 | Obstructed labor due to brow presentation, fetus 2 |
| childbirth | ICD-10-CM Diagnosis | O64.3XX3 | Obstructed labor due to brow presentation, fetus 3 |
| childbirth | ICD-10-CM Diagnosis | O64.3XX4 | Obstructed labor due to brow presentation, fetus 4 |
| childbirth | ICD-10-CM Diagnosis | O64.3XX5 | Obstructed labor due to brow presentation, fetus 5 |
| childbirth | ICD-10-CM Diagnosis | O64.3XX9 | Obstructed labor due to brow presentation, other fetus |
| childbirth | ICD-10-CM Diagnosis | O64.4 | Obstructed labor due to shoulder presentation |
| childbirth | ICD-10-CM Diagnosis | O64.4XX0 | Obstructed labor due to shoulder presentation, not applicable or unspecified |
| childbirth | ICD-10-CM Diagnosis | O64.4XX1 | Obstructed labor due to shoulder presentation, fetus 1 |
| childbirth | ICD-10-CM Diagnosis | O64.4XX2 | Obstructed labor due to shoulder presentation, fetus 2 |
| childbirth | ICD-10-CM Diagnosis | O64.4XX3 | Obstructed labor due to shoulder presentation, fetus 3 |
| childbirth | ICD-10-CM Diagnosis | O64.4XX4 | Obstructed labor due to shoulder presentation, fetus 4 |
| childbirth | ICD-10-CM Diagnosis | O64.4XX5 | Obstructed labor due to shoulder presentation, fetus 5 |
| childbirth | ICD-10-CM Diagnosis | O64.4XX9 | Obstructed labor due to shoulder presentation, other fetus |
| childbirth | ICD-10-CM Diagnosis | O64.5 | Obstructed labor due to compound presentation |
| childbirth | ICD-10-CM Diagnosis | O64.5XX0 | Obstructed labor due to compound presentation, not applicable or unspecified |
| childbirth | ICD-10-CM Diagnosis | O64.5XX1 | Obstructed labor due to compound presentation, fetus 1 |
| childbirth | ICD-10-CM Diagnosis | O64.5XX2 | Obstructed labor due to compound presentation, fetus 2 |
| childbirth | ICD-10-CM Diagnosis | O64.5XX3 | Obstructed labor due to compound presentation, fetus 3 |
| childbirth | ICD-10-CM Diagnosis | O64.5XX4 | Obstructed labor due to compound presentation, fetus 4 |
| childbirth | ICD-10-CM Diagnosis | O64.5XX5 | Obstructed labor due to compound presentation, fetus 5 |
| childbirth | ICD-10-CM Diagnosis | O64.5XX9 | Obstructed labor due to compound presentation, other fetus |
| childbirth | ICD-10-CM Diagnosis | O64.8 | Obstructed labor due to other malposition and malpresentation |
| childbirth | ICD-10-CM Diagnosis | O64.8XX0 | Obstructed labor due to other malposition and malpresentation, not applicable or unspecified |
| childbirth | ICD-10-CM Diagnosis | O64.8XX1 | Obstructed labor due to other malposition and malpresentation, fetus 1 |
| childbirth | ICD-10-CM Diagnosis | O64.8XX2 | Obstructed labor due to other malposition and malpresentation, fetus 2 |
| childbirth | ICD-10-CM Diagnosis | O64.8XX3 | Obstructed labor due to other malposition and malpresentation, fetus 3 |
| childbirth | ICD-10-CM Diagnosis | O64.8XX4 | Obstructed labor due to other malposition and malpresentation, fetus 4 |
| childbirth | ICD-10-CM Diagnosis | O64.8XX5 | Obstructed labor due to other malposition and malpresentation, fetus 5 |
| childbirth | ICD-10-CM Diagnosis | O64.8XX9 | Obstructed labor due to other malposition and malpresentation, other fetus |
| childbirth | ICD-10-CM Diagnosis | O64.9 | Obstructed labor due to malposition and malpresentation, unspecified |
| childbirth | ICD-10-CM Diagnosis | O64.9XX0 | Obstructed labor due to malposition and malpresentation, unspecified, not applicable or unspecified |
| childbirth | ICD-10-CM Diagnosis | O64.9XX1 | Obstructed labor due to malposition and malpresentation, unspecified, fetus 1 |
| childbirth | ICD-10-CM Diagnosis | O64.9XX2 | Obstructed labor due to malposition and malpresentation, unspecified, fetus 2 |
| childbirth | ICD-10-CM Diagnosis | O64.9XX3 | Obstructed labor due to malposition and malpresentation, unspecified, fetus 3 |
| childbirth | ICD-10-CM Diagnosis | O64.9XX4 | Obstructed labor due to malposition and malpresentation, unspecified, fetus 4 |
| childbirth | ICD-10-CM Diagnosis | O64.9XX5 | Obstructed labor due to malposition and malpresentation, unspecified, fetus 5 |
| childbirth | ICD-10-CM Diagnosis | O64.9XX9 | Obstructed labor due to malposition and malpresentation, unspecified, other fetus |
| childbirth | ICD-10-CM Diagnosis | O65 | Obstructed labor due to maternal pelvic abnormality |
| childbirth | ICD-10-CM Diagnosis | O65.0 | Obstructed labor due to deformed pelvis |
| childbirth | ICD-10-CM Diagnosis | O65.1 | Obstructed labor due to generally contracted pelvis |
| childbirth | ICD-10-CM Diagnosis | O65.2 | Obstructed labor due to pelvic inlet contraction |
| childbirth | ICD-10-CM Diagnosis | O65.3 | Obstructed labor due to pelvic outlet and mid-cavity contraction |
| childbirth | ICD-10-CM Diagnosis | O65.4 | Obstructed labor due to fetopelvic disproportion, unspecified |
| childbirth | ICD-10-CM Diagnosis | O65.5 | Obstructed labor due to abnormality of maternal pelvic organs |
| childbirth | ICD-10-CM Diagnosis | O65.8 | Obstructed labor due to other maternal pelvic abnormalities |
| childbirth | ICD-10-CM Diagnosis | O65.9 | Obstructed labor due to maternal pelvic abnormality, unspecified |
| childbirth | ICD-10-CM Diagnosis | O66 | Other obstructed labor |
| childbirth | ICD-10-CM Diagnosis | O66.0 | Obstructed labor due to shoulder dystocia |
| childbirth | ICD-10-CM Diagnosis | O66.1 | Obstructed labor due to locked twins |
| childbirth | ICD-10-CM Diagnosis | O66.2 | Obstructed labor due to unusually large fetus |
| childbirth | ICD-10-CM Diagnosis | O66.3 | Obstructed labor due to other abnormalities of fetus |
| childbirth | ICD-10-CM Diagnosis | O66.4 | Failed trial of labor |
| childbirth | ICD-10-CM Diagnosis | O66.40 | Failed trial of labor, unspecified |
| childbirth | ICD-10-CM Diagnosis | O66.41 | Failed attempted vaginal birth after previous cesarean delivery |
| childbirth | ICD-10-CM Diagnosis | O66.5 | Attempted application of vacuum extractor and forceps |
| childbirth | ICD-10-CM Diagnosis | O66.6 | Obstructed labor due to other multiple fetuses |
| childbirth | ICD-10-CM Diagnosis | O66.8 | Other specified obstructed labor |
| childbirth | ICD-10-CM Diagnosis | O66.9 | Obstructed labor, unspecified |
| childbirth | ICD-10-CM Diagnosis | O67 | Labor and delivery complicated by intrapartum hemorrhage, not elsewhere classified |
| childbirth | ICD-10-CM Diagnosis | O67.0 | Intrapartum hemorrhage with coagulation defect |
| childbirth | ICD-10-CM Diagnosis | O67.8 | Other intrapartum hemorrhage |
| childbirth | ICD-10-CM Diagnosis | O67.9 | Intrapartum hemorrhage, unspecified |
| childbirth | ICD-10-CM Diagnosis | O68 | Labor and delivery complicated by abnormality of fetal acid-base balance |
| childbirth | ICD-10-CM Diagnosis | O69 | Labor and delivery complicated by umbilical cord complications |
| childbirth | ICD-10-CM Diagnosis | O69.0 | Labor and delivery complicated by prolapse of cord |
| childbirth | ICD-10-CM Diagnosis | O69.0XX0 | Labor and delivery complicated by prolapse of cord, not applicable or unspecified |
| childbirth | ICD-10-CM Diagnosis | O69.0XX1 | Labor and delivery complicated by prolapse of cord, fetus 1 |
| childbirth | ICD-10-CM Diagnosis | O69.0XX2 | Labor and delivery complicated by prolapse of cord, fetus 2 |
| childbirth | ICD-10-CM Diagnosis | O69.0XX3 | Labor and delivery complicated by prolapse of cord, fetus 3 |
| childbirth | ICD-10-CM Diagnosis | O69.0XX4 | Labor and delivery complicated by prolapse of cord, fetus 4 |
| childbirth | ICD-10-CM Diagnosis | O69.0XX5 | Labor and delivery complicated by prolapse of cord, fetus 5 |
| childbirth | ICD-10-CM Diagnosis | O69.0XX9 | Labor and delivery complicated by prolapse of cord, other fetus |
| childbirth | ICD-10-CM Diagnosis | O69.1 | Labor and delivery complicated by cord around neck, with compression |
| childbirth | ICD-10-CM Diagnosis | O69.1XX0 | Labor and delivery complicated by cord around neck, with compression, not applicable or unspecified |
| childbirth | ICD-10-CM Diagnosis | O69.1XX1 | Labor and delivery complicated by cord around neck, with compression, fetus 1 |
| childbirth | ICD-10-CM Diagnosis | O69.1XX2 | Labor and delivery complicated by cord around neck, with compression, fetus 2 |
| childbirth | ICD-10-CM Diagnosis | O69.1XX3 | Labor and delivery complicated by cord around neck, with compression, fetus 3 |
| childbirth | ICD-10-CM Diagnosis | O69.1XX4 | Labor and delivery complicated by cord around neck, with compression, fetus 4 |
| childbirth | ICD-10-CM Diagnosis | O69.1XX5 | Labor and delivery complicated by cord around neck, with compression, fetus 5 |
| childbirth | ICD-10-CM Diagnosis | O69.1XX9 | Labor and delivery complicated by cord around neck, with compression, other fetus |
| childbirth | ICD-10-CM Diagnosis | O69.2 | Labor and delivery complicated by other cord entanglement, with compression |
| childbirth | ICD-10-CM Diagnosis | O69.2XX0 | Labor and delivery complicated by other cord entanglement, with compression, not applicable or unspecified |
| childbirth | ICD-10-CM Diagnosis | O69.2XX1 | Labor and delivery complicated by other cord entanglement, with compression, fetus 1 |
| childbirth | ICD-10-CM Diagnosis | O69.2XX2 | Labor and delivery complicated by other cord entanglement, with compression, fetus 2 |
| childbirth | ICD-10-CM Diagnosis | O69.2XX3 | Labor and delivery complicated by other cord entanglement, with compression, fetus 3 |
| childbirth | ICD-10-CM Diagnosis | O69.2XX4 | Labor and delivery complicated by other cord entanglement, with compression, fetus 4 |
| childbirth | ICD-10-CM Diagnosis | O69.2XX5 | Labor and delivery complicated by other cord entanglement, with compression, fetus 5 |
| childbirth | ICD-10-CM Diagnosis | O69.2XX9 | Labor and delivery complicated by other cord entanglement, with compression, other fetus |
| childbirth | ICD-10-CM Diagnosis | O69.3 | Labor and delivery complicated by short cord |
| childbirth | ICD-10-CM Diagnosis | O69.3XX0 | Labor and delivery complicated by short cord, not applicable or unspecified |
| childbirth | ICD-10-CM Diagnosis | O69.3XX1 | Labor and delivery complicated by short cord, fetus 1 |
| childbirth | ICD-10-CM Diagnosis | O69.3XX2 | Labor and delivery complicated by short cord, fetus 2 |
| childbirth | ICD-10-CM Diagnosis | O69.3XX3 | Labor and delivery complicated by short cord, fetus 3 |
| childbirth | ICD-10-CM Diagnosis | O69.3XX4 | Labor and delivery complicated by short cord, fetus 4 |
| childbirth | ICD-10-CM Diagnosis | O69.3XX5 | Labor and delivery complicated by short cord, fetus 5 |
| childbirth | ICD-10-CM Diagnosis | O69.3XX9 | Labor and delivery complicated by short cord, other fetus |
| childbirth | ICD-10-CM Diagnosis | O69.4 | Labor and delivery complicated by vasa previa |
| childbirth | ICD-10-CM Diagnosis | O69.4XX0 | Labor and delivery complicated by vasa previa, not applicable or unspecified |
| childbirth | ICD-10-CM Diagnosis | O69.4XX1 | Labor and delivery complicated by vasa previa, fetus 1 |
| childbirth | ICD-10-CM Diagnosis | O69.4XX2 | Labor and delivery complicated by vasa previa, fetus 2 |
| childbirth | ICD-10-CM Diagnosis | O69.4XX3 | Labor and delivery complicated by vasa previa, fetus 3 |
| childbirth | ICD-10-CM Diagnosis | O69.4XX4 | Labor and delivery complicated by vasa previa, fetus 4 |
| childbirth | ICD-10-CM Diagnosis | O69.4XX5 | Labor and delivery complicated by vasa previa, fetus 5 |
| childbirth | ICD-10-CM Diagnosis | O69.4XX9 | Labor and delivery complicated by vasa previa, other fetus |
| childbirth | ICD-10-CM Diagnosis | O69.5 | Labor and delivery complicated by vascular lesion of cord |
| childbirth | ICD-10-CM Diagnosis | O69.5XX0 | Labor and delivery complicated by vascular lesion of cord, not applicable or unspecified |
| childbirth | ICD-10-CM Diagnosis | O69.5XX1 | Labor and delivery complicated by vascular lesion of cord, fetus 1 |
| childbirth | ICD-10-CM Diagnosis | O69.5XX2 | Labor and delivery complicated by vascular lesion of cord, fetus 2 |
| childbirth | ICD-10-CM Diagnosis | O69.5XX3 | Labor and delivery complicated by vascular lesion of cord, fetus 3 |
| childbirth | ICD-10-CM Diagnosis | O69.5XX4 | Labor and delivery complicated by vascular lesion of cord, fetus 4 |
| childbirth | ICD-10-CM Diagnosis | O69.5XX5 | Labor and delivery complicated by vascular lesion of cord, fetus 5 |
| childbirth | ICD-10-CM Diagnosis | O69.5XX9 | Labor and delivery complicated by vascular lesion of cord, other fetus |
| childbirth | ICD-10-CM Diagnosis | O69.8 | Labor and delivery complicated by other cord complications |
| childbirth | ICD-10-CM Diagnosis | O69.81 | Labor and delivery complicated by cord around neck, without compression |
| childbirth | ICD-10-CM Diagnosis | O69.81X0 | Labor and delivery complicated by cord around neck, without compression, not applicable or unspecified |
| childbirth | ICD-10-CM Diagnosis | O69.81X1 | Labor and delivery complicated by cord around neck, without compression, fetus 1 |
| childbirth | ICD-10-CM Diagnosis | O69.81X2 | Labor and delivery complicated by cord around neck, without compression, fetus 2 |
| childbirth | ICD-10-CM Diagnosis | O69.81X3 | Labor and delivery complicated by cord around neck, without compression, fetus 3 |
| childbirth | ICD-10-CM Diagnosis | O69.81X4 | Labor and delivery complicated by cord around neck, without compression, fetus 4 |
| childbirth | ICD-10-CM Diagnosis | O69.81X5 | Labor and delivery complicated by cord around neck, without compression, fetus 5 |
| childbirth | ICD-10-CM Diagnosis | O69.81X9 | Labor and delivery complicated by cord around neck, without compression, other fetus |
| childbirth | ICD-10-CM Diagnosis | O69.82 | Labor and delivery complicated by other cord entanglement, without compression |
| childbirth | ICD-10-CM Diagnosis | O69.82X0 | Labor and delivery complicated by other cord entanglement, without compression, not applicable or unspecified |
| childbirth | ICD-10-CM Diagnosis | O69.82X1 | Labor and delivery complicated by other cord entanglement, without compression, fetus 1 |
| childbirth | ICD-10-CM Diagnosis | O69.82X2 | Labor and delivery complicated by other cord entanglement, without compression, fetus 2 |
| childbirth | ICD-10-CM Diagnosis | O69.82X3 | Labor and delivery complicated by other cord entanglement, without compression, fetus 3 |
| childbirth | ICD-10-CM Diagnosis | O69.82X4 | Labor and delivery complicated by other cord entanglement, without compression, fetus 4 |
| childbirth | ICD-10-CM Diagnosis | O69.82X5 | Labor and delivery complicated by other cord entanglement, without compression, fetus 5 |
| childbirth | ICD-10-CM Diagnosis | O69.82X9 | Labor and delivery complicated by other cord entanglement, without compression, other fetus |
| childbirth | ICD-10-CM Diagnosis | O69.89 | Labor and delivery complicated by other cord complications |
| childbirth | ICD-10-CM Diagnosis | O69.89X0 | Labor and delivery complicated by other cord complications, not applicable or unspecified |
| childbirth | ICD-10-CM Diagnosis | O69.89X1 | Labor and delivery complicated by other cord complications, fetus 1 |
| childbirth | ICD-10-CM Diagnosis | O69.89X2 | Labor and delivery complicated by other cord complications, fetus 2 |
| childbirth | ICD-10-CM Diagnosis | O69.89X3 | Labor and delivery complicated by other cord complications, fetus 3 |
| childbirth | ICD-10-CM Diagnosis | O69.89X4 | Labor and delivery complicated by other cord complications, fetus 4 |
| childbirth | ICD-10-CM Diagnosis | O69.89X5 | Labor and delivery complicated by other cord complications, fetus 5 |
| childbirth | ICD-10-CM Diagnosis | O69.89X9 | Labor and delivery complicated by other cord complications, other fetus |
| childbirth | ICD-10-CM Diagnosis | O69.8XX0 | Labor and delivery complicated by other cord complications, not applicable or unspecified |
| childbirth | ICD-10-CM Diagnosis | O69.8XX1 | Labor and delivery complicated by other cord complications, fetus 1 |
| childbirth | ICD-10-CM Diagnosis | O69.8XX2 | Labor and delivery complicated by other cord complications, fetus 2 |
| childbirth | ICD-10-CM Diagnosis | O69.8XX3 | Labor and delivery complicated by other cord complications, fetus 3 |
| childbirth | ICD-10-CM Diagnosis | O69.8XX4 | Labor and delivery complicated by other cord complications, fetus 4 |
| childbirth | ICD-10-CM Diagnosis | O69.8XX5 | Labor and delivery complicated by other cord complications, fetus 5 |
| childbirth | ICD-10-CM Diagnosis | O69.8XX9 | Labor and delivery complicated by other cord complications, other fetus |
| childbirth | ICD-10-CM Diagnosis | O69.9 | Labor and delivery complicated by cord complication, unspecified |
| childbirth | ICD-10-CM Diagnosis | O69.9XX0 | Labor and delivery complicated by cord complication, unspecified, not applicable or unspecified |
| childbirth | ICD-10-CM Diagnosis | O69.9XX1 | Labor and delivery complicated by cord complication, unspecified, fetus 1 |
| childbirth | ICD-10-CM Diagnosis | O69.9XX2 | Labor and delivery complicated by cord complication, unspecified, fetus 2 |
| childbirth | ICD-10-CM Diagnosis | O69.9XX3 | Labor and delivery complicated by cord complication, unspecified, fetus 3 |
| childbirth | ICD-10-CM Diagnosis | O69.9XX4 | Labor and delivery complicated by cord complication, unspecified, fetus 4 |
| childbirth | ICD-10-CM Diagnosis | O69.9XX5 | Labor and delivery complicated by cord complication, unspecified, fetus 5 |
| childbirth | ICD-10-CM Diagnosis | O69.9XX9 | Labor and delivery complicated by cord complication, unspecified, other fetus |
| childbirth | ICD-10-CM Diagnosis | O70 | Perineal laceration during delivery |
| childbirth | ICD-10-CM Diagnosis | O70.0 | First degree perineal laceration during delivery |
| childbirth | ICD-10-CM Diagnosis | O70.1 | Second degree perineal laceration during delivery |
| childbirth | ICD-10-CM Diagnosis | O70.2 | Third degree perineal laceration during delivery |
| childbirth | ICD-10-CM Diagnosis | O70.20 | Third degree perineal laceration during delivery, unspecified |
| childbirth | ICD-10-CM Diagnosis | O70.21 | Third degree perineal laceration during delivery, IIIa |
| childbirth | ICD-10-CM Diagnosis | O70.22 | Third degree perineal laceration during delivery, IIIb |
| childbirth | ICD-10-CM Diagnosis | O70.23 | Third degree perineal laceration during delivery, IIIc |
| childbirth | ICD-10-CM Diagnosis | O70.3 | Fourth degree perineal laceration during delivery |
| childbirth | ICD-10-CM Diagnosis | O70.4 | Anal sphincter tear complicating delivery, not associated with third degree laceration |
| childbirth | ICD-10-CM Diagnosis | O70.9 | Perineal laceration during delivery, unspecified |
| childbirth | ICD-10-CM Diagnosis | O71 | Other obstetric trauma |
| childbirth | ICD-10-CM Diagnosis | O71.0 | Rupture of uterus (spontaneous) before onset of labor |
| childbirth | ICD-10-CM Diagnosis | O71.00 | Rupture of uterus before onset of labor, unspecified trimester |
| childbirth | ICD-10-CM Diagnosis | O71.02 | Rupture of uterus before onset of labor, second trimester |
| childbirth | ICD-10-CM Diagnosis | O71.03 | Rupture of uterus before onset of labor, third trimester |
| childbirth | ICD-10-CM Diagnosis | O71.1 | Rupture of uterus during labor |
| childbirth | ICD-10-CM Diagnosis | O71.2 | Postpartum inversion of uterus |
| childbirth | ICD-10-CM Diagnosis | O71.3 | Obstetric laceration of cervix |
| childbirth | ICD-10-CM Diagnosis | O71.4 | Obstetric high vaginal laceration alone |
| childbirth | ICD-10-CM Diagnosis | O71.5 | Other obstetric injury to pelvic organs |
| childbirth | ICD-10-CM Diagnosis | O71.6 | Obstetric damage to pelvic joints and ligaments |
| childbirth | ICD-10-CM Diagnosis | O71.7 | Obstetric hematoma of pelvis |
| childbirth | ICD-10-CM Diagnosis | O71.8 | Other specified obstetric trauma |
| childbirth | ICD-10-CM Diagnosis | O71.81 | Laceration of uterus, not elsewhere classified |
| childbirth | ICD-10-CM Diagnosis | O71.82 | Other specified trauma to perineum and vulva |
| childbirth | ICD-10-CM Diagnosis | O71.89 | Other specified obstetric trauma |
| childbirth | ICD-10-CM Diagnosis | O71.9 | Obstetric trauma, unspecified |
| childbirth | ICD-10-CM Diagnosis | O72.0 | Third-stage hemorrhage |
| childbirth | ICD-10-CM Diagnosis | O72.1 | Other immediate postpartum hemorrhage |
| childbirth | ICD-10-CM Diagnosis | O74 | Complications of anesthesia during labor and delivery |
| childbirth | ICD-10-CM Diagnosis | O74.0 | Aspiration pneumonitis due to anesthesia during labor and delivery |
| childbirth | ICD-10-CM Diagnosis | O74.1 | Other pulmonary complications of anesthesia during labor and delivery |
| childbirth | ICD-10-CM Diagnosis | O74.2 | Cardiac complications of anesthesia during labor and delivery |
| childbirth | ICD-10-CM Diagnosis | O74.3 | Central nervous system complications of anesthesia during labor and delivery |
| childbirth | ICD-10-CM Diagnosis | O74.4 | Toxic reaction to local anesthesia during labor and delivery |
| childbirth | ICD-10-CM Diagnosis | O74.5 | Spinal and epidural anesthesia-induced headache during labor and delivery |
| childbirth | ICD-10-CM Diagnosis | O74.6 | Other complications of spinal and epidural anesthesia during labor and delivery |
| childbirth | ICD-10-CM Diagnosis | O74.7 | Failed or difficult intubation for anesthesia during labor and delivery |
| childbirth | ICD-10-CM Diagnosis | O74.8 | Other complications of anesthesia during labor and delivery |
| childbirth | ICD-10-CM Diagnosis | O74.9 | Complication of anesthesia during labor and delivery, unspecified |
| childbirth | ICD-10-CM Diagnosis | O75 | Other complications of labor and delivery, not elsewhere classified |
| childbirth | ICD-10-CM Diagnosis | O75.0 | Maternal distress during labor and delivery |
| childbirth | ICD-10-CM Diagnosis | O75.1 | Shock during or following labor and delivery |
| childbirth | ICD-10-CM Diagnosis | O75.2 | Pyrexia during labor, not elsewhere classified |
| childbirth | ICD-10-CM Diagnosis | O75.3 | Other infection during labor |
| childbirth | ICD-10-CM Diagnosis | O75.4 | Other complications of obstetric surgery and procedures |
| childbirth | ICD-10-CM Diagnosis | O75.5 | Delayed delivery after artificial rupture of membranes |
| childbirth | ICD-10-CM Diagnosis | O75.6 | Delayed delivery after spontaneous or unspecified rupture of membranes |
| childbirth | ICD-10-CM Diagnosis | O75.7 | Vaginal delivery following previous caesarean section |
| childbirth | ICD-10-CM Diagnosis | O75.8 | Other specified complications of labor and delivery |
| childbirth | ICD-10-CM Diagnosis | O75.81 | Maternal exhaustion complicating labor and delivery |
| childbirth | ICD-10-CM Diagnosis | O75.82 | Onset (spontaneous) of labor after 37 completed weeks of gestation but before 39 completed weeks gestation, with delivery by (planned) cesarean section |
| childbirth | ICD-10-CM Diagnosis | O75.89 | Other specified complications of labor and delivery |
| childbirth | ICD-10-CM Diagnosis | O75.9 | Complication of labor and delivery, unspecified |
| childbirth | ICD-10-CM Diagnosis | O76 | Abnormality in fetal heart rate and rhythm complicating labor and delivery |
| childbirth | ICD-10-CM Diagnosis | O77 | Other fetal stress complicating labor and delivery |
| childbirth | ICD-10-CM Diagnosis | O77.0 | Labor and delivery complicated by meconium in amniotic fluid |
| childbirth | ICD-10-CM Diagnosis | O77.1 | Fetal stress in labor or delivery due to drug administration |
| childbirth | ICD-10-CM Diagnosis | O77.8 | Labor and delivery complicated by other evidence of fetal stress |
| childbirth | ICD-10-CM Diagnosis | O77.9 | Labor and delivery complicated by fetal stress, unspecified |
| childbirth | ICD-10-CM Diagnosis | O80 | Encounter for full-term uncomplicated delivery |
| childbirth | ICD-10-CM Diagnosis | O82 | Encounter for cesarean delivery without indication |
| childbirth | ICD-10-CM Diagnosis | O88.02 | Air embolism in childbirth |
| childbirth | ICD-10-CM Diagnosis | O88.12 | Amniotic fluid embolism in childbirth |
| childbirth | ICD-10-CM Diagnosis | O88.22 | Thromboembolism in childbirth |
| childbirth | ICD-10-CM Diagnosis | O88.32 | Pyemic and septic embolism in childbirth |
| childbirth | ICD-10-CM Diagnosis | O88.82 | Other embolism in childbirth |
| childbirth | ICD-10-CM Diagnosis | O98.02 | Tuberculosis complicating childbirth |
| childbirth | ICD-10-CM Diagnosis | O98.12 | Syphilis complicating childbirth |
| childbirth | ICD-10-CM Diagnosis | O98.22 | Gonorrhea complicating childbirth |
| childbirth | ICD-10-CM Diagnosis | O98.32 | Other infections with a predominantly sexual mode of transmission complicating childbirth |
| childbirth | ICD-10-CM Diagnosis | O98.42 | Viral hepatitis complicating childbirth |
| childbirth | ICD-10-CM Diagnosis | O98.52 | Other viral diseases complicating childbirth |
| childbirth | ICD-10-CM Diagnosis | O98.62 | Protozoal diseases complicating childbirth |
| childbirth | ICD-10-CM Diagnosis | O98.72 | Human immunodeficiency virus [HIV] disease complicating childbirth |
| childbirth | ICD-10-CM Diagnosis | O98.82 | Other maternal infectious and parasitic diseases complicating childbirth |
| childbirth | ICD-10-CM Diagnosis | O98.92 | Unspecified maternal infectious and parasitic disease complicating childbirth |
| childbirth | ICD-10-CM Diagnosis | O99.02 | Anemia complicating childbirth |
| childbirth | ICD-10-CM Diagnosis | O99.12 | Other diseases of the blood and blood-forming organs and certain disorders involving the immune mechanism complicating childbirth |
| childbirth | ICD-10-CM Diagnosis | O99.214 | Obesity complicating childbirth |
| childbirth | ICD-10-CM Diagnosis | O99.284 | Endocrine, nutritional and metabolic diseases complicating childbirth |
| childbirth | ICD-10-CM Diagnosis | O99.314 | Alcohol use complicating childbirth |
| childbirth | ICD-10-CM Diagnosis | O99.324 | Drug use complicating childbirth |
| childbirth | ICD-10-CM Diagnosis | O99.334 | Smoking (tobacco) complicating childbirth |
| childbirth | ICD-10-CM Diagnosis | O99.344 | Other mental disorders complicating childbirth |
| childbirth | ICD-10-CM Diagnosis | O99.354 | Diseases of the nervous system complicating childbirth |
| childbirth | ICD-10-CM Diagnosis | O99.42 | Diseases of the circulatory system complicating childbirth |
| childbirth | ICD-10-CM Diagnosis | O99.52 | Diseases of the respiratory system complicating childbirth |
| childbirth | ICD-10-CM Diagnosis | O99.62 | Diseases of the digestive system complicating childbirth |
| childbirth | ICD-10-CM Diagnosis | O99.72 | Diseases of the skin and subcutaneous tissue complicating childbirth |
| childbirth | ICD-10-CM Diagnosis | O99.814 | Abnormal glucose complicating childbirth |
| childbirth | ICD-10-CM Diagnosis | O99.824 | Streptococcus B carrier state complicating childbirth |
| childbirth | ICD-10-CM Diagnosis | O99.834 | Other infection carrier state complicating childbirth |
| childbirth | ICD-10-CM Diagnosis | O99.844 | Bariatric surgery status complicating childbirth |
| childbirth | ICD-10-CM Diagnosis | O9A.12 | Malignant neoplasm complicating childbirth |
| childbirth | ICD-10-CM Diagnosis | O9A.22 | Injury, poisoning and certain other consequences of external causes complicating childbirth |
| childbirth | ICD-10-CM Diagnosis | O9A.32 | Physical abuse complicating childbirth |
| childbirth | ICD-10-CM Diagnosis | O9A.42 | Sexual abuse complicating childbirth |
| childbirth | ICD-10-CM Diagnosis | O9A.52 | Psychological abuse complicating childbirth |
| childbirth | ICD-10-CM Diagnosis | Z37 | Outcome of delivery |
| childbirth | ICD-10-CM Diagnosis | Z37.0 | Single live birth |
| childbirth | ICD-10-CM Diagnosis | Z37.1 | Single stillbirth |
| childbirth | ICD-10-CM Diagnosis | Z37.2 | Twins, both liveborn |
| childbirth | ICD-10-CM Diagnosis | Z37.3 | Twins, one liveborn and one stillborn |
| childbirth | ICD-10-CM Diagnosis | Z37.4 | Twins, both stillborn |
| childbirth | ICD-10-CM Diagnosis | Z37.5 | Other multiple births, all liveborn |
| childbirth | ICD-10-CM Diagnosis | Z37.50 | Multiple births, unspecified, all liveborn |
| childbirth | ICD-10-CM Diagnosis | Z37.51 | Triplets, all liveborn |
| childbirth | ICD-10-CM Diagnosis | Z37.52 | Quadruplets, all liveborn |
| childbirth | ICD-10-CM Diagnosis | Z37.53 | Quintuplets, all liveborn |
| childbirth | ICD-10-CM Diagnosis | Z37.54 | Sextuplets, all liveborn |
| childbirth | ICD-10-CM Diagnosis | Z37.59 | Other multiple births, all liveborn |
| childbirth | ICD-10-CM Diagnosis | Z37.6 | Other multiple births, some liveborn |
| childbirth | ICD-10-CM Diagnosis | Z37.60 | Multiple births, unspecified, some liveborn |
| childbirth | ICD-10-CM Diagnosis | Z37.61 | Triplets, some liveborn |
| childbirth | ICD-10-CM Diagnosis | Z37.62 | Quadruplets, some liveborn |
| childbirth | ICD-10-CM Diagnosis | Z37.63 | Quintuplets, some liveborn |
| childbirth | ICD-10-CM Diagnosis | Z37.64 | Sextuplets, some liveborn |
| childbirth | ICD-10-CM Diagnosis | Z37.69 | Other multiple births, some liveborn |
| childbirth | ICD-10-CM Diagnosis | Z37.7 | Other multiple births, all stillborn |
| childbirth | ICD-10-CM Diagnosis | Z37.9 | Outcome of delivery, unspecified |

CPT, Current Procedural Terminology; ICD-10-CM, International Classification of Diseases, Tenth Revision, Clinical Modification.
