# Supplementary material for: Real-world treatment patterns and clinical outcomes for inpatients with COVID-19 in the US from September 2020 to February 2021
Source: PLoS One. 2021 Dec 28;16(12):e0261707. doi: 10.1371/journal.pone.0261707 (PMC8714107; doi:10.1371/journal.pone.0261707)
Supplement: S2 Table — a) Cardiac disease diagnosis ICD-9-CM, International Classification of Diseases, Ninth Revision, Clinical Modification; ICD-10-CM, International Classification of Diseases, Tenth Revision, Clinical Modification. b) Respiratory disease diagnosis. ARDS, acute respiratory distress syndrome; International Classification of Diseases, Tenth Revision, Clinical Modification. c) Diabetes mellitus diagnosis. International Classification of Diseases, Tenth Revision, Clinical Modification. d) Kidney disease diagnosis. International Classification of Diseases, Tenth Revision, Clinical Modification. e) Kidney disease procedures. CPT, Current Procedural Terminology; HCPCS, Healthcare Common Procedure Coding System; ICD-10-PCS, International Classification of Diseases, Tenth Revision, Procedure Coding System. f) Venous thromboembolism (VTE) diagnosis. ICD-9-CM, International Classification of Diseases, Ninth Revision, Clinical Modification; ICD-10-CM, International Classification of Diseases, Tenth Revision, Clinical Modification. (DOCX) [file pone.0261707.s002.docx]

## Table S2. Diagnosis and procedure codes used to identify patient comorbidities

a) Cardiac disease diagnosis

| **Criterion Name** | **Code Type** | **Code** | **Code Description** | **Full description** |
| --- | --- | --- | --- | --- |
| Acute heart failure | ICD-10-CM Diagnosis | I11.0 | Acute heart failure | Hypertensive heart disease with heart failure |
| Acute heart failure | ICD-10-CM Diagnosis | I50.1 | Acute heart failure | Left ventricular failure, unspecified |
| Acute heart failure | ICD-10-CM Diagnosis | I50.2 | Acute heart failure | Systolic (congestive) heart failure |
| Acute heart failure | ICD-10-CM Diagnosis | I50.20 | Acute heart failure | Unspecified systolic (congestive) heart failure |
| Acute heart failure | ICD-10-CM Diagnosis | I50.21 | Acute heart failure | Acute systolic (congestive) heart failure |
| Acute heart failure | ICD-10-CM Diagnosis | I50.23 | Acute heart failure | Acute on chronic systolic (congestive) heart failure |
| Acute heart failure | ICD-10-CM Diagnosis | I50.30 | Acute heart failure | Unspecified diastolic (congestive) heart failure |
| Acute heart failure | ICD-10-CM Diagnosis | I50.31 | Acute heart failure | Acute diastolic (congestive) heart failure |
| Acute heart failure | ICD-10-CM Diagnosis | I50.33 | Acute heart failure | Acute on chronic diastolic (congestive) heart failure |
| Acute heart failure | ICD-10-CM Diagnosis | I50.40 | Acute heart failure | Unspecified combined systolic (congestive) and diastolic (congestive) heart failure |
| Acute heart failure | ICD-10-CM Diagnosis | I50.41 | Acute heart failure | Acute combined systolic (congestive) and diastolic (congestive) heart failure |
| Acute heart failure | ICD-10-CM Diagnosis | I50.43 | Acute heart failure | Acute on chronic combined systolic (congestive) and diastolic (congestive) heart failure |
| Acute heart failure | ICD-10-CM Diagnosis | I50.810 | Acute heart failure | Right heart failure, unspecified |
| Acute heart failure | ICD-10-CM Diagnosis | I50.811 | Acute heart failure | Acute right heart failure |
| Acute heart failure | ICD-10-CM Diagnosis | I50.813 |  |  |
| Acute heart failure | ICD-10-CM Diagnosis | I50.814 |  |  |
| Acute heart failure | ICD-10-CM Diagnosis | I50.82 | Acute heart failure | Biventricular heart failure |
| Acute heart failure | ICD-10-CM Diagnosis | I50.83 |  |  |
| Acute heart failure | ICD-10-CM Diagnosis | I50.84 | Acute heart failure | End stage heart failure |
| Acute heart failure | ICD-10-CM Diagnosis | I50.89 | Acute heart failure | Other heart failure |
| Acute heart failure | ICD-10-CM Diagnosis | I50.9 | Acute heart failure | Heart failure, unspecified |
| Cardiovascular disease | ICD-9-CM Diagnosis | 398.91 | Congestive heart failure | Rheumatic heart failure (congestive) |
| Cardiovascular disease | ICD-9-CM Diagnosis | 402.01 | Congestive heart failure | Malignant hypertensive heart disease with heart failure |
| Cardiovascular disease | ICD-9-CM Diagnosis | 402.11 | Congestive heart failure | Benign hypertensive heart disease with heart failure |
| Cardiovascular disease | ICD-9-CM Diagnosis | 402.91 | Congestive heart failure | Unspecified hypertensive heart disease with heart failure |
| Cardiovascular disease | ICD-9-CM Diagnosis | 404.01 | Congestive heart failure | Hypertensive heart and chronic kidney disease, malignant, with heart failure and with chronic kidney disease stage I through stage IV, or unspecified |
| Cardiovascular disease | ICD-9-CM Diagnosis | 404.03 | Congestive heart failure | Hypertensive heart and chronic kidney disease, malignant, with heart failure and with chronic kidney disease stage V or end stage renal disease |
| Cardiovascular disease | ICD-9-CM Diagnosis | 404.11 | Congestive heart failure | Hypertensive heart and chronic kidney disease, benign, with heart failure and with chronic kidney disease stage I through stage IV, or unspecified |
| Cardiovascular disease | ICD-9-CM Diagnosis | 404.9 | Congestive heart failure | Hypertensive heart and renal disease, unspecified |
| Cardiovascular disease | ICD-9-CM Diagnosis | 404.90 | Congestive heart failure | Hypertensive heart and chronic kidney disease, unspecified, without heart failure and with chronic kidney disease stage I through stage IV, or unspecified |
| Cardiovascular disease | ICD-9-CM Diagnosis | 404.91 | Congestive heart failure | Hypertensive heart and chronic kidney disease, unspecified, with heart failure and with chronic kidney disease stage I through stage IV, or unspecified |
| Cardiovascular disease | ICD-9-CM Diagnosis | 404.93 | Congestive heart failure | Hypertensive heart and chronic kidney disease, unspecified, with heart failure and chronic kidney disease stage V or end stage renal disease |
| Cardiovascular disease | ICD-9-CM Diagnosis | 428.0 | Congestive heart failure | Congestive heart failure, unspecified |
| Cardiovascular disease | ICD-10-CM Diagnosis | I11.0 | Congestive heart failure | Hypertensive heart disease with heart failure |
| Cardiovascular disease | ICD-10-CM Diagnosis | I13 | Congestive heart failure | Hypertensive heart and chronic kidney disease |
| Cardiovascular disease | ICD-10-CM Diagnosis | I13.0 | Congestive heart failure | Hypertensive heart and chronic kidney disease with heart failure and stage 1 through stage 4 chronic kidney disease, or unspecified chronic kidney disease |
| Cardiovascular disease | ICD-10-CM Diagnosis | I13.1 | Congestive heart failure | Hypertensive heart and chronic kidney disease without heart failure |
| Cardiovascular disease | ICD-10-CM Diagnosis | I13.10 | Congestive heart failure | Hypertensive heart and chronic kidney disease without heart failure, with stage 1 through stage 4 chronic kidney disease, or unspecified chronic kidney disease |
| Cardiovascular disease | ICD-10-CM Diagnosis | I13.11 | Congestive heart failure | Hypertensive heart and chronic kidney disease without heart failure, with stage 5 chronic kidney disease, or end stage renal disease |
| Cardiovascular disease | ICD-10-CM Diagnosis | I13.2 | Congestive heart failure | Hypertensive heart and chronic kidney disease with heart failure and with stage 5 chronic kidney disease, or end stage renal disease |
| Cardiovascular disease | ICD-10-CM Diagnosis | I50.2 | Congestive heart failure | Systolic (congestive) heart failure |
| Cardiovascular disease | ICD-10-CM Diagnosis | I50.20 | Congestive heart failure | Unspecified systolic (congestive) heart failure |
| Cardiovascular disease | ICD-10-CM Diagnosis | I50.22 | Congestive heart failure | Chronic systolic (congestive) heart failure |
| Cardiovascular disease | ICD-10-CM Diagnosis | I50.23 | Congestive heart failure | Acute on chronic systolic (congestive) heart failure |
| Cardiovascular disease | ICD-10-CM Diagnosis | I50.3 | Congestive heart failure | Diastolic (congestive) heart failure |
| Cardiovascular disease | ICD-10-CM Diagnosis | I50.30 | Congestive heart failure | Unspecified diastolic (congestive) heart failure |
| Cardiovascular disease | ICD-10-CM Diagnosis | I50.32 | Congestive heart failure | Chronic diastolic (congestive) heart failure |
| Cardiovascular disease | ICD-10-CM Diagnosis | I50.33 | Congestive heart failure | Acute on chronic diastolic (congestive) heart failure |
| Cardiovascular disease | ICD-10-CM Diagnosis | I50.4 | Congestive heart failure | Combined systolic (congestive) and diastolic (congestive) heart failure |
| Cardiovascular disease | ICD-10-CM Diagnosis | I50.40 | Congestive heart failure | Unspecified combined systolic (congestive) and diastolic (congestive) heart failure |
| Cardiovascular disease | ICD-10-CM Diagnosis | I50.42 | Congestive heart failure | Chronic combined systolic (congestive) and diastolic (congestive) heart failure |
| Cardiovascular disease | ICD-10-CM Diagnosis | I50.43 | Congestive heart failure | Acute on chronic combined systolic (congestive) and diastolic (congestive) heart failure |
| Cardiovascular disease | ICD-10-CM Diagnosis | I50.813 | Congestive heart failure | Acute on chronic right heart failure |
| Cardiovascular disease | ICD-10-CM Diagnosis | I50.82 | Congestive heart failure | Biventricular heart failure |

ICD-9-CM, International Classification of Diseases, Ninth Revision, Clinical Modification; ICD-10-CM, International Classification of Diseases, Tenth Revision, Clinical Modification.

b) Respiratory disease diagnosis

| **Criterion Name** | **Code Type** | **Code** | **Code Description** | **Full Description** |
| --- | --- | --- | --- | --- |
| ARDS | ICD-10-CM diagnosis | J80 | ARDS | Acute respiratory distress syndrome |
| Respiratory failure | ICD-10-CM diagnosis | J96.0 | Respiratory failure | Acute respiratory failure |
| Respiratory failure | ICD-10-CM diagnosis | J96.01 | Respiratory failure | Acute respiratory failure with hypoxia |
| Respiratory failure | ICD-10-CM diagnosis | J96.02 | Respiratory failure | Acute respiratory failure with hypercapnia |
| Respiratory failure | ICD-10-CM diagnosis | J96.20 | Respiratory failure | Acute and chronic respiratory failure, unspecified whether with hypoxia or hypercapnia |
| Respiratory failure | ICD-10-CM diagnosis | J96.21 | Respiratory failure | Acute and chronic respiratory failure with hypoxia |
| Respiratory failure | ICD-10-CM diagnosis | J96.22 | Respiratory failure | Acute and chronic respiratory failure with hypercapnia |

ARDS, acute respiratory distress syndrome; International Classification of Diseases, Tenth Revision, Clinical Modification.

c) Diabetes mellitus diagnosis

| **Criterion Name** | **Code Type** | **Code** | **Code Description** | **Full description** |
| --- | --- | --- | --- | --- |
| Diabetes mellitus | ICD-10-CM Diagnosis | E08 | Diabetes (type unknown) | Diabetes mellitus due to underlying condition |
| Diabetes mellitus | ICD-10-CM Diagnosis | E08.0 | Diabetes (type unknown) | Diabetes mellitus due to underlying condition with hyperosmolarity |
| Diabetes mellitus | ICD-10-CM Diagnosis | E08.00 | Diabetes (type unknown) | Diabetes mellitus due to underlying condition with hyperosmolarity without nonketotic hyperglycemic-hyperosmolar coma (NKHHC) |
| Diabetes mellitus | ICD-10-CM Diagnosis | E08.01 | Diabetes (type unknown) | Diabetes mellitus due to underlying condition with hyperosmolarity with coma |
| Diabetes mellitus | ICD-10-CM Diagnosis | E08.2 | Diabetes (type unknown) | Diabetes mellitus due to underlying condition with kidney complications |
| Diabetes mellitus | ICD-10-CM Diagnosis | E08.21 | Diabetes (type unknown) | Diabetes mellitus due to underlying condition with diabetic nephropathy |
| Diabetes mellitus | ICD-10-CM Diagnosis | E08.22 | Diabetes (type unknown) | Diabetes mellitus due to underlying condition with diabetic chronic kidney disease |
| Diabetes mellitus | ICD-10-CM Diagnosis | E08.29 | Diabetes (type unknown) | Diabetes mellitus due to underlying condition with other diabetic kidney complication |
| Diabetes mellitus | ICD-10-CM Diagnosis | E08.3 | Diabetes (type unknown) | Diabetes mellitus due to underlying condition with ophthalmic complications |
| Diabetes mellitus | ICD-10-CM Diagnosis | E08.31 | Diabetes (type unknown) | Diabetes mellitus due to underlying condition with unspecified diabetic retinopathy |
| Diabetes mellitus | ICD-10-CM Diagnosis | E08.311 | Diabetes (type unknown) | Diabetes mellitus due to underlying condition with unspecified diabetic retinopathy with macular edema |
| Diabetes mellitus | ICD-10-CM Diagnosis | E08.319 | Diabetes (type unknown) | Diabetes mellitus due to underlying condition with unspecified diabetic retinopathy without macular edema |
| Diabetes mellitus | ICD-10-CM Diagnosis | E08.32 | Diabetes (type unknown) | Diabetes mellitus due to underlying condition with mild nonproliferative diabetic retinopathy |
| Diabetes mellitus | ICD-10-CM Diagnosis | E08.321 | Diabetes (type unknown) | Diabetes mellitus due to underlying condition with mild nonproliferative diabetic retinopathy with macular edema |
| Diabetes mellitus | ICD-10-CM Diagnosis | E08.3211 | Diabetes (type unknown) | Diabetes mellitus due to underlying condition with mild nonproliferative diabetic retinopathy with macular edema, right eye |
| Diabetes mellitus | ICD-10-CM Diagnosis | E08.3212 | Diabetes (type unknown) | Diabetes mellitus due to underlying condition with mild nonproliferative diabetic retinopathy with macular edema, left eye |
| Diabetes mellitus | ICD-10-CM Diagnosis | E08.3213 | Diabetes (type unknown) | Diabetes mellitus due to underlying condition with mild nonproliferative diabetic retinopathy with macular edema, bilateral |
| Diabetes mellitus | ICD-10-CM Diagnosis | E08.3219 | Diabetes (type unknown) | Diabetes mellitus due to underlying condition with mild nonproliferative diabetic retinopathy with macular edema, unspecified eye |
| Diabetes mellitus | ICD-10-CM Diagnosis | E08.329 | Diabetes (type unknown) | Diabetes mellitus due to underlying condition with mild nonproliferative diabetic retinopathy without macular edema |
| Diabetes mellitus | ICD-10-CM Diagnosis | E08.3291 | Diabetes (type unknown) | Diabetes mellitus due to underlying condition with mild nonproliferative diabetic retinopathy without macular edema, right eye |
| Diabetes mellitus | ICD-10-CM Diagnosis | E08.3292 | Diabetes (type unknown) | Diabetes mellitus due to underlying condition with mild nonproliferative diabetic retinopathy without macular edema, left eye |
| Diabetes mellitus | ICD-10-CM Diagnosis | E08.3293 | Diabetes (type unknown) | Diabetes mellitus due to underlying condition with mild nonproliferative diabetic retinopathy without macular edema, bilateral |
| Diabetes mellitus | ICD-10-CM Diagnosis | E08.3299 | Diabetes (type unknown) | Diabetes mellitus due to underlying condition with mild nonproliferative diabetic retinopathy without macular edema, unspecified eye |
| Diabetes mellitus | ICD-10-CM Diagnosis | E08.3311 | Diabetes (type unknown) | Diabetes mellitus due to underlying condition with moderate nonproliferative diabetic retinopathy with macular edema, right eye |
| Diabetes mellitus | ICD-10-CM Diagnosis | E08.3312 | Diabetes (type unknown) | Diabetes mellitus due to underlying condition with moderate nonproliferative diabetic retinopathy with macular edema, left eye |
| Diabetes mellitus | ICD-10-CM Diagnosis | E08.3313 | Diabetes (type unknown) | Diabetes mellitus due to underlying condition with moderate nonproliferative diabetic retinopathy with macular edema, bilateral |
| Diabetes mellitus | ICD-10-CM Diagnosis | E08.3391 | Diabetes (type unknown) | Diabetes mellitus due to underlying condition with moderate nonproliferative diabetic retinopathy without macular edema, right eye |
| Diabetes mellitus | ICD-10-CM Diagnosis | E08.3392 | Diabetes (type unknown) | Diabetes mellitus due to underlying condition with moderate nonproliferative diabetic retinopathy without macular edema, left eye |
| Diabetes mellitus | ICD-10-CM Diagnosis | E08.3393 | Diabetes (type unknown) | Diabetes mellitus due to underlying condition with moderate nonproliferative diabetic retinopathy without macular edema, bilateral |
| Diabetes mellitus | ICD-10-CM Diagnosis | E08.34 | Diabetes (type unknown) | Diabetes mellitus due to underlying condition with severe nonproliferative diabetic retinopathy |
| Diabetes mellitus | ICD-10-CM Diagnosis | E08.341 | Diabetes (type unknown) | Diabetes mellitus due to underlying condition with severe nonproliferative diabetic retinopathy with macular edema |
| Diabetes mellitus | ICD-10-CM Diagnosis | E08.3411 | Diabetes (type unknown) | Diabetes mellitus due to underlying condition with severe nonproliferative diabetic retinopathy with macular edema, right eye |
| Diabetes mellitus | ICD-10-CM Diagnosis | E08.3412 | Diabetes (type unknown) | Diabetes mellitus due to underlying condition with severe nonproliferative diabetic retinopathy with macular edema, left eye |
| Diabetes mellitus | ICD-10-CM Diagnosis | E08.3413 | Diabetes (type unknown) | Diabetes mellitus due to underlying condition with severe nonproliferative diabetic retinopathy with macular edema, bilateral |
| Diabetes mellitus | ICD-10-CM Diagnosis | E08.3419 | Diabetes (type unknown) | Diabetes mellitus due to underlying condition with severe nonproliferative diabetic retinopathy with macular edema, unspecified eye |
| Diabetes mellitus | ICD-10-CM Diagnosis | E08.349 | Diabetes (type unknown) | Diabetes mellitus due to underlying condition with severe nonproliferative diabetic retinopathy without macular edema |
| Diabetes mellitus | ICD-10-CM Diagnosis | E08.3491 | Diabetes (type unknown) | Diabetes mellitus due to underlying condition with severe nonproliferative diabetic retinopathy without macular edema, right eye |
| Diabetes mellitus | ICD-10-CM Diagnosis | E08.3492 | Diabetes (type unknown) | Diabetes mellitus due to underlying condition with severe nonproliferative diabetic retinopathy without macular edema, left eye |
| Diabetes mellitus | ICD-10-CM Diagnosis | E08.3493 | Diabetes (type unknown) | Diabetes mellitus due to underlying condition with severe nonproliferative diabetic retinopathy without macular edema, bilateral |
| Diabetes mellitus | ICD-10-CM Diagnosis | E08.3499 | Diabetes (type unknown) | Diabetes mellitus due to underlying condition with severe nonproliferative diabetic retinopathy without macular edema, unspecified eye |
| Diabetes mellitus | ICD-10-CM Diagnosis | E08.35 | Diabetes (type unknown) | Diabetes mellitus due to underlying condition with proliferative diabetic retinopathy |
| Diabetes mellitus | ICD-10-CM Diagnosis | E08.351 | Diabetes (type unknown) | Diabetes mellitus due to underlying condition with proliferative diabetic retinopathy with macular edema |
| Diabetes mellitus | ICD-10-CM Diagnosis | E08.3511 | Diabetes (type unknown) | Diabetes mellitus due to underlying condition with proliferative diabetic retinopathy with macular edema, right eye |
| Diabetes mellitus | ICD-10-CM Diagnosis | E08.3512 | Diabetes (type unknown) | Diabetes mellitus due to underlying condition with proliferative diabetic retinopathy with macular edema, left eye |
| Diabetes mellitus | ICD-10-CM Diagnosis | E08.3513 | Diabetes (type unknown) | Diabetes mellitus due to underlying condition with proliferative diabetic retinopathy with macular edema, bilateral |
| Diabetes mellitus | ICD-10-CM Diagnosis | E08.3519 | Diabetes (type unknown) | Diabetes mellitus due to underlying condition with proliferative diabetic retinopathy with macular edema, unspecified eye |
| Diabetes mellitus | ICD-10-CM Diagnosis | E08.352 | Diabetes (type unknown) | Diabetes mellitus due to underlying condition with proliferative diabetic retinopathy with traction retinal detachment involving the macula |
| Diabetes mellitus | ICD-10-CM Diagnosis | E08.3521 | Diabetes (type unknown) | Diabetes mellitus due to underlying condition with proliferative diabetic retinopathy with traction retinal detachment involving the macula, right eye |
| Diabetes mellitus | ICD-10-CM Diagnosis | E08.3522 | Diabetes (type unknown) | Diabetes mellitus due to underlying condition with proliferative diabetic retinopathy with traction retinal detachment involving the macula, left eye |
| Diabetes mellitus | ICD-10-CM Diagnosis | E08.3523 | Diabetes (type unknown) | Diabetes mellitus due to underlying condition with proliferative diabetic retinopathy with traction retinal detachment involving the macula, bilateral |
| Diabetes mellitus | ICD-10-CM Diagnosis | E08.3529 | Diabetes (type unknown) | Diabetes mellitus due to underlying condition with proliferative diabetic retinopathy with traction retinal detachment involving the macula, unspecified eye |
| Diabetes mellitus | ICD-10-CM Diagnosis | E08.353 | Diabetes (type unknown) | Diabetes mellitus due to underlying condition with proliferative diabetic retinopathy with traction retinal detachment not involving the macula |
| Diabetes mellitus | ICD-10-CM Diagnosis | E08.3531 | Diabetes (type unknown) | Diabetes mellitus due to underlying condition with proliferative diabetic retinopathy with traction retinal detachment not involving the macula, right eye |
| Diabetes mellitus | ICD-10-CM Diagnosis | E08.3532 | Diabetes (type unknown) | Diabetes mellitus due to underlying condition with proliferative diabetic retinopathy with traction retinal detachment not involving the macula, left eye |
| Diabetes mellitus | ICD-10-CM Diagnosis | E08.3533 | Diabetes (type unknown) | Diabetes mellitus due to underlying condition with proliferative diabetic retinopathy with traction retinal detachment not involving the macula, bilateral |
| Diabetes mellitus | ICD-10-CM Diagnosis | E08.3539 | Diabetes (type unknown) | Diabetes mellitus due to underlying condition with proliferative diabetic retinopathy with traction retinal detachment not involving the macula, unspecified eye |
| Diabetes mellitus | ICD-10-CM Diagnosis | E08.354 | Diabetes (type unknown) | Diabetes mellitus due to underlying condition with proliferative diabetic retinopathy with combined traction retinal detachment and rhegmatogenous retinal detachment |
| Diabetes mellitus | ICD-10-CM Diagnosis | E08.3541 | Diabetes (type unknown) | Diabetes mellitus due to underlying condition with proliferative diabetic retinopathy with combined traction retinal detachment and rhegmatogenous retinal detachment, right eye |
| Diabetes mellitus | ICD-10-CM Diagnosis | E08.3542 | Diabetes (type unknown) | Diabetes mellitus due to underlying condition with proliferative diabetic retinopathy with combined traction retinal detachment and rhegmatogenous retinal detachment, left eye |
| Diabetes mellitus | ICD-10-CM Diagnosis | E08.3543 | Diabetes (type unknown) | Diabetes mellitus due to underlying condition with proliferative diabetic retinopathy with combined traction retinal detachment and rhegmatogenous retinal detachment, bilateral |
| Diabetes mellitus | ICD-10-CM Diagnosis | E08.3549 | Diabetes (type unknown) | Diabetes mellitus due to underlying condition with proliferative diabetic retinopathy with combined traction retinal detachment and rhegmatogenous retinal detachment, unspecified eye |
| Diabetes mellitus | ICD-10-CM Diagnosis | E08.355 | Diabetes (type unknown) | Diabetes mellitus due to underlying condition with stable proliferative diabetic retinopathy |
| Diabetes mellitus | ICD-10-CM Diagnosis | E08.3551 | Diabetes (type unknown) | Diabetes mellitus due to underlying condition with stable proliferative diabetic retinopathy, right eye |
| Diabetes mellitus | ICD-10-CM Diagnosis | E08.3552 | Diabetes (type unknown) | Diabetes mellitus due to underlying condition with stable proliferative diabetic retinopathy, left eye |
| Diabetes mellitus | ICD-10-CM Diagnosis | E08.3553 | Diabetes (type unknown) | Diabetes mellitus due to underlying condition with stable proliferative diabetic retinopathy, bilateral |
| Diabetes mellitus | ICD-10-CM Diagnosis | E08.3559 | Diabetes (type unknown) | Diabetes mellitus due to underlying condition with stable proliferative diabetic retinopathy, unspecified eye |
| Diabetes mellitus | ICD-10-CM Diagnosis | E08.359 | Diabetes (type unknown) | Diabetes mellitus due to underlying condition with proliferative diabetic retinopathy without macular edema |
| Diabetes mellitus | ICD-10-CM Diagnosis | E08.3591 | Diabetes (type unknown) | Diabetes mellitus due to underlying condition with proliferative diabetic retinopathy without macular edema, right eye |
| Diabetes mellitus | ICD-10-CM Diagnosis | E08.3592 | Diabetes (type unknown) | Diabetes mellitus due to underlying condition with proliferative diabetic retinopathy without macular edema, left eye |
| Diabetes mellitus | ICD-10-CM Diagnosis | E08.3593 | Diabetes (type unknown) | Diabetes mellitus due to underlying condition with proliferative diabetic retinopathy without macular edema, bilateral |
| Diabetes mellitus | ICD-10-CM Diagnosis | E08.3599 | Diabetes (type unknown) | Diabetes mellitus due to underlying condition with proliferative diabetic retinopathy without macular edema, unspecified eye |
| Diabetes mellitus | ICD-10-CM Diagnosis | E08.36 | Diabetes (type unknown) | Diabetes mellitus due to underlying condition with diabetic cataract |
| Diabetes mellitus | ICD-10-CM Diagnosis | E08.37 | Diabetes (type unknown) | Diabetes mellitus due to underlying condition with diabetic macular edema, resolved following treatment |
| Diabetes mellitus | ICD-10-CM Diagnosis | E08.37X1 | Diabetes (type unknown) | Diabetes mellitus due to underlying condition with diabetic macular edema, resolved following treatment, right eye |
| Diabetes mellitus | ICD-10-CM Diagnosis | E08.37X2 | Diabetes (type unknown) | Diabetes mellitus due to underlying condition with diabetic macular edema, resolved following treatment, left eye |
| Diabetes mellitus | ICD-10-CM Diagnosis | E08.37X3 | Diabetes (type unknown) | Diabetes mellitus due to underlying condition with diabetic macular edema, resolved following treatment, bilateral |
| Diabetes mellitus | ICD-10-CM Diagnosis | E08.37X9 | Diabetes (type unknown) | Diabetes mellitus due to underlying condition with diabetic macular edema, resolved following treatment, unspecified eye |
| Diabetes mellitus | ICD-10-CM Diagnosis | E08.39 | Diabetes (type unknown) | Diabetes mellitus due to underlying condition with other diabetic ophthalmic complication |
| Diabetes mellitus | ICD-10-CM Diagnosis | E08.4 | Diabetes (type unknown) | Diabetes mellitus due to underlying condition with neurological complications |
| Diabetes mellitus | ICD-10-CM Diagnosis | E08.40 | Diabetes (type unknown) | Diabetes mellitus due to underlying condition with diabetic neuropathy, unspecified |
| Diabetes mellitus | ICD-10-CM Diagnosis | E08.41 | Diabetes (type unknown) | Diabetes mellitus due to underlying condition with diabetic mononeuropathy |
| Diabetes mellitus | ICD-10-CM Diagnosis | E08.42 | Diabetes (type unknown) | Diabetes mellitus due to underlying condition with diabetic polyneuropathy |
| Diabetes mellitus | ICD-10-CM Diagnosis | E08.43 | Diabetes (type unknown) | Diabetes mellitus due to underlying condition with diabetic autonomic (poly)neuropathy |
| Diabetes mellitus | ICD-10-CM Diagnosis | E08.44 | Diabetes (type unknown) | Diabetes mellitus due to underlying condition with diabetic amyotrophy |
| Diabetes mellitus | ICD-10-CM Diagnosis | E08.49 | Diabetes (type unknown) | Diabetes mellitus due to underlying condition with other diabetic neurological complication |
| Diabetes mellitus | ICD-10-CM Diagnosis | E08.5 | Diabetes (type unknown) | Diabetes mellitus due to underlying condition with circulatory complications |
| Diabetes mellitus | ICD-10-CM Diagnosis | E08.51 | Diabetes (type unknown) | Diabetes mellitus due to underlying condition with diabetic peripheral angiopathy without gangrene |
| Diabetes mellitus | ICD-10-CM Diagnosis | E08.52 | Diabetes (type unknown) | Diabetes mellitus due to underlying condition with diabetic peripheral angiopathy with gangrene |
| Diabetes mellitus | ICD-10-CM Diagnosis | E08.59 | Diabetes (type unknown) | Diabetes mellitus due to underlying condition with other circulatory complications |
| Diabetes mellitus | ICD-10-CM Diagnosis | E08.6 | Diabetes (type unknown) | Diabetes mellitus due to underlying condition with other specified complications |
| Diabetes mellitus | ICD-10-CM Diagnosis | E08.61 | Diabetes (type unknown) | Diabetes mellitus due to underlying condition with diabetic arthropathy |
| Diabetes mellitus | ICD-10-CM Diagnosis | E08.610 | Diabetes (type unknown) | Diabetes mellitus due to underlying condition with diabetic neuropathic arthropathy |
| Diabetes mellitus | ICD-10-CM Diagnosis | E08.618 | Diabetes (type unknown) | Diabetes mellitus due to underlying condition with other diabetic arthropathy |
| Diabetes mellitus | ICD-10-CM Diagnosis | E08.62 | Diabetes (type unknown) | Diabetes mellitus due to underlying condition with skin complications |
| Diabetes mellitus | ICD-10-CM Diagnosis | E08.620 | Diabetes (type unknown) | Diabetes mellitus due to underlying condition with diabetic dermatitis |
| Diabetes mellitus | ICD-10-CM Diagnosis | E08.621 | Diabetes (type unknown) | Diabetes mellitus due to underlying condition with foot ulcer |
| Diabetes mellitus | ICD-10-CM Diagnosis | E08.622 | Diabetes (type unknown) | Diabetes mellitus due to underlying condition with other skin ulcer |
| Diabetes mellitus | ICD-10-CM Diagnosis | E08.628 | Diabetes (type unknown) | Diabetes mellitus due to underlying condition with other skin complications |
| Diabetes mellitus | ICD-10-CM Diagnosis | E08.63 | Diabetes (type unknown) | Diabetes mellitus due to underlying condition with oral complications |
| Diabetes mellitus | ICD-10-CM Diagnosis | E08.630 | Diabetes (type unknown) | Diabetes mellitus due to underlying condition with periodontal disease |
| Diabetes mellitus | ICD-10-CM Diagnosis | E08.638 | Diabetes (type unknown) | Diabetes mellitus due to underlying condition with other oral complications |
| Diabetes mellitus | ICD-10-CM Diagnosis | E08.64 | Diabetes (type unknown) | Diabetes mellitus due to underlying condition with hypoglycemia |
| Diabetes mellitus | ICD-10-CM Diagnosis | E08.641 | Diabetes (type unknown) | Diabetes mellitus due to underlying condition with hypoglycemia with coma |
| Diabetes mellitus | ICD-10-CM Diagnosis | E08.649 | Diabetes (type unknown) | Diabetes mellitus due to underlying condition with hypoglycemia without coma |
| Diabetes mellitus | ICD-10-CM Diagnosis | E08.65 | Diabetes (type unknown) | Diabetes mellitus due to underlying condition with hyperglycemia |
| Diabetes mellitus | ICD-10-CM Diagnosis | E08.69 | Diabetes (type unknown) | Diabetes mellitus due to underlying condition with other specified complication |
| Diabetes mellitus | ICD-10-CM Diagnosis | E08.8 | Diabetes (type unknown) | Diabetes mellitus due to underlying condition with unspecified complications |
| Diabetes mellitus | ICD-10-CM Diagnosis | E08.9 | Diabetes (type unknown) | Diabetes mellitus due to underlying condition without complications |
| Diabetes mellitus | ICD-10-CM Diagnosis | E09 | Diabetes (type unknown) | Drug or chemical induced diabetes mellitus |
| Diabetes mellitus | ICD-10-CM Diagnosis | E09.0 | Diabetes (type unknown) | Drug or chemical induced diabetes mellitus with hyperosmolarity |
| Diabetes mellitus | ICD-10-CM Diagnosis | E09.00 | Diabetes (type unknown) | Drug or chemical induced diabetes mellitus with hyperosmolarity without nonketotic hyperglycemic-hyperosmolar coma (NKHHC) |
| Diabetes mellitus | ICD-10-CM Diagnosis | E09.01 | Diabetes (type unknown) | Drug or chemical induced diabetes mellitus with hyperosmolarity with coma |
| Diabetes mellitus | ICD-10-CM Diagnosis | E09.2 | Diabetes (type unknown) | Drug or chemical induced diabetes mellitus with kidney complications |
| Diabetes mellitus | ICD-10-CM Diagnosis | E09.21 | Diabetes (type unknown) | Drug or chemical induced diabetes mellitus with diabetic nephropathy |
| Diabetes mellitus | ICD-10-CM Diagnosis | E09.22 | Diabetes (type unknown) | Drug or chemical induced diabetes mellitus with diabetic chronic kidney disease |
| Diabetes mellitus | ICD-10-CM Diagnosis | E09.29 | Diabetes (type unknown) | Drug or chemical induced diabetes mellitus with other diabetic kidney complication |
| Diabetes mellitus | ICD-10-CM Diagnosis | E09.3 | Diabetes (type unknown) | Drug or chemical induced diabetes mellitus with ophthalmic complications |
| Diabetes mellitus | ICD-10-CM Diagnosis | E09.31 | Diabetes (type unknown) | Drug or chemical induced diabetes mellitus with unspecified diabetic retinopathy |
| Diabetes mellitus | ICD-10-CM Diagnosis | E09.311 | Diabetes (type unknown) | Drug or chemical induced diabetes mellitus with unspecified diabetic retinopathy with macular edema |
| Diabetes mellitus | ICD-10-CM Diagnosis | E09.319 | Diabetes (type unknown) | Drug or chemical induced diabetes mellitus with unspecified diabetic retinopathy without macular edema |
| Diabetes mellitus | ICD-10-CM Diagnosis | E09.32 | Diabetes (type unknown) | Drug or chemical induced diabetes mellitus with mild nonproliferative diabetic retinopathy |
| Diabetes mellitus | ICD-10-CM Diagnosis | E09.321 | Diabetes (type unknown) | Drug or chemical induced diabetes mellitus with mild nonproliferative diabetic retinopathy with macular edema |
| Diabetes mellitus | ICD-10-CM Diagnosis | E09.3211 | Diabetes (type unknown) | Drug or chemical induced diabetes mellitus with mild nonproliferative diabetic retinopathy with macular edema, right eye |
| Diabetes mellitus | ICD-10-CM Diagnosis | E09.3212 | Diabetes (type unknown) | Drug or chemical induced diabetes mellitus with mild nonproliferative diabetic retinopathy with macular edema, left eye |
| Diabetes mellitus | ICD-10-CM Diagnosis | E09.3213 | Diabetes (type unknown) | Drug or chemical induced diabetes mellitus with mild nonproliferative diabetic retinopathy with macular edema, bilateral |
| Diabetes mellitus | ICD-10-CM Diagnosis | E09.3219 | Diabetes (type unknown) | Drug or chemical induced diabetes mellitus with mild nonproliferative diabetic retinopathy with macular edema, unspecified eye |
| Diabetes mellitus | ICD-10-CM Diagnosis | E09.329 | Diabetes (type unknown) | Drug or chemical induced diabetes mellitus with mild nonproliferative diabetic retinopathy without macular edema |
| Diabetes mellitus | ICD-10-CM Diagnosis | E09.3292 | Diabetes (type unknown) | Drug or chemical induced diabetes mellitus with mild nonproliferative diabetic retinopathy without macular edema, left eye |
| Diabetes mellitus | ICD-10-CM Diagnosis | E09.3293 | Diabetes (type unknown) | Drug or chemical induced diabetes mellitus with mild nonproliferative diabetic retinopathy without macular edema, bilateral |
| Diabetes mellitus | ICD-10-CM Diagnosis | E09.3299 | Diabetes (type unknown) | Drug or chemical induced diabetes mellitus with mild nonproliferative diabetic retinopathy without macular edema, unspecified eye |
| Diabetes mellitus | ICD-10-CM Diagnosis | E09.33 | Diabetes (type unknown) | Drug or chemical induced diabetes mellitus with moderate nonproliferative diabetic retinopathy |
| Diabetes mellitus | ICD-10-CM Diagnosis | E09.331 | Diabetes (type unknown) | Drug or chemical induced diabetes mellitus with moderate nonproliferative diabetic retinopathy with macular edema |
| Diabetes mellitus | ICD-10-CM Diagnosis | E09.3311 | Diabetes (type unknown) | Drug or chemical induced diabetes mellitus with moderate nonproliferative diabetic retinopathy with macular edema, right eye |
| Diabetes mellitus | ICD-10-CM Diagnosis | E09.3312 | Diabetes (type unknown) | Drug or chemical induced diabetes mellitus with moderate nonproliferative diabetic retinopathy with macular edema, left eye |
| Diabetes mellitus | ICD-10-CM Diagnosis | E09.3313 | Diabetes (type unknown) | Drug or chemical induced diabetes mellitus with moderate nonproliferative diabetic retinopathy with macular edema, bilateral |
| Diabetes mellitus | ICD-10-CM Diagnosis | E09.3319 | Diabetes (type unknown) | Drug or chemical induced diabetes mellitus with moderate nonproliferative diabetic retinopathy with macular edema, unspecified eye |
| Diabetes mellitus | ICD-10-CM Diagnosis | E09.339 | Diabetes (type unknown) | Drug or chemical induced diabetes mellitus with moderate nonproliferative diabetic retinopathy without macular edema |
| Diabetes mellitus | ICD-10-CM Diagnosis | E09.3391 | Diabetes (type unknown) | Drug or chemical induced diabetes mellitus with moderate nonproliferative diabetic retinopathy without macular edema, right eye |
| Diabetes mellitus | ICD-10-CM Diagnosis | E09.3392 | Diabetes (type unknown) | Drug or chemical induced diabetes mellitus with moderate nonproliferative diabetic retinopathy without macular edema, left eye |
| Diabetes mellitus | ICD-10-CM Diagnosis | E09.3393 | Diabetes (type unknown) | Drug or chemical induced diabetes mellitus with moderate nonproliferative diabetic retinopathy without macular edema, bilateral |
| Diabetes mellitus | ICD-10-CM Diagnosis | E09.3399 | Diabetes (type unknown) | Drug or chemical induced diabetes mellitus with moderate nonproliferative diabetic retinopathy without macular edema, unspecified eye |
| Diabetes mellitus | ICD-10-CM Diagnosis | E09.34 | Diabetes (type unknown) | Drug or chemical induced diabetes mellitus with severe nonproliferative diabetic retinopathy |
| Diabetes mellitus | ICD-10-CM Diagnosis | E09.341 | Diabetes (type unknown) | Drug or chemical induced diabetes mellitus with severe nonproliferative diabetic retinopathy with macular edema |
| Diabetes mellitus | ICD-10-CM Diagnosis | E09.3411 | Diabetes (type unknown) | Drug or chemical induced diabetes mellitus with severe nonproliferative diabetic retinopathy with macular edema, right eye |
| Diabetes mellitus | ICD-10-CM Diagnosis | E09.3412 | Diabetes (type unknown) | Drug or chemical induced diabetes mellitus with severe nonproliferative diabetic retinopathy with macular edema, left eye |
| Diabetes mellitus | ICD-10-CM Diagnosis | E09.3413 | Diabetes (type unknown) | Drug or chemical induced diabetes mellitus with severe nonproliferative diabetic retinopathy with macular edema, bilateral |
| Diabetes mellitus | ICD-10-CM Diagnosis | E09.3419 | Diabetes (type unknown) | Drug or chemical induced diabetes mellitus with severe nonproliferative diabetic retinopathy with macular edema, unspecified eye |
| Diabetes mellitus | ICD-10-CM Diagnosis | E09.349 | Diabetes (type unknown) | Drug or chemical induced diabetes mellitus with severe nonproliferative diabetic retinopathy without macular edema |
| Diabetes mellitus | ICD-10-CM Diagnosis | E09.3491 | Diabetes (type unknown) | Drug or chemical induced diabetes mellitus with severe nonproliferative diabetic retinopathy without macular edema, right eye |
| Diabetes mellitus | ICD-10-CM Diagnosis | E09.3492 | Diabetes (type unknown) | Drug or chemical induced diabetes mellitus with severe nonproliferative diabetic retinopathy without macular edema, left eye |
| Diabetes mellitus | ICD-10-CM Diagnosis | E09.3493 | Diabetes (type unknown) | Drug or chemical induced diabetes mellitus with severe nonproliferative diabetic retinopathy without macular edema, bilateral |
| Diabetes mellitus | ICD-10-CM Diagnosis | E09.3499 | Diabetes (type unknown) | Drug or chemical induced diabetes mellitus with severe nonproliferative diabetic retinopathy without macular edema, unspecified eye |
| Diabetes mellitus | ICD-10-CM Diagnosis | E09.35 | Diabetes (type unknown) | Drug or chemical induced diabetes mellitus with proliferative diabetic retinopathy |
| Diabetes mellitus | ICD-10-CM Diagnosis | E09.351 | Diabetes (type unknown) | Drug or chemical induced diabetes mellitus with proliferative diabetic retinopathy with macular edema |
| Diabetes mellitus | ICD-10-CM Diagnosis | E09.3511 | Diabetes (type unknown) | Drug or chemical induced diabetes mellitus with proliferative diabetic retinopathy with macular edema, right eye |
| Diabetes mellitus | ICD-10-CM Diagnosis | E09.3512 | Diabetes (type unknown) | Drug or chemical induced diabetes mellitus with proliferative diabetic retinopathy with macular edema, left eye |
| Diabetes mellitus | ICD-10-CM Diagnosis | E09.3513 | Diabetes (type unknown) | Drug or chemical induced diabetes mellitus with proliferative diabetic retinopathy with macular edema, bilateral |
| Diabetes mellitus | ICD-10-CM Diagnosis | E09.3519 | Diabetes (type unknown) | Drug or chemical induced diabetes mellitus with proliferative diabetic retinopathy with macular edema, unspecified eye |
| Diabetes mellitus | ICD-10-CM Diagnosis | E09.352 | Diabetes (type unknown) | Drug or chemical induced diabetes mellitus with proliferative diabetic retinopathy with traction retinal detachment involving the macula |
| Diabetes mellitus | ICD-10-CM Diagnosis | E09.3521 | Diabetes (type unknown) | Drug or chemical induced diabetes mellitus with proliferative diabetic retinopathy with traction retinal detachment involving the macula, right eye |
| Diabetes mellitus | ICD-10-CM Diagnosis | E09.3522 | Diabetes (type unknown) | Drug or chemical induced diabetes mellitus with proliferative diabetic retinopathy with traction retinal detachment involving the macula, left eye |
| Diabetes mellitus | ICD-10-CM Diagnosis | E09.3523 | Diabetes (type unknown) | Drug or chemical induced diabetes mellitus with proliferative diabetic retinopathy with traction retinal detachment involving the macula, bilateral |
| Diabetes mellitus | ICD-10-CM Diagnosis | E09.3529 | Diabetes (type unknown) | Drug or chemical induced diabetes mellitus with proliferative diabetic retinopathy with traction retinal detachment involving the macula, unspecified eye |
| Diabetes mellitus | ICD-10-CM Diagnosis | E09.353 | Diabetes (type unknown) | Drug or chemical induced diabetes mellitus with proliferative diabetic retinopathy with traction retinal detachment not involving the macula |
| Diabetes mellitus | ICD-10-CM Diagnosis | E09.3531 | Diabetes (type unknown) | Drug or chemical induced diabetes mellitus with proliferative diabetic retinopathy with traction retinal detachment not involving the macula, right eye |
| Diabetes mellitus | ICD-10-CM Diagnosis | E09.3532 | Diabetes (type unknown) | Drug or chemical induced diabetes mellitus with proliferative diabetic retinopathy with traction retinal detachment not involving the macula, left eye |
| Diabetes mellitus | ICD-10-CM Diagnosis | E09.3533 | Diabetes (type unknown) | Drug or chemical induced diabetes mellitus with proliferative diabetic retinopathy with traction retinal detachment not involving the macula, bilateral |
| Diabetes mellitus | ICD-10-CM Diagnosis | E09.3539 | Diabetes (type unknown) | Drug or chemical induced diabetes mellitus with proliferative diabetic retinopathy with traction retinal detachment not involving the macula, unspecified eye |
| Diabetes mellitus | ICD-10-CM Diagnosis | E09.354 | Diabetes (type unknown) | Drug or chemical induced diabetes mellitus with proliferative diabetic retinopathy with combined traction retinal detachment and rhegmatogenous retinal detachment |
| Diabetes mellitus | ICD-10-CM Diagnosis | E09.3541 | Diabetes (type unknown) | Drug or chemical induced diabetes mellitus with proliferative diabetic retinopathy with combined traction retinal detachment and rhegmatogenous retinal detachment, right eye |
| Diabetes mellitus | ICD-10-CM Diagnosis | E09.3542 | Diabetes (type unknown) | Drug or chemical induced diabetes mellitus with proliferative diabetic retinopathy with combined traction retinal detachment and rhegmatogenous retinal detachment, left eye |
| Diabetes mellitus | ICD-10-CM Diagnosis | E09.3543 | Diabetes (type unknown) | Drug or chemical induced diabetes mellitus with proliferative diabetic retinopathy with combined traction retinal detachment and rhegmatogenous retinal detachment, bilateral |
| Diabetes mellitus | ICD-10-CM Diagnosis | E09.3549 | Diabetes (type unknown) | Drug or chemical induced diabetes mellitus with proliferative diabetic retinopathy with combined traction retinal detachment and rhegmatogenous retinal detachment, unspecified eye |
| Diabetes mellitus | ICD-10-CM Diagnosis | E09.355 | Diabetes (type unknown) | Drug or chemical induced diabetes mellitus with stable proliferative diabetic retinopathy |
| Diabetes mellitus | ICD-10-CM Diagnosis | E09.3551 | Diabetes (type unknown) | Drug or chemical induced diabetes mellitus with stable proliferative diabetic retinopathy, right eye |
| Diabetes mellitus | ICD-10-CM Diagnosis | E09.3552 | Diabetes (type unknown) | Drug or chemical induced diabetes mellitus with stable proliferative diabetic retinopathy, left eye |
| Diabetes mellitus | ICD-10-CM Diagnosis | E09.3553 | Diabetes (type unknown) | Drug or chemical induced diabetes mellitus with stable proliferative diabetic retinopathy, bilateral |
| Diabetes mellitus | ICD-10-CM Diagnosis | E09.3559 | Diabetes (type unknown) | Drug or chemical induced diabetes mellitus with stable proliferative diabetic retinopathy, unspecified eye |
| Diabetes mellitus | ICD-10-CM Diagnosis | E09.359 | Diabetes (type unknown) | Drug or chemical induced diabetes mellitus with proliferative diabetic retinopathy without macular edema |
| Diabetes mellitus | ICD-10-CM Diagnosis | E09.3591 | Diabetes (type unknown) | Drug or chemical induced diabetes mellitus with proliferative diabetic retinopathy without macular edema, right eye |
| Diabetes mellitus | ICD-10-CM Diagnosis | E09.3592 | Diabetes (type unknown) | Drug or chemical induced diabetes mellitus with proliferative diabetic retinopathy without macular edema, left eye |
| Diabetes mellitus | ICD-10-CM Diagnosis | E09.3593 | Diabetes (type unknown) | Drug or chemical induced diabetes mellitus with proliferative diabetic retinopathy without macular edema, bilateral |
| Diabetes mellitus | ICD-10-CM Diagnosis | E09.3599 | Diabetes (type unknown) | Drug or chemical induced diabetes mellitus with proliferative diabetic retinopathy without macular edema, unspecified eye |
| Diabetes mellitus | ICD-10-CM Diagnosis | E09.36 | Diabetes (type unknown) | Drug or chemical induced diabetes mellitus with diabetic cataract |
| Diabetes mellitus | ICD-10-CM Diagnosis | E09.37 | Diabetes (type unknown) | Drug or chemical induced diabetes mellitus with diabetic macular edema, resolved following treatment |
| Diabetes mellitus | ICD-10-CM Diagnosis | E09.37X1 | Diabetes (type unknown) | Drug or chemical induced diabetes mellitus with diabetic macular edema, resolved following treatment, right eye |
| Diabetes mellitus | ICD-10-CM Diagnosis | E09.37X2 | Diabetes (type unknown) | Drug or chemical induced diabetes mellitus with diabetic macular edema, resolved following treatment, left eye |
| Diabetes mellitus | ICD-10-CM Diagnosis | E09.37X3 | Diabetes (type unknown) | Drug or chemical induced diabetes mellitus with diabetic macular edema, resolved following treatment, bilateral |
| Diabetes mellitus | ICD-10-CM Diagnosis | E09.37X9 | Diabetes (type unknown) | Drug or chemical induced diabetes mellitus with diabetic macular edema, resolved following treatment, unspecified eye |
| Diabetes mellitus | ICD-10-CM Diagnosis | E09.39 | Diabetes (type unknown) | Drug or chemical induced diabetes mellitus with other diabetic ophthalmic complication |
| Diabetes mellitus | ICD-10-CM Diagnosis | E09.4 | Diabetes (type unknown) | Drug or chemical induced diabetes mellitus with neurological complications |
| Diabetes mellitus | ICD-10-CM Diagnosis | E09.40 | Diabetes (type unknown) | Drug or chemical induced diabetes mellitus with neurological complications with diabetic neuropathy, unspecified |
| Diabetes mellitus | ICD-10-CM Diagnosis | E09.41 | Diabetes (type unknown) | Drug or chemical induced diabetes mellitus with neurological complications with diabetic mononeuropathy |
| Diabetes mellitus | ICD-10-CM Diagnosis | E09.42 | Diabetes (type unknown) | Drug or chemical induced diabetes mellitus with neurological complications with diabetic polyneuropathy |
| Diabetes mellitus | ICD-10-CM Diagnosis | E09.43 | Diabetes (type unknown) | Drug or chemical induced diabetes mellitus with neurological complications with diabetic autonomic (poly)neuropathy |
| Diabetes mellitus | ICD-10-CM Diagnosis | E09.44 | Diabetes (type unknown) | Drug or chemical induced diabetes mellitus with neurological complications with diabetic amyotrophy |
| Diabetes mellitus | ICD-10-CM Diagnosis | E09.49 | Diabetes (type unknown) | Drug or chemical induced diabetes mellitus with neurological complications with other diabetic neurological complication |
| Diabetes mellitus | ICD-10-CM Diagnosis | E09.5 | Diabetes (type unknown) | Drug or chemical induced diabetes mellitus with circulatory complications |
| Diabetes mellitus | ICD-10-CM Diagnosis | E09.51 | Diabetes (type unknown) | Drug or chemical induced diabetes mellitus with diabetic peripheral angiopathy without gangrene |
| Diabetes mellitus | ICD-10-CM Diagnosis | E09.52 | Diabetes (type unknown) | Drug or chemical induced diabetes mellitus with diabetic peripheral angiopathy with gangrene |
| Diabetes mellitus | ICD-10-CM Diagnosis | E09.59 | Diabetes (type unknown) | Drug or chemical induced diabetes mellitus with other circulatory complications |
| Diabetes mellitus | ICD-10-CM Diagnosis | E09.6 | Diabetes (type unknown) | Drug or chemical induced diabetes mellitus with other specified complications |
| Diabetes mellitus | ICD-10-CM Diagnosis | E09.61 | Diabetes (type unknown) | Drug or chemical induced diabetes mellitus with diabetic arthropathy |
| Diabetes mellitus | ICD-10-CM Diagnosis | E09.610 | Diabetes (type unknown) | Drug or chemical induced diabetes mellitus with diabetic neuropathic arthropathy |
| Diabetes mellitus | ICD-10-CM Diagnosis | E09.618 | Diabetes (type unknown) | Drug or chemical induced diabetes mellitus with other diabetic arthropathy |
| Diabetes mellitus | ICD-10-CM Diagnosis | E09.62 | Diabetes (type unknown) | Drug or chemical induced diabetes mellitus with skin complications |
| Diabetes mellitus | ICD-10-CM Diagnosis | E09.620 | Diabetes (type unknown) | Drug or chemical induced diabetes mellitus with diabetic dermatitis |
| Diabetes mellitus | ICD-10-CM Diagnosis | E09.621 | Diabetes (type unknown) | Drug or chemical induced diabetes mellitus with foot ulcer |
| Diabetes mellitus | ICD-10-CM Diagnosis | E09.622 | Diabetes (type unknown) | Drug or chemical induced diabetes mellitus with other skin ulcer |
| Diabetes mellitus | ICD-10-CM Diagnosis | E09.628 | Diabetes (type unknown) | Drug or chemical induced diabetes mellitus with other skin complications |
| Diabetes mellitus | ICD-10-CM Diagnosis | E09.63 | Diabetes (type unknown) | Drug or chemical induced diabetes mellitus with oral complications |
| Diabetes mellitus | ICD-10-CM Diagnosis | E09.630 | Diabetes (type unknown) | Drug or chemical induced diabetes mellitus with periodontal disease |
| Diabetes mellitus | ICD-10-CM Diagnosis | E09.638 | Diabetes (type unknown) | Drug or chemical induced diabetes mellitus with other oral complications |
| Diabetes mellitus | ICD-10-CM Diagnosis | E09.64 | Diabetes (type unknown) | Drug or chemical induced diabetes mellitus with hypoglycemia |
| Diabetes mellitus | ICD-10-CM Diagnosis | E09.641 | Diabetes (type unknown) | Drug or chemical induced diabetes mellitus with hypoglycemia with coma |
| Diabetes mellitus | ICD-10-CM Diagnosis | E09.649 | Diabetes (type unknown) | Drug or chemical induced diabetes mellitus with hypoglycemia without coma |
| Diabetes mellitus | ICD-10-CM Diagnosis | E09.65 | Diabetes (type unknown) | Drug or chemical induced diabetes mellitus with hyperglycemia |
| Diabetes mellitus | ICD-10-CM Diagnosis | E09.69 | Diabetes (type unknown) | Drug or chemical induced diabetes mellitus with other specified complication |
| Diabetes mellitus | ICD-10-CM Diagnosis | E09.8 | Diabetes (type unknown) | Drug or chemical induced diabetes mellitus with unspecified complications |
| Diabetes mellitus | ICD-10-CM Diagnosis | E09.9 | Diabetes (type unknown) | Drug or chemical induced diabetes mellitus without complications |
| Diabetes mellitus | ICD-10-CM Diagnosis | E13 | Diabetes (type unknown) | Other specified diabetes mellitus |
| Diabetes mellitus | ICD-10-CM Diagnosis | E13.0 | Diabetes (type unknown) | Other specified diabetes mellitus with hyperosmolarity |
| Diabetes mellitus | ICD-10-CM Diagnosis | E13.00 | Diabetes (type unknown) | Other specified diabetes mellitus with hyperosmolarity without nonketotic hyperglycemic-hyperosmolar coma (NKHHC) |
| Diabetes mellitus | ICD-10-CM Diagnosis | E13.6 | Diabetes (type unknown) | Other specified diabetes mellitus with other specified complications |
| Diabetes mellitus | ICD-10-CM Diagnosis | E13.61 | Diabetes (type unknown) | Other specified diabetes mellitus with diabetic arthropathy |
| Diabetes mellitus | ICD-10-CM Diagnosis | E13.610 | Diabetes (type unknown) | Other specified diabetes mellitus with diabetic neuropathic arthropathy |
| Diabetes mellitus | ICD-10-CM Diagnosis | E13.618 | Diabetes (type unknown) | Other specified diabetes mellitus with other diabetic arthropathy |
| Diabetes mellitus | ICD-10-CM Diagnosis | E13.621 | Diabetes (type unknown) | Other specified diabetes mellitus with foot ulcer |
| Diabetes mellitus | ICD-10-CM Diagnosis | E13.622 | Diabetes (type unknown) | Other specified diabetes mellitus with other skin ulcer |
| Diabetes mellitus | ICD-10-CM Diagnosis | E13.63 | Diabetes (type unknown) | Other specified diabetes mellitus with oral complications |
| Diabetes mellitus | ICD-10-CM Diagnosis | E13.630 | Diabetes (type unknown) | Other specified diabetes mellitus with periodontal disease |
| Diabetes mellitus | ICD-10-CM Diagnosis | E13.638 | Diabetes (type unknown) | Other specified diabetes mellitus with other oral complications |
| Diabetes mellitus | ICD-10-CM Diagnosis | E13.64 | Diabetes (type unknown) | Other specified diabetes mellitus with hypoglycemia |
| Diabetes mellitus | ICD-10-CM Diagnosis | E13.649 | Diabetes (type unknown) | Other specified diabetes mellitus with hypoglycemia without coma |
| Diabetes mellitus | ICD-10-CM Diagnosis | E13.65 | Diabetes (type unknown) | Other specified diabetes mellitus with hyperglycemia |
| Diabetes mellitus | ICD-10-CM Diagnosis | E13.69 | Diabetes (type unknown) | Other specified diabetes mellitus with other specified complication |
| Diabetes mellitus | ICD-10-CM Diagnosis | E13.8 | Diabetes (type unknown) | Other specified diabetes mellitus with unspecified complications |
| Diabetes mellitus | ICD-10-CM Diagnosis | E13.9 | Diabetes (type unknown) | Other specified diabetes mellitus without complications |
| Diabetes mellitus | ICD-10-CM Diagnosis | E10 | Type I Diabetes | Type 1 diabetes mellitus |
| Diabetes mellitus | ICD-10-CM Diagnosis | E10.1 | Type I Diabetes | Type 1 diabetes mellitus with ketoacidosis |
| Diabetes mellitus | ICD-10-CM Diagnosis | E10.10 | Type I Diabetes | Type 1 diabetes mellitus with ketoacidosis without coma |
| Diabetes mellitus | ICD-10-CM Diagnosis | E10.11 | Type I Diabetes | Type 1 diabetes mellitus with ketoacidosis with coma |
| Diabetes mellitus | ICD-10-CM Diagnosis | E10.2 | Type I Diabetes | Type 1 diabetes mellitus with kidney complications |
| Diabetes mellitus | ICD-10-CM Diagnosis | E10.21 | Type I Diabetes | Type 1 diabetes mellitus with diabetic nephropathy |
| Diabetes mellitus | ICD-10-CM Diagnosis | E10.22 | Type I Diabetes | Type 1 diabetes mellitus with diabetic chronic kidney disease |
| Diabetes mellitus | ICD-10-CM Diagnosis | E10.29 | Type I Diabetes | Type 1 diabetes mellitus with other diabetic kidney complication |
| Diabetes mellitus | ICD-10-CM Diagnosis | E10.3 | Type I Diabetes | Type 1 diabetes mellitus with ophthalmic complications |
| Diabetes mellitus | ICD-10-CM Diagnosis | E10.31 | Type I Diabetes | Type 1 diabetes mellitus with unspecified diabetic retinopathy |
| Diabetes mellitus | ICD-10-CM Diagnosis | E10.311 | Type I Diabetes | Type 1 diabetes mellitus with unspecified diabetic retinopathy with macular edema |
| Diabetes mellitus | ICD-10-CM Diagnosis | E10.319 | Type I Diabetes | Type 1 diabetes mellitus with unspecified diabetic retinopathy without macular edema |
| Diabetes mellitus | ICD-10-CM Diagnosis | E10.32 | Type I Diabetes | Type 1 diabetes mellitus with mild nonproliferative diabetic retinopathy |
| Diabetes mellitus | ICD-10-CM Diagnosis | E10.321 | Type I Diabetes | Type 1 diabetes mellitus with mild nonproliferative diabetic retinopathy with macular edema |
| Diabetes mellitus | ICD-10-CM Diagnosis | E10.3211 | Type I Diabetes | Type 1 diabetes mellitus with mild nonproliferative diabetic retinopathy with macular edema, right eye |
| Diabetes mellitus | ICD-10-CM Diagnosis | E10.3212 | Type I Diabetes | Type 1 diabetes mellitus with mild nonproliferative diabetic retinopathy with macular edema, left eye |
| Diabetes mellitus | ICD-10-CM Diagnosis | E10.3213 | Type I Diabetes | Type 1 diabetes mellitus with mild nonproliferative diabetic retinopathy with macular edema, bilateral |
| Diabetes mellitus | ICD-10-CM Diagnosis | E10.3219 | Type I Diabetes | Type 1 diabetes mellitus with mild nonproliferative diabetic retinopathy with macular edema, unspecified eye |
| Diabetes mellitus | ICD-10-CM Diagnosis | E10.329 | Type I Diabetes | Type 1 diabetes mellitus with mild nonproliferative diabetic retinopathy without macular edema |
| Diabetes mellitus | ICD-10-CM Diagnosis | E10.3291 | Type I Diabetes | Type 1 diabetes mellitus with mild nonproliferative diabetic retinopathy without macular edema, right eye |
| Diabetes mellitus | ICD-10-CM Diagnosis | E10.3292 | Type I Diabetes | Type 1 diabetes mellitus with mild nonproliferative diabetic retinopathy without macular edema, left eye |
| Diabetes mellitus | ICD-10-CM Diagnosis | E10.3293 | Type I Diabetes | Type 1 diabetes mellitus with mild nonproliferative diabetic retinopathy without macular edema, bilateral |
| Diabetes mellitus | ICD-10-CM Diagnosis | E10.3299 | Type I Diabetes | Type 1 diabetes mellitus with mild nonproliferative diabetic retinopathy without macular edema, unspecified eye |
| Diabetes mellitus | ICD-10-CM Diagnosis | E10.33 | Type I Diabetes | Type 1 diabetes mellitus with moderate nonproliferative diabetic retinopathy |
| Diabetes mellitus | ICD-10-CM Diagnosis | E10.331 | Type I Diabetes | Type 1 diabetes mellitus with moderate nonproliferative diabetic retinopathy with macular edema |
| Diabetes mellitus | ICD-10-CM Diagnosis | E10.3311 | Type I Diabetes | Type 1 diabetes mellitus with moderate nonproliferative diabetic retinopathy with macular edema, right eye |
| Diabetes mellitus | ICD-10-CM Diagnosis | E10.3312 | Type I Diabetes | Type 1 diabetes mellitus with moderate nonproliferative diabetic retinopathy with macular edema, left eye |
| Diabetes mellitus | ICD-10-CM Diagnosis | E10.3313 | Type I Diabetes | Type 1 diabetes mellitus with moderate nonproliferative diabetic retinopathy with macular edema, bilateral |
| Diabetes mellitus | ICD-10-CM Diagnosis | E10.3319 | Type I Diabetes | Type 1 diabetes mellitus with moderate nonproliferative diabetic retinopathy with macular edema, unspecified eye |
| Diabetes mellitus | ICD-10-CM Diagnosis | E10.339 | Type I Diabetes | Type 1 diabetes mellitus with moderate nonproliferative diabetic retinopathy without macular edema |
| Diabetes mellitus | ICD-10-CM Diagnosis | E10.3391 | Type I Diabetes | Type 1 diabetes mellitus with moderate nonproliferative diabetic retinopathy without macular edema, right eye |
| Diabetes mellitus | ICD-10-CM Diagnosis | E10.3392 | Type I Diabetes | Type 1 diabetes mellitus with moderate nonproliferative diabetic retinopathy without macular edema, left eye |
| Diabetes mellitus | ICD-10-CM Diagnosis | E10.3393 | Type I Diabetes | Type 1 diabetes mellitus with moderate nonproliferative diabetic retinopathy without macular edema, bilateral |
| Diabetes mellitus | ICD-10-CM Diagnosis | E10.3399 | Type I Diabetes | Type 1 diabetes mellitus with moderate nonproliferative diabetic retinopathy without macular edema, unspecified eye |
| Diabetes mellitus | ICD-10-CM Diagnosis | E10.34 | Type I Diabetes | Type 1 diabetes mellitus with severe nonproliferative diabetic retinopathy |
| Diabetes mellitus | ICD-10-CM Diagnosis | E10.341 | Type I Diabetes | Type 1 diabetes mellitus with severe nonproliferative diabetic retinopathy with macular edema |
| Diabetes mellitus | ICD-10-CM Diagnosis | E10.3411 | Type I Diabetes | Type 1 diabetes mellitus with severe nonproliferative diabetic retinopathy with macular edema, right eye |
| Diabetes mellitus | ICD-10-CM Diagnosis | E10.3412 | Type I Diabetes | Type 1 diabetes mellitus with severe nonproliferative diabetic retinopathy with macular edema, left eye |
| Diabetes mellitus | ICD-10-CM Diagnosis | E10.3413 | Type I Diabetes | Type 1 diabetes mellitus with severe nonproliferative diabetic retinopathy with macular edema, bilateral |
| Diabetes mellitus | ICD-10-CM Diagnosis | E10.3419 | Type I Diabetes | Type 1 diabetes mellitus with severe nonproliferative diabetic retinopathy with macular edema, unspecified eye |
| Diabetes mellitus | ICD-10-CM Diagnosis | E10.349 | Type I Diabetes | Type 1 diabetes mellitus with severe nonproliferative diabetic retinopathy without macular edema |
| Diabetes mellitus | ICD-10-CM Diagnosis | E10.3491 | Type I Diabetes | Type 1 diabetes mellitus with severe nonproliferative diabetic retinopathy without macular edema, right eye |
| Diabetes mellitus | ICD-10-CM Diagnosis | E10.3492 | Type I Diabetes | Type 1 diabetes mellitus with severe nonproliferative diabetic retinopathy without macular edema, left eye |
| Diabetes mellitus | ICD-10-CM Diagnosis | E10.3493 | Type I Diabetes | Type 1 diabetes mellitus with severe nonproliferative diabetic retinopathy without macular edema, bilateral |
| Diabetes mellitus | ICD-10-CM Diagnosis | E10.3499 | Type I Diabetes | Type 1 diabetes mellitus with severe nonproliferative diabetic retinopathy without macular edema, unspecified eye |
| Diabetes mellitus | ICD-10-CM Diagnosis | E10.35 | Type I Diabetes | Type 1 diabetes mellitus with proliferative diabetic retinopathy |
| Diabetes mellitus | ICD-10-CM Diagnosis | E10.351 | Type I Diabetes | Type 1 diabetes mellitus with proliferative diabetic retinopathy with macular edema |
| Diabetes mellitus | ICD-10-CM Diagnosis | E10.3511 | Type I Diabetes | Type 1 diabetes mellitus with proliferative diabetic retinopathy with macular edema, right eye |
| Diabetes mellitus | ICD-10-CM Diagnosis | E10.3512 | Type I Diabetes | Type 1 diabetes mellitus with proliferative diabetic retinopathy with macular edema, left eye |
| Diabetes mellitus | ICD-10-CM Diagnosis | E10.3513 | Type I Diabetes | Type 1 diabetes mellitus with proliferative diabetic retinopathy with macular edema, bilateral |
| Diabetes mellitus | ICD-10-CM Diagnosis | E10.3519 | Type I Diabetes | Type 1 diabetes mellitus with proliferative diabetic retinopathy with macular edema, unspecified eye |
| Diabetes mellitus | ICD-10-CM Diagnosis | E10.352 | Type I Diabetes | Type 1 diabetes mellitus with proliferative diabetic retinopathy with traction retinal detachment involving the macula |
| Diabetes mellitus | ICD-10-CM Diagnosis | E10.3521 | Type I Diabetes | Type 1 diabetes mellitus with proliferative diabetic retinopathy with traction retinal detachment involving the macula, right eye |
| Diabetes mellitus | ICD-10-CM Diagnosis | E10.3522 | Type I Diabetes | Type 1 diabetes mellitus with proliferative diabetic retinopathy with traction retinal detachment involving the macula, left eye |
| Diabetes mellitus | ICD-10-CM Diagnosis | E10.3523 | Type I Diabetes | Type 1 diabetes mellitus with proliferative diabetic retinopathy with traction retinal detachment involving the macula, bilateral |
| Diabetes mellitus | ICD-10-CM Diagnosis | E10.3529 | Type I Diabetes | Type 1 diabetes mellitus with proliferative diabetic retinopathy with traction retinal detachment involving the macula, unspecified eye |
| Diabetes mellitus | ICD-10-CM Diagnosis | E10.353 | Type I Diabetes | Type 1 diabetes mellitus with proliferative diabetic retinopathy with traction retinal detachment not involving the macula |
| Diabetes mellitus | ICD-10-CM Diagnosis | E10.3531 | Type I Diabetes | Type 1 diabetes mellitus with proliferative diabetic retinopathy with traction retinal detachment not involving the macula, right eye |
| Diabetes mellitus | ICD-10-CM Diagnosis | E10.3532 | Type I Diabetes | Type 1 diabetes mellitus with proliferative diabetic retinopathy with traction retinal detachment not involving the macula, left eye |
| Diabetes mellitus | ICD-10-CM Diagnosis | E10.3533 | Type I Diabetes | Type 1 diabetes mellitus with proliferative diabetic retinopathy with traction retinal detachment not involving the macula, bilateral |
| Diabetes mellitus | ICD-10-CM Diagnosis | E10.3539 | Type I Diabetes | Type 1 diabetes mellitus with proliferative diabetic retinopathy with traction retinal detachment not involving the macula, unspecified eye |
| Diabetes mellitus | ICD-10-CM Diagnosis | E10.354 | Type I Diabetes | Type 1 diabetes mellitus with proliferative diabetic retinopathy with combined traction retinal detachment and rhegmatogenous retinal detachment |
| Diabetes mellitus | ICD-10-CM Diagnosis | E10.3541 | Type I Diabetes | Type 1 diabetes mellitus with proliferative diabetic retinopathy with combined traction retinal detachment and rhegmatogenous retinal detachment, right eye |
| Diabetes mellitus | ICD-10-CM Diagnosis | E10.3542 | Type I Diabetes | Type 1 diabetes mellitus with proliferative diabetic retinopathy with combined traction retinal detachment and rhegmatogenous retinal detachment, left eye |
| Diabetes mellitus | ICD-10-CM Diagnosis | E10.3543 | Type I Diabetes | Type 1 diabetes mellitus with proliferative diabetic retinopathy with combined traction retinal detachment and rhegmatogenous retinal detachment, bilateral |
| Diabetes mellitus | ICD-10-CM Diagnosis | E10.3549 | Type I Diabetes | Type 1 diabetes mellitus with proliferative diabetic retinopathy with combined traction retinal detachment and rhegmatogenous retinal detachment, unspecified eye |
| Diabetes mellitus | ICD-10-CM Diagnosis | E10.355 | Type I Diabetes | Type 1 diabetes mellitus with stable proliferative diabetic retinopathy |
| Diabetes mellitus | ICD-10-CM Diagnosis | E10.3551 | Type I Diabetes | Type 1 diabetes mellitus with stable proliferative diabetic retinopathy, right eye |
| Diabetes mellitus | ICD-10-CM Diagnosis | E10.3552 | Type I Diabetes | Type 1 diabetes mellitus with stable proliferative diabetic retinopathy, left eye |
| Diabetes mellitus | ICD-10-CM Diagnosis | E10.3553 | Type I Diabetes | Type 1 diabetes mellitus with stable proliferative diabetic retinopathy, bilateral |
| Diabetes mellitus | ICD-10-CM Diagnosis | E10.3559 | Type I Diabetes | Type 1 diabetes mellitus with stable proliferative diabetic retinopathy, unspecified eye |
| Diabetes mellitus | ICD-10-CM Diagnosis | E10.359 | Type I Diabetes | Type 1 diabetes mellitus with proliferative diabetic retinopathy without macular edema |
| Diabetes mellitus | ICD-10-CM Diagnosis | E10.3591 | Type I Diabetes | Type 1 diabetes mellitus with proliferative diabetic retinopathy without macular edema, right eye |
| Diabetes mellitus | ICD-10-CM Diagnosis | E10.3592 | Type I Diabetes | Type 1 diabetes mellitus with proliferative diabetic retinopathy without macular edema, left eye |
| Diabetes mellitus | ICD-10-CM Diagnosis | E10.3593 | Type I Diabetes | Type 1 diabetes mellitus with proliferative diabetic retinopathy without macular edema, bilateral |
| Diabetes mellitus | ICD-10-CM Diagnosis | E10.3599 | Type I Diabetes | Type 1 diabetes mellitus with proliferative diabetic retinopathy without macular edema, unspecified eye |
| Diabetes mellitus | ICD-10-CM Diagnosis | E10.36 | Type I Diabetes | Type 1 diabetes mellitus with diabetic cataract |
| Diabetes mellitus | ICD-10-CM Diagnosis | E10.37 | Type I Diabetes | Type 1 diabetes mellitus with diabetic macular edema, resolved following treatment |
| Diabetes mellitus | ICD-10-CM Diagnosis | E10.37X1 | Type I Diabetes | Type 1 diabetes mellitus with diabetic macular edema, resolved following treatment, right eye |
| Diabetes mellitus | ICD-10-CM Diagnosis | E10.37X2 | Type I Diabetes | Type 1 diabetes mellitus with diabetic macular edema, resolved following treatment, left eye |
| Diabetes mellitus | ICD-10-CM Diagnosis | E10.37X3 | Type I Diabetes | Type 1 diabetes mellitus with diabetic macular edema, resolved following treatment, bilateral |
| Diabetes mellitus | ICD-10-CM Diagnosis | E10.37X9 | Type I Diabetes | Type 1 diabetes mellitus with diabetic macular edema, resolved following treatment, unspecified eye |
| Diabetes mellitus | ICD-10-CM Diagnosis | E10.39 | Type I Diabetes | Type 1 diabetes mellitus with other diabetic ophthalmic complication |
| Diabetes mellitus | ICD-10-CM Diagnosis | E10.4 | Type I Diabetes | Type 1 diabetes mellitus with neurological complications |
| Diabetes mellitus | ICD-10-CM Diagnosis | E10.40 | Type I Diabetes | Type 1 diabetes mellitus with diabetic neuropathy, unspecified |
| Diabetes mellitus | ICD-10-CM Diagnosis | E10.41 | Type I Diabetes | Type 1 diabetes mellitus with diabetic mononeuropathy |
| Diabetes mellitus | ICD-10-CM Diagnosis | E10.42 | Type I Diabetes | Type 1 diabetes mellitus with diabetic polyneuropathy |
| Diabetes mellitus | ICD-10-CM Diagnosis | E10.43 | Type I Diabetes | Type 1 diabetes mellitus with diabetic autonomic (poly)neuropathy |
| Diabetes mellitus | ICD-10-CM Diagnosis | E10.44 | Type I Diabetes | Type 1 diabetes mellitus with diabetic amyotrophy |
| Diabetes mellitus | ICD-10-CM Diagnosis | E10.49 | Type I Diabetes | Type 1 diabetes mellitus with other diabetic neurological complication |
| Diabetes mellitus | ICD-10-CM Diagnosis | E10.5 | Type I Diabetes | Type 1 diabetes mellitus with circulatory complications |
| Diabetes mellitus | ICD-10-CM Diagnosis | E10.51 | Type I Diabetes | Type 1 diabetes mellitus with diabetic peripheral angiopathy without gangrene |
| Diabetes mellitus | ICD-10-CM Diagnosis | E10.52 | Type I Diabetes | Type 1 diabetes mellitus with diabetic peripheral angiopathy with gangrene |
| Diabetes mellitus | ICD-10-CM Diagnosis | E10.59 | Type I Diabetes | Type 1 diabetes mellitus with other circulatory complications |
| Diabetes mellitus | ICD-10-CM Diagnosis | E10.6 | Type I Diabetes | Type 1 diabetes mellitus with other specified complications |
| Diabetes mellitus | ICD-10-CM Diagnosis | E10.61 | Type I Diabetes | Type 1 diabetes mellitus with diabetic arthropathy |
| Diabetes mellitus | ICD-10-CM Diagnosis | E10.610 | Type I Diabetes | Type 1 diabetes mellitus with diabetic neuropathic arthropathy |
| Diabetes mellitus | ICD-10-CM Diagnosis | E10.618 | Type I Diabetes | Type 1 diabetes mellitus with other diabetic arthropathy |
| Diabetes mellitus | ICD-10-CM Diagnosis | E10.62 | Type I Diabetes | Type 1 diabetes mellitus with skin complications |
| Diabetes mellitus | ICD-10-CM Diagnosis | E10.620 | Type I Diabetes | Type 1 diabetes mellitus with diabetic dermatitis |
| Diabetes mellitus | ICD-10-CM Diagnosis | E10.621 | Type I Diabetes | Type 1 diabetes mellitus with foot ulcer |
| Diabetes mellitus | ICD-10-CM Diagnosis | E10.622 | Type I Diabetes | Type 1 diabetes mellitus with other skin ulcer |
| Diabetes mellitus | ICD-10-CM Diagnosis | E10.628 | Type I Diabetes | Type 1 diabetes mellitus with other skin complications |
| Diabetes mellitus | ICD-10-CM Diagnosis | E10.63 | Type I Diabetes | Type 1 diabetes mellitus with oral complications |
| Diabetes mellitus | ICD-10-CM Diagnosis | E10.630 | Type I Diabetes | Type 1 diabetes mellitus with periodontal disease |
| Diabetes mellitus | ICD-10-CM Diagnosis | E10.638 | Type I Diabetes | Type 1 diabetes mellitus with other oral complications |
| Diabetes mellitus | ICD-10-CM Diagnosis | E10.64 | Type I Diabetes | Type 1 diabetes mellitus with hypoglycemia |
| Diabetes mellitus | ICD-10-CM Diagnosis | E10.641 | Type I Diabetes | Type 1 diabetes mellitus with hypoglycemia with coma |
| Diabetes mellitus | ICD-10-CM Diagnosis | E10.649 | Type I Diabetes | Type 1 diabetes mellitus with hypoglycemia without coma |
| Diabetes mellitus | ICD-10-CM Diagnosis | E10.65 | Type I Diabetes | Type 1 diabetes mellitus with hyperglycemia |
| Diabetes mellitus | ICD-10-CM Diagnosis | E10.69 | Type I Diabetes | Type 1 diabetes mellitus with other specified complication |
| Diabetes mellitus | ICD-10-CM Diagnosis | E10.8 | Type I Diabetes | Type 1 diabetes mellitus with unspecified complications |
| Diabetes mellitus | ICD-10-CM Diagnosis | E10.9 | Type I Diabetes | Type 1 diabetes mellitus without complications |
| Diabetes mellitus | ICD-10-CM Diagnosis | O24.0 | Type I Diabetes | Pre-existing type 1 diabetes mellitus, in pregnancy, childbirth and the puerperium |
| Diabetes mellitus | ICD-10-CM Diagnosis | O24.01 | Type I Diabetes | Pre-existing type 1 diabetes mellitus, in pregnancy |
| Diabetes mellitus | ICD-10-CM Diagnosis | O24.011 | Type I Diabetes | Pre-existing type 1 diabetes mellitus, in pregnancy, first trimester |
| Diabetes mellitus | ICD-10-CM Diagnosis | O24.012 | Type I Diabetes | Pre-existing type 1 diabetes mellitus, in pregnancy, second trimester |
| Diabetes mellitus | ICD-10-CM Diagnosis | O24.013 | Type I Diabetes | Pre-existing type 1 diabetes mellitus, in pregnancy, third trimester |
| Diabetes mellitus | ICD-10-CM Diagnosis | O24.019 | Type I Diabetes | Pre-existing type 1 diabetes mellitus, in pregnancy, unspecified trimester |
| Diabetes mellitus | ICD-10-CM Diagnosis | O24.03 | Type I Diabetes | Pre-existing type 1 diabetes mellitus, in the puerperium |
| Diabetes mellitus | ICD-9-CM Diagnosis | 250.10 | Type II Diabetes | Diabetes with ketoacidosis, type II or unspecified type, not stated as uncontrolled |
| Diabetes mellitus | ICD-9-CM Diagnosis | 250.12 | Type II Diabetes | Diabetes with ketoacidosis, type II or unspecified type, uncontrolled |
| Diabetes mellitus | ICD-9-CM Diagnosis | 250.80 | Type II Diabetes | Diabetes with other specified manifestations, type II or unspecified type, not stated as uncontrolled |
| Diabetes mellitus | ICD-9-CM Diagnosis | 250.82 | Type II Diabetes | Diabetes with other specified manifestations, type II or unspecified type, uncontrolled |
| Diabetes mellitus | ICD-9-CM Diagnosis | 250.90 | Type II Diabetes | Diabetes with unspecified complication, type II or unspecified type, not stated as uncontrolled |
| Diabetes mellitus | ICD-9-CM Diagnosis | 250.92 | Type II Diabetes | Diabetes with unspecified complication, type II or unspecified type, uncontrolled |
| Diabetes mellitus | ICD-10-CM Diagnosis | E11 | Type II Diabetes | Type 2 diabetes mellitus |
| Diabetes mellitus | ICD-10-CM Diagnosis | E11.0 | Type II Diabetes | Type 2 diabetes mellitus with hyperosmolarity |
| Diabetes mellitus | ICD-10-CM Diagnosis | E11.00 | Type II Diabetes | Type 2 diabetes mellitus with hyperosmolarity without nonketotic hyperglycemic-hyperosmolar coma (NKHHC) |
| Diabetes mellitus | ICD-10-CM Diagnosis | E11.01 | Type II Diabetes | Type 2 diabetes mellitus with hyperosmolarity with coma |
| Diabetes mellitus | ICD-10-CM Diagnosis | E11.1 | Type II Diabetes | Type 2 diabetes mellitus with ketoacidosis |
| Diabetes mellitus | ICD-10-CM Diagnosis | E11.10 | Type II Diabetes | Type 2 diabetes mellitus with ketoacidosis without coma |
| Diabetes mellitus | ICD-10-CM Diagnosis | E11.11 | Type II Diabetes | Type 2 diabetes mellitus with ketoacidosis with coma |
| Diabetes mellitus | ICD-10-CM Diagnosis | E11.2 | Type II Diabetes | Type 2 diabetes mellitus with kidney complications |
| Diabetes mellitus | ICD-10-CM Diagnosis | E11.21 | Type II Diabetes | Type 2 diabetes mellitus with diabetic nephropathy |
| Diabetes mellitus | ICD-10-CM Diagnosis | E11.22 | Type II Diabetes | Type 2 diabetes mellitus with diabetic chronic kidney disease |
| Diabetes mellitus | ICD-10-CM Diagnosis | E11.29 | Type II Diabetes | Type 2 diabetes mellitus with other diabetic kidney complication |
| Diabetes mellitus | ICD-10-CM Diagnosis | E11.3 | Type II Diabetes | Type 2 diabetes mellitus with ophthalmic complications |
| Diabetes mellitus | ICD-10-CM Diagnosis | E11.31 | Type II Diabetes | Type 2 diabetes mellitus with unspecified diabetic retinopathy |
| Diabetes mellitus | ICD-10-CM Diagnosis | E11.311 | Type II Diabetes | Type 2 diabetes mellitus with unspecified diabetic retinopathy with macular edema |
| Diabetes mellitus | ICD-10-CM Diagnosis | E11.319 | Type II Diabetes | Type 2 diabetes mellitus with unspecified diabetic retinopathy without macular edema |
| Diabetes mellitus | ICD-10-CM Diagnosis | E11.32 | Type II Diabetes | Type 2 diabetes mellitus with mild nonproliferative diabetic retinopathy |
| Diabetes mellitus | ICD-10-CM Diagnosis | E11.321 | Type II Diabetes | Type 2 diabetes mellitus with mild nonproliferative diabetic retinopathy with macular edema |
| Diabetes mellitus | ICD-10-CM Diagnosis | E11.3211 | Type II Diabetes | Type 2 diabetes mellitus with mild nonproliferative diabetic retinopathy with macular edema, right eye |
| Diabetes mellitus | ICD-10-CM Diagnosis | E11.3212 | Type II Diabetes | Type 2 diabetes mellitus with mild nonproliferative diabetic retinopathy with macular edema, left eye |
| Diabetes mellitus | ICD-10-CM Diagnosis | E11.3213 | Type II Diabetes | Type 2 diabetes mellitus with mild nonproliferative diabetic retinopathy with macular edema, bilateral |
| Diabetes mellitus | ICD-10-CM Diagnosis | E11.3219 | Type II Diabetes | Type 2 diabetes mellitus with mild nonproliferative diabetic retinopathy with macular edema, unspecified eye |
| Diabetes mellitus | ICD-10-CM Diagnosis | E11.329 | Type II Diabetes | Type 2 diabetes mellitus with mild nonproliferative diabetic retinopathy without macular edema |
| Diabetes mellitus | ICD-10-CM Diagnosis | E11.3291 | Type II Diabetes | Type 2 diabetes mellitus with mild nonproliferative diabetic retinopathy without macular edema, right eye |
| Diabetes mellitus | ICD-10-CM Diagnosis | E11.3292 | Type II Diabetes | Type 2 diabetes mellitus with mild nonproliferative diabetic retinopathy without macular edema, left eye |
| Diabetes mellitus | ICD-10-CM Diagnosis | E11.3293 | Type II Diabetes | Type 2 diabetes mellitus with mild nonproliferative diabetic retinopathy without macular edema, bilateral |
| Diabetes mellitus | ICD-10-CM Diagnosis | E11.3299 | Type II Diabetes | Type 2 diabetes mellitus with mild nonproliferative diabetic retinopathy without macular edema, unspecified eye |
| Diabetes mellitus | ICD-10-CM Diagnosis | E11.33 | Type II Diabetes | Type 2 diabetes mellitus with moderate nonproliferative diabetic retinopathy |
| Diabetes mellitus | ICD-10-CM Diagnosis | E11.331 | Type II Diabetes | Type 2 diabetes mellitus with moderate nonproliferative diabetic retinopathy with macular edema |
| Diabetes mellitus | ICD-10-CM Diagnosis | E11.3311 | Type II Diabetes | Type 2 diabetes mellitus with moderate nonproliferative diabetic retinopathy with macular edema, right eye |
| Diabetes mellitus | ICD-10-CM Diagnosis | E11.3312 | Type II Diabetes | Type 2 diabetes mellitus with moderate nonproliferative diabetic retinopathy with macular edema, left eye |
| Diabetes mellitus | ICD-10-CM Diagnosis | E11.3313 | Type II Diabetes | Type 2 diabetes mellitus with moderate nonproliferative diabetic retinopathy with macular edema, bilateral |
| Diabetes mellitus | ICD-10-CM Diagnosis | E11.3319 | Type II Diabetes | Type 2 diabetes mellitus with moderate nonproliferative diabetic retinopathy with macular edema, unspecified eye |
| Diabetes mellitus | ICD-10-CM Diagnosis | E11.339 | Type II Diabetes | Type 2 diabetes mellitus with moderate nonproliferative diabetic retinopathy without macular edema |
| Diabetes mellitus | ICD-10-CM Diagnosis | E11.3391 | Type II Diabetes | Type 2 diabetes mellitus with moderate nonproliferative diabetic retinopathy without macular edema, right eye |
| Diabetes mellitus | ICD-10-CM Diagnosis | E11.3392 | Type II Diabetes | Type 2 diabetes mellitus with moderate nonproliferative diabetic retinopathy without macular edema, left eye |
| Diabetes mellitus | ICD-10-CM Diagnosis | E11.3393 | Type II Diabetes | Type 2 diabetes mellitus with moderate nonproliferative diabetic retinopathy without macular edema, bilateral |
| Diabetes mellitus | ICD-10-CM Diagnosis | E11.3399 | Type II Diabetes | Type 2 diabetes mellitus with moderate nonproliferative diabetic retinopathy without macular edema, unspecified eye |
| Diabetes mellitus | ICD-10-CM Diagnosis | E11.34 | Type II Diabetes | Type 2 diabetes mellitus with severe nonproliferative diabetic retinopathy |
| Diabetes mellitus | ICD-10-CM Diagnosis | E11.341 | Type II Diabetes | Type 2 diabetes mellitus with severe nonproliferative diabetic retinopathy with macular edema |
| Diabetes mellitus | ICD-10-CM Diagnosis | E11.3411 | Type II Diabetes | Type 2 diabetes mellitus with severe nonproliferative diabetic retinopathy with macular edema, right eye |
| Diabetes mellitus | ICD-10-CM Diagnosis | E11.3412 | Type II Diabetes | Type 2 diabetes mellitus with severe nonproliferative diabetic retinopathy with macular edema, left eye |
| Diabetes mellitus | ICD-10-CM Diagnosis | E11.3413 | Type II Diabetes | Type 2 diabetes mellitus with severe nonproliferative diabetic retinopathy with macular edema, bilateral |
| Diabetes mellitus | ICD-10-CM Diagnosis | E11.3419 | Type II Diabetes | Type 2 diabetes mellitus with severe nonproliferative diabetic retinopathy with macular edema, unspecified eye |
| Diabetes mellitus | ICD-10-CM Diagnosis | E11.349 | Type II Diabetes | Type 2 diabetes mellitus with severe nonproliferative diabetic retinopathy without macular edema |
| Diabetes mellitus | ICD-10-CM Diagnosis | E11.3491 | Type II Diabetes | Type 2 diabetes mellitus with severe nonproliferative diabetic retinopathy without macular edema, right eye |
| Diabetes mellitus | ICD-10-CM Diagnosis | E11.3492 | Type II Diabetes | Type 2 diabetes mellitus with severe nonproliferative diabetic retinopathy without macular edema, left eye |
| Diabetes mellitus | ICD-10-CM Diagnosis | E11.3493 | Type II Diabetes | Type 2 diabetes mellitus with severe nonproliferative diabetic retinopathy without macular edema, bilateral |
| Diabetes mellitus | ICD-10-CM Diagnosis | E11.3499 | Type II Diabetes | Type 2 diabetes mellitus with severe nonproliferative diabetic retinopathy without macular edema, unspecified eye |
| Diabetes mellitus | ICD-10-CM Diagnosis | E11.35 | Type II Diabetes | Type 2 diabetes mellitus with proliferative diabetic retinopathy |
| Diabetes mellitus | ICD-10-CM Diagnosis | E11.351 | Type II Diabetes | Type 2 diabetes mellitus with proliferative diabetic retinopathy with macular edema |
| Diabetes mellitus | ICD-10-CM Diagnosis | E11.3511 | Type II Diabetes | Type 2 diabetes mellitus with proliferative diabetic retinopathy with macular edema, right eye |
| Diabetes mellitus | ICD-10-CM Diagnosis | E11.3512 | Type II Diabetes | Type 2 diabetes mellitus with proliferative diabetic retinopathy with macular edema, left eye |
| Diabetes mellitus | ICD-10-CM Diagnosis | E11.3513 | Type II Diabetes | Type 2 diabetes mellitus with proliferative diabetic retinopathy with macular edema, bilateral |
| Diabetes mellitus | ICD-10-CM Diagnosis | E11.3519 | Type II Diabetes | Type 2 diabetes mellitus with proliferative diabetic retinopathy with macular edema, unspecified eye |
| Diabetes mellitus | ICD-10-CM Diagnosis | E11.352 | Type II Diabetes | Type 2 diabetes mellitus with proliferative diabetic retinopathy with traction retinal detachment involving the macula |
| Diabetes mellitus | ICD-10-CM Diagnosis | E11.3521 | Type II Diabetes | Type 2 diabetes mellitus with proliferative diabetic retinopathy with traction retinal detachment involving the macula, right eye |
| Diabetes mellitus | ICD-10-CM Diagnosis | E11.3522 | Type II Diabetes | Type 2 diabetes mellitus with proliferative diabetic retinopathy with traction retinal detachment involving the macula, left eye |
| Diabetes mellitus | ICD-10-CM Diagnosis | E11.3523 | Type II Diabetes | Type 2 diabetes mellitus with proliferative diabetic retinopathy with traction retinal detachment involving the macula, bilateral |
| Diabetes mellitus | ICD-10-CM Diagnosis | E11.3529 | Type II Diabetes | Type 2 diabetes mellitus with proliferative diabetic retinopathy with traction retinal detachment involving the macula, unspecified eye |
| Diabetes mellitus | ICD-10-CM Diagnosis | E11.353 | Type II Diabetes | Type 2 diabetes mellitus with proliferative diabetic retinopathy with traction retinal detachment not involving the macula |
| Diabetes mellitus | ICD-10-CM Diagnosis | E11.3531 | Type II Diabetes | Type 2 diabetes mellitus with proliferative diabetic retinopathy with traction retinal detachment not involving the macula, right eye |
| Diabetes mellitus | ICD-10-CM Diagnosis | E11.3532 | Type II Diabetes | Type 2 diabetes mellitus with proliferative diabetic retinopathy with traction retinal detachment not involving the macula, left eye |
| Diabetes mellitus | ICD-10-CM Diagnosis | E11.3533 | Type II Diabetes | Type 2 diabetes mellitus with proliferative diabetic retinopathy with traction retinal detachment not involving the macula, bilateral |
| Diabetes mellitus | ICD-10-CM Diagnosis | E11.3539 | Type II Diabetes | Type 2 diabetes mellitus with proliferative diabetic retinopathy with traction retinal detachment not involving the macula, unspecified eye |
| Diabetes mellitus | ICD-10-CM Diagnosis | E11.354 | Type II Diabetes | Type 2 diabetes mellitus with proliferative diabetic retinopathy with combined traction retinal detachment and rhegmatogenous retinal detachment |
| Diabetes mellitus | ICD-10-CM Diagnosis | E11.3541 | Type II Diabetes | Type 2 diabetes mellitus with proliferative diabetic retinopathy with combined traction retinal detachment and rhegmatogenous retinal detachment, right eye |
| Diabetes mellitus | ICD-10-CM Diagnosis | E11.3542 | Type II Diabetes | Type 2 diabetes mellitus with proliferative diabetic retinopathy with combined traction retinal detachment and rhegmatogenous retinal detachment, left eye |
| Diabetes mellitus | ICD-10-CM Diagnosis | E11.3543 | Type II Diabetes | Type 2 diabetes mellitus with proliferative diabetic retinopathy with combined traction retinal detachment and rhegmatogenous retinal detachment, bilateral |
| Diabetes mellitus | ICD-10-CM Diagnosis | E11.3549 | Type II Diabetes | Type 2 diabetes mellitus with proliferative diabetic retinopathy with combined traction retinal detachment and rhegmatogenous retinal detachment, unspecified eye |
| Diabetes mellitus | ICD-10-CM Diagnosis | E11.355 | Type II Diabetes | Type 2 diabetes mellitus with stable proliferative diabetic retinopathy |
| Diabetes mellitus | ICD-10-CM Diagnosis | E11.3551 | Type II Diabetes | Type 2 diabetes mellitus with stable proliferative diabetic retinopathy, right eye |
| Diabetes mellitus | ICD-10-CM Diagnosis | E11.3552 | Type II Diabetes | Type 2 diabetes mellitus with stable proliferative diabetic retinopathy, left eye |
| Diabetes mellitus | ICD-10-CM Diagnosis | E11.3553 | Type II Diabetes | Type 2 diabetes mellitus with stable proliferative diabetic retinopathy, bilateral |
| Diabetes mellitus | ICD-10-CM Diagnosis | E11.3559 | Type II Diabetes | Type 2 diabetes mellitus with stable proliferative diabetic retinopathy, unspecified eye |
| Diabetes mellitus | ICD-10-CM Diagnosis | E11.359 | Type II Diabetes | Type 2 diabetes mellitus with proliferative diabetic retinopathy without macular edema |
| Diabetes mellitus | ICD-10-CM Diagnosis | E11.3591 | Type II Diabetes | Type 2 diabetes mellitus with proliferative diabetic retinopathy without macular edema, right eye |
| Diabetes mellitus | ICD-10-CM Diagnosis | E11.3592 | Type II Diabetes | Type 2 diabetes mellitus with proliferative diabetic retinopathy without macular edema, left eye |
| Diabetes mellitus | ICD-10-CM Diagnosis | E11.3593 | Type II Diabetes | Type 2 diabetes mellitus with proliferative diabetic retinopathy without macular edema, bilateral |
| Diabetes mellitus | ICD-10-CM Diagnosis | E11.3599 | Type II Diabetes | Type 2 diabetes mellitus with proliferative diabetic retinopathy without macular edema, unspecified eye |
| Diabetes mellitus | ICD-10-CM Diagnosis | E11.36 | Type II Diabetes | Type 2 diabetes mellitus with diabetic cataract |
| Diabetes mellitus | ICD-10-CM Diagnosis | E11.37 | Type II Diabetes | Type 2 diabetes mellitus with diabetic macular edema, resolved following treatment |
| Diabetes mellitus | ICD-10-CM Diagnosis | E11.37X1 | Type II Diabetes | Type 2 diabetes mellitus with diabetic macular edema, resolved following treatment, right eye |
| Diabetes mellitus | ICD-10-CM Diagnosis | E11.37X2 | Type II Diabetes | Type 2 diabetes mellitus with diabetic macular edema, resolved following treatment, left eye |
| Diabetes mellitus | ICD-10-CM Diagnosis | E11.37X3 | Type II Diabetes | Type 2 diabetes mellitus with diabetic macular edema, resolved following treatment, bilateral |
| Diabetes mellitus | ICD-10-CM Diagnosis | E11.37X9 | Type II Diabetes | Type 2 diabetes mellitus with diabetic macular edema, resolved following treatment, unspecified eye |
| Diabetes mellitus | ICD-10-CM Diagnosis | E11.39 | Type II Diabetes | Type 2 diabetes mellitus with other diabetic ophthalmic complication |
| Diabetes mellitus | ICD-10-CM Diagnosis | E11.4 | Type II Diabetes | Type 2 diabetes mellitus with neurological complications |
| Diabetes mellitus | ICD-10-CM Diagnosis | E11.40 | Type II Diabetes | Type 2 diabetes mellitus with diabetic neuropathy, unspecified |
| Diabetes mellitus | ICD-10-CM Diagnosis | E11.41 | Type II Diabetes | Type 2 diabetes mellitus with diabetic mononeuropathy |
| Diabetes mellitus | ICD-10-CM Diagnosis | E11.42 | Type II Diabetes | Type 2 diabetes mellitus with diabetic polyneuropathy |
| Diabetes mellitus | ICD-10-CM Diagnosis | E11.43 | Type II Diabetes | Type 2 diabetes mellitus with diabetic autonomic (poly)neuropathy |
| Diabetes mellitus | ICD-10-CM Diagnosis | E11.44 | Type II Diabetes | Type 2 diabetes mellitus with diabetic amyotrophy |
| Diabetes mellitus | ICD-10-CM Diagnosis | E11.49 | Type II Diabetes | Type 2 diabetes mellitus with other diabetic neurological complication |
| Diabetes mellitus | ICD-10-CM Diagnosis | E11.5 | Type II Diabetes | Type 2 diabetes mellitus with circulatory complications |
| Diabetes mellitus | ICD-10-CM Diagnosis | E11.51 | Type II Diabetes | Type 2 diabetes mellitus with diabetic peripheral angiopathy without gangrene |
| Diabetes mellitus | ICD-10-CM Diagnosis | E11.52 | Type II Diabetes | Type 2 diabetes mellitus with diabetic peripheral angiopathy with gangrene |
| Diabetes mellitus | ICD-10-CM Diagnosis | E11.59 | Type II Diabetes | Type 2 diabetes mellitus with other circulatory complications |
| Diabetes mellitus | ICD-10-CM Diagnosis | E11.6 | Type II Diabetes | Type 2 diabetes mellitus with other specified complications |
| Diabetes mellitus | ICD-10-CM Diagnosis | E11.61 | Type II Diabetes | Type 2 diabetes mellitus with diabetic arthropathy |
| Diabetes mellitus | ICD-10-CM Diagnosis | E11.610 | Type II Diabetes | Type 2 diabetes mellitus with diabetic neuropathic arthropathy |
| Diabetes mellitus | ICD-10-CM Diagnosis | E11.618 | Type II Diabetes | Type 2 diabetes mellitus with other diabetic arthropathy |
| Diabetes mellitus | ICD-10-CM Diagnosis | E11.62 | Type II Diabetes | Type 2 diabetes mellitus with skin complications |
| Diabetes mellitus | ICD-10-CM Diagnosis | E11.620 | Type II Diabetes | Type 2 diabetes mellitus with diabetic dermatitis |
| Diabetes mellitus | ICD-10-CM Diagnosis | E11.621 | Type II Diabetes | Type 2 diabetes mellitus with foot ulcer |
| Diabetes mellitus | ICD-10-CM Diagnosis | E11.622 | Type II Diabetes | Type 2 diabetes mellitus with other skin ulcer |
| Diabetes mellitus | ICD-10-CM Diagnosis | E11.628 | Type II Diabetes | Type 2 diabetes mellitus with other skin complications |
| Diabetes mellitus | ICD-10-CM Diagnosis | E11.63 | Type II Diabetes | Type 2 diabetes mellitus with oral complications |
| Diabetes mellitus | ICD-10-CM Diagnosis | E11.630 | Type II Diabetes | Type 2 diabetes mellitus with periodontal disease |
| Diabetes mellitus | ICD-10-CM Diagnosis | E11.638 | Type II Diabetes | Type 2 diabetes mellitus with other oral complications |
| Diabetes mellitus | ICD-10-CM Diagnosis | E11.64 | Type II Diabetes | Type 2 diabetes mellitus with hypoglycemia |
| Diabetes mellitus | ICD-10-CM Diagnosis | E11.641 | Type II Diabetes | Type 2 diabetes mellitus with hypoglycemia with coma |
| Diabetes mellitus | ICD-10-CM Diagnosis | E11.649 | Type II Diabetes | Type 2 diabetes mellitus with hypoglycemia without coma |
| Diabetes mellitus | ICD-10-CM Diagnosis | E11.65 | Type II Diabetes | Type 2 diabetes mellitus with hyperglycemia |
| Diabetes mellitus | ICD-10-CM Diagnosis | E11.69 | Type II Diabetes | Type 2 diabetes mellitus with other specified complication |
| Diabetes mellitus | ICD-10-CM Diagnosis | E11.8 | Type II Diabetes | Type 2 diabetes mellitus with unspecified complications |
| Diabetes mellitus | ICD-10-CM Diagnosis | E11.9 | Type II Diabetes | Type 2 diabetes mellitus without complications |
| Diabetes mellitus | ICD-10-CM Diagnosis | O24.1 | Type II Diabetes | Pre-existing type 2 diabetes mellitus, in pregnancy, childbirth and the puerperium |
| Diabetes mellitus | ICD-10-CM Diagnosis | O24.11 | Type II Diabetes | Pre-existing type 2 diabetes mellitus, in pregnancy |
| Diabetes mellitus | ICD-10-CM Diagnosis | O24.111 | Type II Diabetes | Pre-existing type 2 diabetes mellitus, in pregnancy, first trimester |
| Diabetes mellitus | ICD-10-CM Diagnosis | O24.112 | Type II Diabetes | Pre-existing type 2 diabetes mellitus, in pregnancy, second trimester |
| Diabetes mellitus | ICD-10-CM Diagnosis | O24.113 | Type II Diabetes | Pre-existing type 2 diabetes mellitus, in pregnancy, third trimester |
| Diabetes mellitus | ICD-10-CM Diagnosis | O24.119 | Type II Diabetes | Pre-existing type 2 diabetes mellitus, in pregnancy, unspecified trimester |
| Diabetes mellitus | ICD-10-CM Diagnosis | O24.13 | Type II Diabetes | Pre-existing type 2 diabetes mellitus, in the puerperium |

International Classification of Diseases, Tenth Revision, Clinical Modification.

d) Kidney disease diagnosis

| **Criterion Name** | **Code Type** | **Code** | **Full description** |
| --- | --- | --- | --- |
| Acute Renal Failure | ICD-10-CM Diagnosis | N17.9 | Acute kidney failure, unspecified |
| Acute Renal Failure | ICD-10-CM Diagnosis | N17.8 | Other acute kidney failure |
| Acute Renal Failure | ICD-10-CM Diagnosis | N17.2 | Acute kidney failure with medullary necrosis |
| Acute Renal Failure | ICD-10-CM Diagnosis | N17.1 | Acute kidney failure with acute cortical necrosis |
| Acute Renal Failure | ICD-10-CM Diagnosis | N17.0 | Acute kidney failure with tubular necrosis |
| Acute Renal Failure | ICD-10-CM Diagnosis | N17 | Acute kidney failure |
| Dialysis | ICD-10-CM Diagnosis | Z99.2 | Dependence on renal dialysis |
| Kidney Transplant | ICD-10-CM Diagnosis | T86.11 | Kidney transplant rejection |
| Kidney Transplant | ICD-10-CM Diagnosis | T86.12 | Kidney transplant failure |
| Kidney Transplant | ICD-10-CM Diagnosis | T86.13 | Kidney transplant infection |
| Kidney Transplant | ICD-10-CM Diagnosis | T86.10 | Unspecified complication of kidney transplant |
| Kidney Transplant | ICD-10-CM Diagnosis | T86.19 | Other complication of kidney transplant |
| Kidney Transplant | ICD-10-CM Diagnosis | Z94.0 | Kidney transplant status |
| Kidney Transplant | ICD-10-CM Diagnosis | T86.1 | Complications of kidney transplant |
| Kidney Transplant | ICD-10-CM Diagnosis | Z48.22 |  |

International Classification of Diseases, Tenth Revision, Clinical Modification.

e) Kidney disease procedures

| **Criterion Name** | **Code Type** | **Code** |
| --- | --- | --- |
| Dialysis | CPT | 4052F |
| Dialysis | CPT | 4053F |
| Dialysis | CPT | 4054F |
| Dialysis | CPT | 4055F |
| Dialysis | CPT | 90935 |
| Dialysis | CPT | 90937 |
| Dialysis | CPT | 90945 |
| Dialysis | CPT | 90947 |
| Dialysis | CPT | 90965 |
| Dialysis | CPT | 90966 |
| Dialysis | CPT | 90969 |
| Dialysis | CPT | 90970 |
| Dialysis | CPT | 90999 |
| Dialysis | CPT | 99512 |
| Dialysis | CPT | G0257 |
| Dialysis | HCPCS | G0322 |
| Dialysis | HCPCS | G0323 |
| Dialysis | HCPCS | G0326 |
| Dialysis | HCPCS | G0327 |
| Dialysis | HCPCS | G8081 |
| Dialysis | HCPCS | G8082 |
| Dialysis | HCPCS | G8085 |
| Dialysis | HCPCS | G8714 |
| Dialysis | HCPCS | G8715 |
| Dialysis | HCPCS | G8956 |
| Dialysis | HCPCS | G9264 |
| Dialysis | HCPCS | G9265 |
| Dialysis | HCPCS | G9266 |
| Dialysis | HCPCS | S9335 |
| Dialysis | HCPCS | S9339 |
| Dialysis | HCPCS | G0491 |
| Dialysis | ICD-10-PCS | 5A1D00Z |
| Dialysis | ICD-10-PCS | 5A1D60Z |
| Dialysis | ICD-10-PCS | 5A1D70Z |
| Dialysis | ICD-10-PCS | 5A1D80Z |
| Dialysis | ICD-10-PCS | 5A1D90Z |
| Dialysis | ICD-10-PCS | 3E1M39Z |
| Kidney Transplant | ICD-10-PCS | 0TY00Z0 |
| Kidney Transplant | ICD-10-PCS | 0TY00Z1 |
| Kidney Transplant | ICD-10-PCS | 0TY00Z2 |
| Kidney Transplant | ICD-10-PCS | 0TY10Z0 |
| Kidney Transplant | ICD-10-PCS | 0TY10Z1 |
| Kidney Transplant | ICD-10-PCS | 0TY10Z2 |
| Kidney Transplant | CPT | 50360 |
| Kidney Transplant | CPT | 50365 |
| Kidney Transplant | CPT | 50370 |
| Kidney Transplant | CPT | 50380 |
| Kidney Transplant | HCPCS | S2065 |

CPT, Current Procedural Terminology; HCPCS, Healthcare Common Procedure Coding System; ICD-10-PCS, International Classification of Diseases, Tenth Revision, Procedure Coding System.

e) Venous thromboembolism (VTE) diagnosis

| **Criterion Name** | **Code Type** | **Code** | **Code Description** | **Full description** |
| --- | --- | --- | --- | --- |
| VTE | ICD-9-CM Diagnosis | 415.1 | VTE | Pulmonary embolism and infarction |
| VTE | ICD-9-CM Diagnosis | 415.11 | VTE | Iatrogenic pulmonary embolism and infarction |
| VTE | ICD-9-CM Diagnosis | 415.12 | VTE | Septic pulmonary embolism |
| VTE | ICD-9-CM Diagnosis | 415.13 | VTE | Saddle embolus of pulmonary artery |
| VTE | ICD-9-CM Diagnosis | 415.19 | VTE | Other pulmonary embolism and infarction |
| VTE | ICD-9-CM Diagnosis | 416.2 | VTE | Chronic pulmonary embolism |
| VTE | ICD-9-CM Diagnosis | 437.6 | VTE | Nonpyogenic thrombosis of intracranial venous sinus |
| VTE | ICD-9-CM Diagnosis | 453.1 | VTE | Thrombophlebitis migrans |
| VTE | ICD-9-CM Diagnosis | 453.2 | VTE | Other venous embolism and thrombosis of inferior vena cava |
| VTE | ICD-9-CM Diagnosis | 453.3 | VTE | Other venous embolism and thrombosis of renal vein |
| VTE | ICD-9-CM Diagnosis | 453.4 | VTE | Acute venous embolism and thrombosis of deep vessels of lower extremity |
| VTE | ICD-9-CM Diagnosis | 453.40 | VTE | Acute venous embolism and thrombosis of unspecified deep vessels of lower extremity |
| VTE | ICD-9-CM Diagnosis | 453.41 | VTE | Acute venous embolism and thrombosis of deep vessels of proximal lower extremity |
| VTE | ICD-9-CM Diagnosis | 453.42 | VTE | Acute venous embolism and thrombosis of deep vessels of distal lower extremity |
| VTE | ICD-9-CM Diagnosis | 453.5 | VTE | Chronic venous embolism and thrombosis of deep vessels of lower extremity |
| VTE | ICD-9-CM Diagnosis | 453.50 | VTE | Chronic venous embolism and thrombosis of unspecified deep vessels of lower extremity |
| VTE | ICD-9-CM Diagnosis | 453.51 | VTE | Chronic venous embolism and thrombosis of deep vessels of proximal lower extremity |
| VTE | ICD-9-CM Diagnosis | 453.52 | VTE | Chronic venous embolism and thrombosis of deep vessels of distal lower extremity |
| VTE | ICD-9-CM Diagnosis | 453.6 | VTE | Venous embolism and thrombosis of superficial vessels of lower extremity |
| VTE | ICD-9-CM Diagnosis | 453.7 | VTE | Chronic venous embolism and thrombosis of other specified vessels |
| VTE | ICD-9-CM Diagnosis | 453.71 | VTE | Chronic venous embolism and thrombosis of superficial veins of upper extremity |
| VTE | ICD-9-CM Diagnosis | 453.72 | VTE | Chronic venous embolism and thrombosis of deep veins of upper extremity |
| VTE | ICD-9-CM Diagnosis | 453.73 | VTE | Chronic venous embolism and thrombosis of upper extremity, unspecified |
| VTE | ICD-9-CM Diagnosis | 453.74 | VTE | Chronic venous embolism and thrombosis of axillary veins |
| VTE | ICD-9-CM Diagnosis | 453.75 | VTE | Chronic venous embolism and thrombosis of subclavian veins |
| VTE | ICD-9-CM Diagnosis | 453.76 | VTE | Chronic venous embolism and thrombosis of internal jugular veins |
| VTE | ICD-9-CM Diagnosis | 453.77 | VTE | Chronic venous embolism and thrombosis of other thoracic veins |
| VTE | ICD-9-CM Diagnosis | 453.79 | VTE | Chronic venous embolism and thrombosis of other specified veins |
| VTE | ICD-9-CM Diagnosis | 453.81 | VTE | Acute venous embolism and thrombosis of superficial veins of upper extremity |
| VTE | ICD-9-CM Diagnosis | 453.82 | VTE | Acute venous embolism and thrombosis of deep veins of upper extremity |
| VTE | ICD-9-CM Diagnosis | 453.83 | VTE | Acute venous embolism and thrombosis of upper extremity, unspecified |
| VTE | ICD-9-CM Diagnosis | 453.84 | VTE | Acute venous embolism and thrombosis of axillary veins |
| VTE | ICD-9-CM Diagnosis | 453.85 | VTE | Acute venous embolism and thrombosis of subclavian veins |
| VTE | ICD-9-CM Diagnosis | 453.86 | VTE | Acute venous embolism and thrombosis of internal jugular veins |
| VTE | ICD-9-CM Diagnosis | 453.87 | VTE | Acute venous embolism and thrombosis of other thoracic veins |
| VTE | ICD-9-CM Diagnosis | 453.89 | VTE | Acute venous embolism and thrombosis of other specified veins |
| VTE | ICD-9-CM Diagnosis | 453.9 | VTE | Other venous embolism and thrombosis of unspecified site |
| VTE | ICD-10-CM Diagnosis | I26 | VTE | Pulmonary embolism |
| VTE | ICD-10-CM Diagnosis | I26.0 | VTE | Pulmonary embolism with acute cor pulmonale |
| VTE | ICD-10-CM Diagnosis | I26.01 | VTE | Septic pulmonary embolism with acute cor pulmonale |
| VTE | ICD-10-CM Diagnosis | I26.02 | VTE | Saddle embolus of pulmonary artery with acute cor pulmonale |
| VTE | ICD-10-CM Diagnosis | I26.09 | VTE | Other pulmonary embolism with acute cor pulmonale |
| VTE | ICD-10-CM Diagnosis | I26.9 | VTE | Pulmonary embolism without acute cor pulmonale |
| VTE | ICD-10-CM Diagnosis | I26.90 | VTE | Septic pulmonary embolism without acute cor pulmonale |
| VTE | ICD-10-CM Diagnosis | I26.92 | VTE | Saddle embolus of pulmonary artery without acute cor pulmonale |
| VTE | ICD-10-CM Diagnosis | I26.93 | VTE | Single subsegmental pulmonary embolism without acute cor pulmonale |
| VTE | ICD-10-CM Diagnosis | I26.94 | VTE | Multiple subsegmental pulmonary emboli without acute cor pulmonale |
| VTE | ICD-10-CM Diagnosis | I26.99 | VTE | Other pulmonary embolism without acute cor pulmonale |
| VTE | ICD-10-CM Diagnosis | I27.82 | VTE | Chronic pulmonary embolism |
| VTE | ICD-10-CM Diagnosis | I67.6 | VTE | Nonpyogenic thrombosis of intracranial venous system |
| VTE | ICD-10-CM Diagnosis | I80 | VTE | Phlebitis and thrombophlebitis |
| VTE | ICD-10-CM Diagnosis | I80.2 | VTE | Phlebitis and thrombophlebitis of other and unspecified deep vessels of lower extremities |
| VTE | ICD-10-CM Diagnosis | I80.20 | VTE | Phlebitis and thrombophlebitis of unspecified deep vessels of lower extremities |
| VTE | ICD-10-CM Diagnosis | I80.201 | VTE | Phlebitis and thrombophlebitis of unspecified deep vessels of right lower extremity |
| VTE | ICD-10-CM Diagnosis | I80.202 | VTE | Phlebitis and thrombophlebitis of unspecified deep vessels of left lower extremity |
| VTE | ICD-10-CM Diagnosis | I80.203 | VTE | Phlebitis and thrombophlebitis of unspecified deep vessels of lower extremities, bilateral |
| VTE | ICD-10-CM Diagnosis | I80.209 | VTE | Phlebitis and thrombophlebitis of unspecified deep vessels of unspecified lower extremity |
| VTE | ICD-10-CM Diagnosis | I80.21 | VTE | Phlebitis and thrombophlebitis of iliac vein |
| VTE | ICD-10-CM Diagnosis | I80.211 | VTE | Phlebitis and thrombophlebitis of right iliac vein |
| VTE | ICD-10-CM Diagnosis | I80.212 | VTE | Phlebitis and thrombophlebitis of left iliac vein |
| VTE | ICD-10-CM Diagnosis | I80.213 | VTE | Phlebitis and thrombophlebitis of iliac vein, bilateral |
| VTE | ICD-10-CM Diagnosis | I80.219 | VTE | Phlebitis and thrombophlebitis of unspecified iliac vein |
| VTE | ICD-10-CM Diagnosis | I80.29 | VTE | Phlebitis and thrombophlebitis of other deep vessels of lower extremities |
| VTE | ICD-10-CM Diagnosis | I80.291 | VTE | Phlebitis and thrombophlebitis of other deep vessels of right lower extremity |
| VTE | ICD-10-CM Diagnosis | I80.292 | VTE | Phlebitis and thrombophlebitis of other deep vessels of left lower extremity |
| VTE | ICD-10-CM Diagnosis | I80.293 | VTE | Phlebitis and thrombophlebitis of other deep vessels of lower extremity, bilateral |
| VTE | ICD-10-CM Diagnosis | I80.299 | VTE | Phlebitis and thrombophlebitis of other deep vessels of unspecified lower extremity |
| VTE | ICD-10-CM Diagnosis | I80.3 | VTE | Phlebitis and thrombophlebitis of lower extremities, unspecified |
| VTE | ICD-10-CM Diagnosis | I80.8 | VTE | Phlebitis and thrombophlebitis of other sites |
| VTE | ICD-10-CM Diagnosis | I80.9 | VTE | Phlebitis and thrombophlebitis of unspecified site |
| VTE | ICD-10-CM Diagnosis | I82.1 | VTE | Thrombophlebitis migrans |
| VTE | ICD-10-CM Diagnosis | I82.2 | VTE | Embolism and thrombosis of vena cava and other thoracic veins |
| VTE | ICD-10-CM Diagnosis | I82.21 | VTE | Embolism and thrombosis of superior vena cava |
| VTE | ICD-10-CM Diagnosis | I82.210 | VTE | Acute embolism and thrombosis of superior vena cava |
| VTE | ICD-10-CM Diagnosis | I82.211 | VTE | Chronic embolism and thrombosis of superior vena cava |
| VTE | ICD-10-CM Diagnosis | I82.22 | VTE | Embolism and thrombosis of inferior vena cava |
| VTE | ICD-10-CM Diagnosis | I82.220 | VTE | Acute embolism and thrombosis of inferior vena cava |
| VTE | ICD-10-CM Diagnosis | I82.221 | VTE | Chronic embolism and thrombosis of inferior vena cava |
| VTE | ICD-10-CM Diagnosis | I82.29 | VTE | Embolism and thrombosis of other thoracic veins |
| VTE | ICD-10-CM Diagnosis | I82.290 | VTE | Acute embolism and thrombosis of other thoracic veins |
| VTE | ICD-10-CM Diagnosis | I82.291 | VTE | Chronic embolism and thrombosis of other thoracic veins |
| VTE | ICD-10-CM Diagnosis | I82.3 | VTE | Embolism and thrombosis of renal vein |
| VTE | ICD-10-CM Diagnosis | I82.4 | VTE | Acute embolism and thrombosis of deep veins of lower extremity |
| VTE | ICD-10-CM Diagnosis | I82.40 | VTE | Acute embolism and thrombosis of unspecified deep veins of lower extremity |
| VTE | ICD-10-CM Diagnosis | I82.401 | VTE | Acute embolism and thrombosis of unspecified deep veins of right lower extremity |
| VTE | ICD-10-CM Diagnosis | I82.402 | VTE | Acute embolism and thrombosis of unspecified deep veins of left lower extremity |
| VTE | ICD-10-CM Diagnosis | I82.403 | VTE | Acute embolism and thrombosis of unspecified deep veins of lower extremity, bilateral |
| VTE | ICD-10-CM Diagnosis | I82.409 | VTE | Acute embolism and thrombosis of unspecified deep veins of unspecified lower extremity |
| VTE | ICD-10-CM Diagnosis | I82.41 | VTE | Acute embolism and thrombosis of femoral vein |
| VTE | ICD-10-CM Diagnosis | I82.411 | VTE | Acute embolism and thrombosis of right femoral vein |
| VTE | ICD-10-CM Diagnosis | I82.412 | VTE | Acute embolism and thrombosis of left femoral vein |
| VTE | ICD-10-CM Diagnosis | I82.413 | VTE | Acute embolism and thrombosis of femoral vein, bilateral |
| VTE | ICD-10-CM Diagnosis | I82.419 | VTE | Acute embolism and thrombosis of unspecified femoral vein |
| VTE | ICD-10-CM Diagnosis | I82.42 | VTE | Acute embolism and thrombosis of iliac vein |
| VTE | ICD-10-CM Diagnosis | I82.421 | VTE | Acute embolism and thrombosis of right iliac vein |
| VTE | ICD-10-CM Diagnosis | I82.422 | VTE | Acute embolism and thrombosis of left iliac vein |
| VTE | ICD-10-CM Diagnosis | I82.423 | VTE | Acute embolism and thrombosis of iliac vein, bilateral |
| VTE | ICD-10-CM Diagnosis | I82.429 | VTE | Acute embolism and thrombosis of unspecified iliac vein |
| VTE | ICD-10-CM Diagnosis | I82.43 | VTE | Acute embolism and thrombosis of popliteal vein |
| VTE | ICD-10-CM Diagnosis | I82.431 | VTE | Acute embolism and thrombosis of right popliteal vein |
| VTE | ICD-10-CM Diagnosis | I82.432 | VTE | Acute embolism and thrombosis of left popliteal vein |
| VTE | ICD-10-CM Diagnosis | I82.433 | VTE | Acute embolism and thrombosis of popliteal vein, bilateral |
| VTE | ICD-10-CM Diagnosis | I82.439 | VTE | Acute embolism and thrombosis of unspecified popliteal vein |
| VTE | ICD-10-CM Diagnosis | I82.44 | VTE | Acute embolism and thrombosis of tibial vein |
| VTE | ICD-10-CM Diagnosis | I82.441 | VTE | Acute embolism and thrombosis of right tibial vein |
| VTE | ICD-10-CM Diagnosis | I82.442 | VTE | Acute embolism and thrombosis of left tibial vein |
| VTE | ICD-10-CM Diagnosis | I82.443 | VTE | Acute embolism and thrombosis of tibial vein, bilateral |
| VTE | ICD-10-CM Diagnosis | I82.449 | VTE | Acute embolism and thrombosis of unspecified tibial vein |
| VTE | ICD-10-CM Diagnosis | I82.49 | VTE | Acute embolism and thrombosis of other specified deep vein of lower extremity |
| VTE | ICD-10-CM Diagnosis | I82.491 | VTE | Acute embolism and thrombosis of other specified deep vein of right lower extremity |
| VTE | ICD-10-CM Diagnosis | I82.492 | VTE | Acute embolism and thrombosis of other specified deep vein of left lower extremity |
| VTE | ICD-10-CM Diagnosis | I82.493 | VTE | Acute embolism and thrombosis of other specified deep vein of lower extremity, bilateral |
| VTE | ICD-10-CM Diagnosis | I82.499 | VTE | Acute embolism and thrombosis of other specified deep vein of unspecified lower extremity |
| VTE | ICD-10-CM Diagnosis | I82.4Y | VTE | Acute embolism and thrombosis of unspecified deep veins of proximal lower extremity |
| VTE | ICD-10-CM Diagnosis | I82.4Y1 | VTE | Acute embolism and thrombosis of unspecified deep veins of right proximal lower extremity |
| VTE | ICD-10-CM Diagnosis | I82.4Y2 | VTE | Acute embolism and thrombosis of unspecified deep veins of left proximal lower extremity |
| VTE | ICD-10-CM Diagnosis | I82.4Y3 | VTE | Acute embolism and thrombosis of unspecified deep veins of proximal lower extremity, bilateral |
| VTE | ICD-10-CM Diagnosis | I82.4Y9 | VTE | Acute embolism and thrombosis of unspecified deep veins of unspecified proximal lower extremity |
| VTE | ICD-10-CM Diagnosis | I82.4Z | VTE | Acute embolism and thrombosis of unspecified deep veins of distal lower extremity |
| VTE | ICD-10-CM Diagnosis | I82.4Z1 | VTE | Acute embolism and thrombosis of unspecified deep veins of right distal lower extremity |
| VTE | ICD-10-CM Diagnosis | I82.4Z2 | VTE | Acute embolism and thrombosis of unspecified deep veins of left distal lower extremity |
| VTE | ICD-10-CM Diagnosis | I82.4Z3 | VTE | Acute embolism and thrombosis of unspecified deep veins of distal lower extremity, bilateral |
| VTE | ICD-10-CM Diagnosis | I82.4Z9 | VTE | Acute embolism and thrombosis of unspecified deep veins of unspecified distal lower extremity |
| VTE | ICD-10-CM Diagnosis | I82.5 | VTE | Chronic embolism and thrombosis of deep veins of lower extremity |
| VTE | ICD-10-CM Diagnosis | I82.50 | VTE | Chronic embolism and thrombosis of unspecified deep veins of lower extremity |
| VTE | ICD-10-CM Diagnosis | I82.501 | VTE | Chronic embolism and thrombosis of unspecified deep veins of right lower extremity |
| VTE | ICD-10-CM Diagnosis | I82.502 | VTE | Chronic embolism and thrombosis of unspecified deep veins of left lower extremity |
| VTE | ICD-10-CM Diagnosis | I82.503 | VTE | Chronic embolism and thrombosis of unspecified deep veins of lower extremity, bilateral |
| VTE | ICD-10-CM Diagnosis | I82.509 | VTE | Chronic embolism and thrombosis of unspecified deep veins of unspecified lower extremity |
| VTE | ICD-10-CM Diagnosis | I82.59 | VTE | Chronic embolism and thrombosis of other specified deep vein of lower extremity |
| VTE | ICD-10-CM Diagnosis | I82.591 | VTE | Chronic embolism and thrombosis of other specified deep vein of right lower extremity |
| VTE | ICD-10-CM Diagnosis | I82.592 | VTE | Chronic embolism and thrombosis of other specified deep vein of left lower extremity |
| VTE | ICD-10-CM Diagnosis | I82.593 | VTE | Chronic embolism and thrombosis of other specified deep vein of lower extremity, bilateral |
| VTE | ICD-10-CM Diagnosis | I82.599 | VTE | Chronic embolism and thrombosis of other specified deep vein of unspecified lower extremity |
| VTE | ICD-10-CM Diagnosis | I82.5Y | VTE | Chronic embolism and thrombosis of unspecified deep veins of proximal lower extremity |
| VTE | ICD-10-CM Diagnosis | I82.5Y1 | VTE | Chronic embolism and thrombosis of unspecified deep veins of right proximal lower extremity |
| VTE | ICD-10-CM Diagnosis | I82.5Y2 | VTE | Chronic embolism and thrombosis of unspecified deep veins of left proximal lower extremity |
| VTE | ICD-10-CM Diagnosis | I82.5Y3 | VTE | Chronic embolism and thrombosis of unspecified deep veins of proximal lower extremity, bilateral |
| VTE | ICD-10-CM Diagnosis | I82.5Y9 | VTE | Chronic embolism and thrombosis of unspecified deep veins of unspecified proximal lower extremity |
| VTE | ICD-10-CM Diagnosis | I82.6 | VTE | Acute embolism and thrombosis of veins of upper extremity |
| VTE | ICD-10-CM Diagnosis | I82.60 | VTE | Acute embolism and thrombosis of unspecified veins of upper extremity |
| VTE | ICD-10-CM Diagnosis | I82.601 | VTE | Acute embolism and thrombosis of unspecified veins of right upper extremity |
| VTE | ICD-10-CM Diagnosis | I82.602 | VTE | Acute embolism and thrombosis of unspecified veins of left upper extremity |
| VTE | ICD-10-CM Diagnosis | I82.603 | VTE | Acute embolism and thrombosis of unspecified veins of upper extremity, bilateral |
| VTE | ICD-10-CM Diagnosis | I82.609 | VTE | Acute embolism and thrombosis of unspecified veins of unspecified upper extremity |
| VTE | ICD-10-CM Diagnosis | I82.61 | VTE | Acute embolism and thrombosis of superficial veins of upper extremity |
| VTE | ICD-10-CM Diagnosis | I82.611 | VTE | Acute embolism and thrombosis of superficial veins of right upper extremity |
| VTE | ICD-10-CM Diagnosis | I82.612 | VTE | Acute embolism and thrombosis of superficial veins of left upper extremity |
| VTE | ICD-10-CM Diagnosis | I82.613 | VTE | Acute embolism and thrombosis of superficial veins of upper extremity, bilateral |
| VTE | ICD-10-CM Diagnosis | I82.619 | VTE | Acute embolism and thrombosis of superficial veins of unspecified upper extremity |
| VTE | ICD-10-CM Diagnosis | I82.62 | VTE | Acute embolism and thrombosis of deep veins of upper extremity |
| VTE | ICD-10-CM Diagnosis | I82.621 | VTE | Acute embolism and thrombosis of deep veins of right upper extremity |
| VTE | ICD-10-CM Diagnosis | I82.622 | VTE | Acute embolism and thrombosis of deep veins of left upper extremity |
| VTE | ICD-10-CM Diagnosis | I82.623 | VTE | Acute embolism and thrombosis of deep veins of upper extremity, bilateral |
| VTE | ICD-10-CM Diagnosis | I82.629 | VTE | Acute embolism and thrombosis of deep veins of unspecified upper extremity |
| VTE | ICD-10-CM Diagnosis | I82.7 | VTE | Chronic embolism and thrombosis of veins of upper extremity |
| VTE | ICD-10-CM Diagnosis | I82.70 | VTE | Chronic embolism and thrombosis of unspecified veins of upper extremity |
| VTE | ICD-10-CM Diagnosis | I82.701 | VTE | Chronic embolism and thrombosis of unspecified veins of right upper extremity |
| VTE | ICD-10-CM Diagnosis | I82.702 | VTE | Chronic embolism and thrombosis of unspecified veins of left upper extremity |
| VTE | ICD-10-CM Diagnosis | I82.703 | VTE | Chronic embolism and thrombosis of unspecified veins of upper extremity, bilateral |
| VTE | ICD-10-CM Diagnosis | I82.709 | VTE | Chronic embolism and thrombosis of unspecified veins of unspecified upper extremity |
| VTE | ICD-10-CM Diagnosis | I82.71 | VTE | Chronic embolism and thrombosis of superficial veins of upper extremity |
| VTE | ICD-10-CM Diagnosis | I82.711 | VTE | Chronic embolism and thrombosis of superficial veins of right upper extremity |
| VTE | ICD-10-CM Diagnosis | I82.712 | VTE | Chronic embolism and thrombosis of superficial veins of left upper extremity |
| VTE | ICD-10-CM Diagnosis | I82.713 | VTE | Chronic embolism and thrombosis of superficial veins of upper extremity, bilateral |
| VTE | ICD-10-CM Diagnosis | I82.719 | VTE | Chronic embolism and thrombosis of superficial veins of unspecified upper extremity |
| VTE | ICD-10-CM Diagnosis | I82.72 | VTE | Chronic embolism and thrombosis of deep veins of upper extremity |
| VTE | ICD-10-CM Diagnosis | I82.721 | VTE | Chronic embolism and thrombosis of deep veins of right upper extremity |
| VTE | ICD-10-CM Diagnosis | I82.722 | VTE | Chronic embolism and thrombosis of deep veins of left upper extremity |
| VTE | ICD-10-CM Diagnosis | I82.723 | VTE | Chronic embolism and thrombosis of deep veins of upper extremity, bilateral |
| VTE | ICD-10-CM Diagnosis | I82.729 | VTE | Chronic embolism and thrombosis of deep veins of unspecified upper extremity |
| VTE | ICD-10-CM Diagnosis | I82.8 | VTE | Embolism and thrombosis of other specified veins |
| VTE | ICD-10-CM Diagnosis | I82.81 | VTE | Embolism and thrombosis of superficial veins of lower extremities |
| VTE | ICD-10-CM Diagnosis | I82.811 | VTE | Embolism and thrombosis of superficial veins of right lower extremity |
| VTE | ICD-10-CM Diagnosis | I82.812 | VTE | Embolism and thrombosis of superficial veins of left lower extremity |
| VTE | ICD-10-CM Diagnosis | I82.813 | VTE | Embolism and thrombosis of superficial veins of lower extremities, bilateral |
| VTE | ICD-10-CM Diagnosis | I82.819 | VTE | Embolism and thrombosis of superficial veins of unspecified lower extremity |
| VTE | ICD-10-CM Diagnosis | I82.89 | VTE | Embolism and thrombosis of other specified veins |
| VTE | ICD-10-CM Diagnosis | I82.890 | VTE | Acute embolism and thrombosis of other specified veins |
| VTE | ICD-10-CM Diagnosis | I82.891 | VTE | Chronic embolism and thrombosis of other specified veins |
| VTE | ICD-10-CM Diagnosis | I82.9 | VTE | Embolism and thrombosis of unspecified vein |
| VTE | ICD-10-CM Diagnosis | I82.90 | VTE | Acute embolism and thrombosis of unspecified vein |
| VTE | ICD-10-CM Diagnosis | I82.91 | VTE | Chronic embolism and thrombosis of unspecified vein |
| VTE | ICD-10-CM Diagnosis | I82.A | VTE | Embolism and thrombosis of axillary vein |
| VTE | ICD-10-CM Diagnosis | I82.A1 | VTE | Acute embolism and thrombosis of axillary vein |
| VTE | ICD-10-CM Diagnosis | I82.A11 | VTE | Acute embolism and thrombosis of right axillary vein |
| VTE | ICD-10-CM Diagnosis | I82.A12 | VTE | Acute embolism and thrombosis of left axillary vein |
| VTE | ICD-10-CM Diagnosis | I82.A13 | VTE | Acute embolism and thrombosis of axillary vein, bilateral |
| VTE | ICD-10-CM Diagnosis | I82.A19 | VTE | Acute embolism and thrombosis of unspecified axillary vein |
| VTE | ICD-10-CM Diagnosis | I82.A2 | VTE | Chronic embolism and thrombosis of axillary vein |
| VTE | ICD-10-CM Diagnosis | I82.A21 | VTE | Chronic embolism and thrombosis of right axillary vein |
| VTE | ICD-10-CM Diagnosis | I82.A22 | VTE | Chronic embolism and thrombosis of left axillary vein |
| VTE | ICD-10-CM Diagnosis | I82.A23 | VTE | Chronic embolism and thrombosis of axillary vein, bilateral |
| VTE | ICD-10-CM Diagnosis | I82.A29 | VTE | Chronic embolism and thrombosis of unspecified axillary vein |
| VTE | ICD-10-CM Diagnosis | I82.B | VTE | Embolism and thrombosis of subclavian vein |
| VTE | ICD-10-CM Diagnosis | I82.B1 | VTE | Acute embolism and thrombosis of subclavian vein |
| VTE | ICD-10-CM Diagnosis | I82.B11 | VTE | Acute embolism and thrombosis of right subclavian vein |
| VTE | ICD-10-CM Diagnosis | I82.B12 | VTE | Acute embolism and thrombosis of left subclavian vein |
| VTE | ICD-10-CM Diagnosis | I82.B13 | VTE | Acute embolism and thrombosis of subclavian vein, bilateral |
| VTE | ICD-10-CM Diagnosis | I82.B19 | VTE | Acute embolism and thrombosis of unspecified subclavian vein |
| VTE | ICD-10-CM Diagnosis | I82.B2 | VTE | Chronic embolism and thrombosis of subclavian vein |
| VTE | ICD-10-CM Diagnosis | I82.B21 | VTE | Chronic embolism and thrombosis of right subclavian vein |
| VTE | ICD-10-CM Diagnosis | I82.B22 | VTE | Chronic embolism and thrombosis of left subclavian vein |
| VTE | ICD-10-CM Diagnosis | I82.B23 | VTE | Chronic embolism and thrombosis of subclavian vein, bilateral |
| VTE | ICD-10-CM Diagnosis | I82.B29 | VTE | Chronic embolism and thrombosis of unspecified subclavian vein |
| VTE | ICD-10-CM Diagnosis | I82.C | VTE | Embolism and thrombosis of internal jugular vein |
| VTE | ICD-10-CM Diagnosis | I82.C1 | VTE | Acute embolism and thrombosis of internal jugular vein |
| VTE | ICD-10-CM Diagnosis | I82.C11 | VTE | Acute embolism and thrombosis of right internal jugular vein |
| VTE | ICD-10-CM Diagnosis | I82.C12 | VTE | Acute embolism and thrombosis of left internal jugular vein |
| VTE | ICD-10-CM Diagnosis | I82.C13 | VTE | Acute embolism and thrombosis of internal jugular vein, bilateral |
| VTE | ICD-10-CM Diagnosis | I82.C19 | VTE | Acute embolism and thrombosis of unspecified internal jugular vein |
| VTE | ICD-10-CM Diagnosis | I82.C2 | VTE | Chronic embolism and thrombosis of internal jugular vein |
| VTE | ICD-10-CM Diagnosis | I82.C21 | VTE | Chronic embolism and thrombosis of right internal jugular vein |
| VTE | ICD-10-CM Diagnosis | I82.C22 | VTE | Chronic embolism and thrombosis of left internal jugular vein |
| VTE | ICD-10-CM Diagnosis | I82.C23 | VTE | Chronic embolism and thrombosis of internal jugular vein, bilateral |
| VTE | ICD-10-CM Diagnosis | I82.C29 | VTE | Chronic embolism and thrombosis of unspecified internal jugular vein |

ICD-9-CM, International Classification of Diseases, Ninth Revision, Clinical Modification; ICD-10-CM, International Classification of Diseases, Tenth Revision, Clinical Modification.
