# Supplementary figures and images for: Real-world treatment patterns and clinical outcomes for inpatients with COVID-19 in the US from September 2020 to February 2021
Source: PLoS One. 2021 Dec 28;16(12):e0261707. doi: 10.1371/journal.pone.0261707 (PMC8714107; doi:10.1371/journal.pone.0261707)

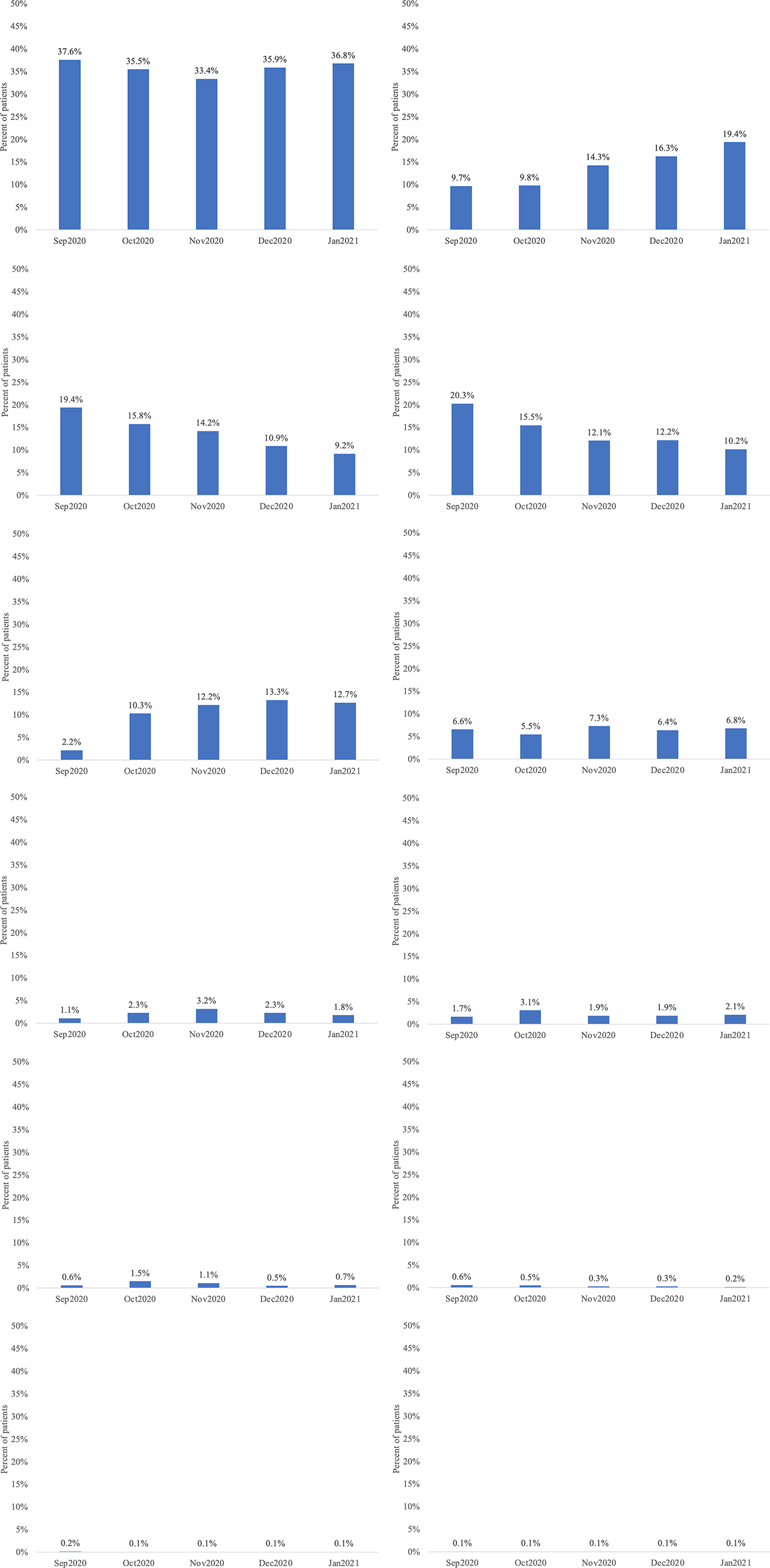

Supplement: S1 Fig — a) Dexamethasone. b) Dexamethasone + Remdesivir. c) Azithromycin. d) Azithromycin + Dexamethasone. e) Remdesivir. f) Azithromycin + Dexamethasone + Remdesivir. g) Azithromycin + Remdesivir. h) Convalescent Plasma + Dexamethasone + Remdesivir. i) Convalescent Plasma. j) Hydroxychloroquine. k) Azithromycin + Hydroxychloroquine. l) Tocilizumab. (TIF) [file pone.0261707.s003.tif]

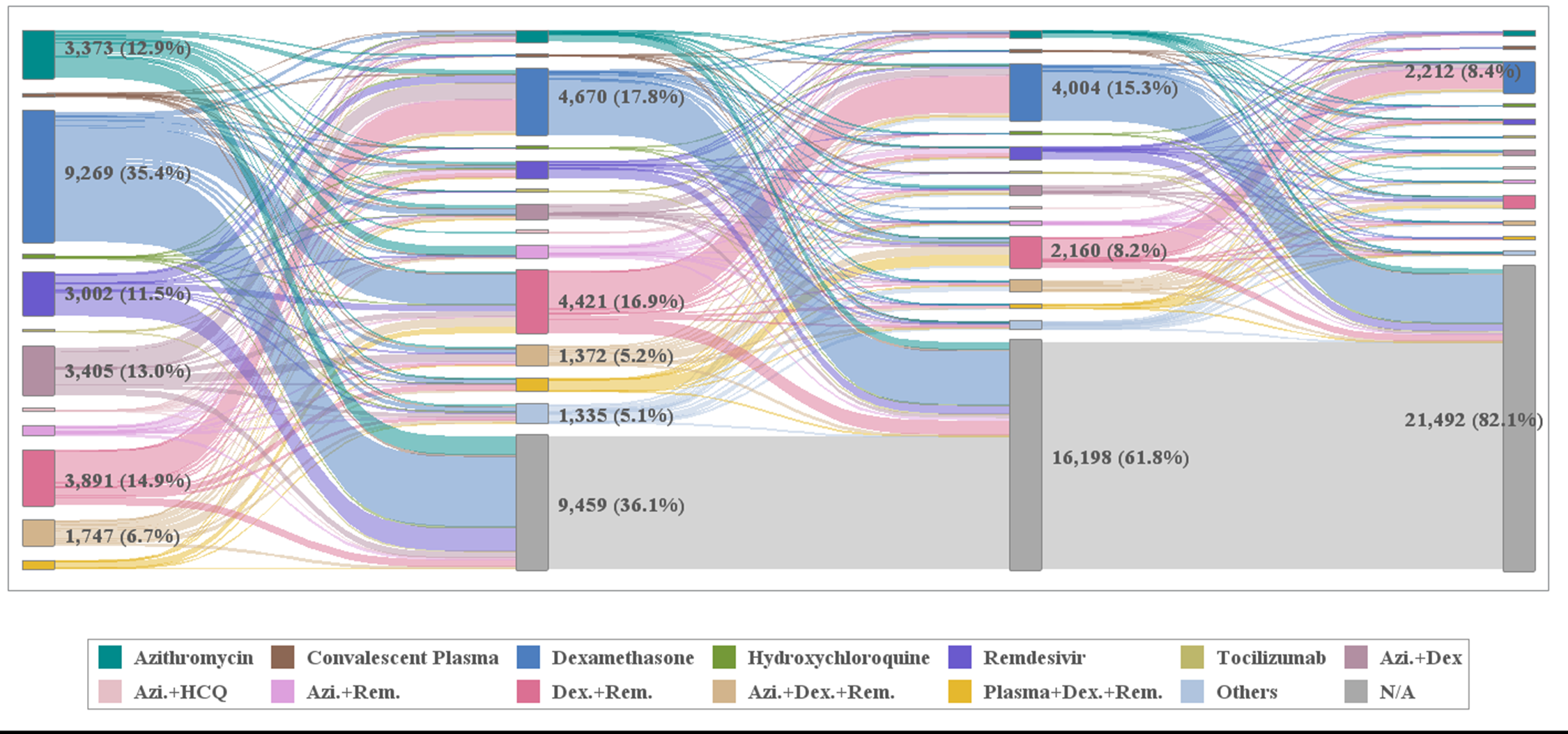

Supplement: S2 Fig — Azi, azithromycin; Dex, dexamethasone; HCQ, hydroxychloroquine; NA, not available; Rem, remdesivir. (TIF) [file pone.0261707.s004.tif]
